# Topoisomerase 3β knockout mice show transcriptional and behavioural impairments associated with neurogenesis and synaptic plasticity

**DOI:** 10.1038/s41467-020-16884-4

**Published:** 2020-06-19

**Authors:** Yuyoung Joo, Yutong Xue, Yue Wang, Ross A. McDevitt, Nirnath Sah, Simone Bossi, Shuaikun Su, Seung Kyu Lee, Wei Peng, Aoji Xie, Yongqing Zhang, Yi Ding, Wai Lim Ku, Soumita Ghosh, Kenneth Fishbein, Weiping Shen, Richard Spencer, Kevin Becker, Keji Zhao, Mark P. Mattson, Henriette van Praag, Alexei Sharov, Weidong Wang

**Affiliations:** 1 Laboratory of Genetics and Genomics, National Institute on Aging, National Institutes of Health, Baltimore, MD 21224 USA; 2 Neuroscience, National Institute on Aging, National Institutes of Health, Baltimore, MD 21224 USA; 3 The Comparative Medicine Section, National Institute on Aging, National Institutes of Health, Baltimore, MD 21224 USA; 4 Laboratory of Epigenome Biology, National Heart, Lung, and Blood Institute, National Institutes of Health, Bethesda, MD 20892 USA; 5 Clinical Investigation, National Institute on Aging, National Institutes of Health, Baltimore, MD 21224 USA; 6 Present Address: Brain Institute and Charles E. Schmidt College of Medicine, Jupiter, FL 33458 USA

**Keywords:** Schizophrenia, Autism spectrum disorders

## Abstract

Topoisomerase 3β (Top3β) is the only dual-activity topoisomerase in animals that can change topology for both DNA and RNA, and facilitate transcription on DNA and translation on mRNAs. Top3β mutations have been linked to schizophrenia, autism, epilepsy, and cognitive impairment. Here we show that Top3β knockout mice exhibit behavioural phenotypes related to psychiatric disorders and cognitive impairment. The mice also display impairments in hippocampal neurogenesis and synaptic plasticity. Notably, the brains of the mutant mice exhibit impaired global neuronal activity-dependent transcription in response to fear conditioning stress, and the affected genes include many with known neuronal functions. Our data suggest that Top3β is essential for normal brain function, and that defective neuronal activity-dependent transcription may be a mechanism by which Top3β deletion causes cognitive impairment and psychiatric disorders.

## Abstract (web summary)

Topoisomerase 3β (Top3β) solves topological stress in DNA or RNA metabolism and its mutations are linked to mental disorders. Here, the authors describe transcriptional and behavioural impairments in Top3β-knockout mice and show that these are linked to impaired neurogenesis and synaptic plasticity.

## Introduction

Topoisomerase 3β (Top3β), a Type IA topoisomerase, has recently drawn increased interest because it is the only one in animals with dual-activity—capable of changing the topology of not only DNA, but also RNA^1^. Emerging evidence shows that Top3β forms a conserved complex with TDRD3 (Tudor domain containing-3), and this complex can function in both DNA- and RNA-based transactions. For example, Top3β has been proposed to be recruited by TDRD3 to promoters of several genes, including c-myc, to reduce R-loops and stimulate transcription in cultured mammalian cell lines^2^. This proposal is supported by reduced transcription and/or increased R-loops in TDRD3-Knockdown cells or null mice. Because there is no similar evidence from Top3β-Knockdown or knockout cells or animals, whether Top3β is required for transcription by reducing R-loops remains unknown. In addition, Top3β-TDRD3 complex can interact with the RNA-binding protein FMRP (Fragile-X mental retardation protein) to facilitate mRNA translation and synapse formation^1,3,4^; and to work with the RNA-induced silencing complex to promote heterochromatin formation and silencing of genes and transposons^5^.

The finding that Top3β interacts with FMRP suggests that Top3β mutations may cause mental disorders similar to Fragile-X syndrome, which is the known leading cause of autism. Indeed, Top3β is frequently deleted in the 22q11.2 distal deletion syndrome, whose features include mental retardation and cognitive dysfunction^6^. Moreover, smaller genomic deletions and de novo single-nucleotide variants have been identified that specifically link Top3β mutations to schizophrenia, autism, epilepsy, intellectual disability, and cognitive impairment^3,4,7–10^. However, whether and how Top3β mutation is causal for these disorders remains unclear. Neither Top3β nor TDRD3 have yet been shown to directly affect transcription or translation of genes important for neuronal function and mental health. In contrast, two other topoisomerases, Top1 and Top2β, are required for normal transcription of genes related to autism, cognitive function, neuronal-early response (NER)^11–13^, and neuronal survival^14^. In particular, Top2β, a Type II topoisomerase, preferentially binds promoters of transcriptionally active genes^14^, and is critical for regulated gene activation^15^ (see review^12^), including neuronal activity-dependent transcription (NADT) of several NER genes^13^. Because neuronal activity can activate transcription of thousands of mRNAs and enhancer RNAs (eRNAs)^16^, this process likely produces a large increase of genome-wide topological constraints within a very short period of time. We hypothesize that resolving these constraints in a timely manner may require additional topoisomerases, including Top3β.

To test this hypothesis and also to investigate whether Top3β deficiency can cause brain dysfunction, we analyzed a Top3β-knockout (KO) mouse model^17^. These mice display shortened life-span^17^, autoimmunity^18^, and abnormal synapse formation^1^; but have not been analyzed for emotional and cognitive functions, nor for transcriptional regulation. Here we show that Top3β-KO mice exhibit several behavior phenotypes related to psychiatric disorders and cognitive impairment, which are associated with abnormal adult neurogenesis and synaptic transmission. Notably, the brains of the mutant mice exhibit impaired NADT, which has been observed in schizophrenia and autism^19^; and the affected genes include many that are critical for synaptic and behavioral neuroplasticity. Our data suggest that Top3β is crucial for normal brain function; and that disruption of NADT could be one mechanism by which Top3β deletion causes mental and cognitive disorders.

## Results

### Top3β-KO mice exhibit abnormal anxiety and fear behaviors

**Fig. 1:**
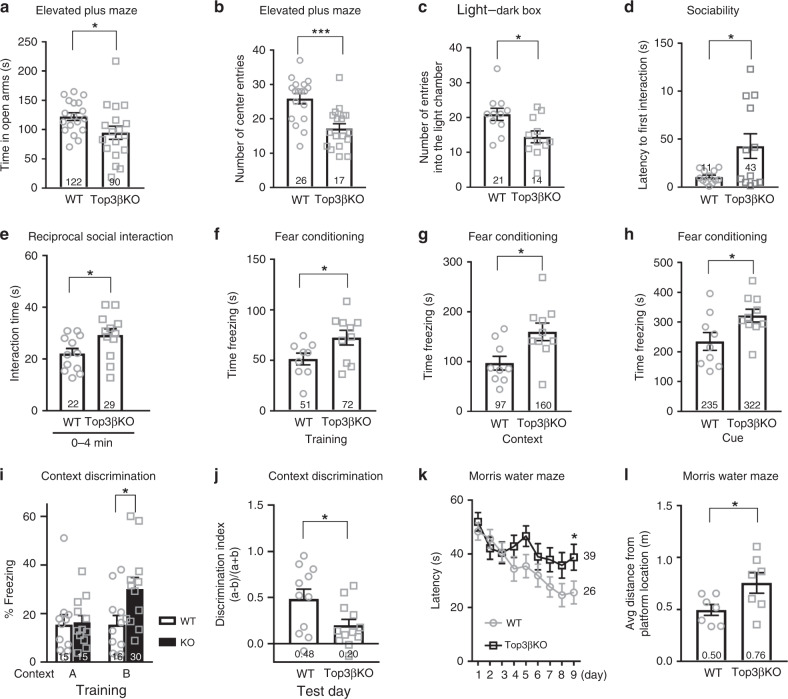
Top3β-KO mice have higher anxiety and defective hippocampus-dependent cognitive functions. **a**, **b** Top3β-KO mice displayed increased anxiety behavior in elevated maze test, as evidenced by reduced staying time (**a**) and number of entries in light arm (**b**) (18 mice/group). **c** Top3β-KO mice exhibited increased anxiety behavior in light-dark box test, as shown by fewer entries into the light box (11 mice/group). **d**, **e** Top3β-KO mice showed abnormal behavior in sociability test, as revealed by increased latency to initiate the first interaction with the other mouse in three-chamber sociability test (**d**), and increased nose-to-nose sniffing interaction in reciprocal social interaction test (**e**) (12 mice per group). **f**–**h** Top3β-KO mice displayed increased fear behaviors, as revealed by longer freezing time in training (**f**), context (**g**), and cue (**h**) test of the FC test (9 WT and 10 KO). The FC paradigm is in (Supplementary Fig. 1a). **i**, **j** Top3β-KO mice exhibited impaired ability to discriminate between a shock-paired context (A) and a neutral context (B). The paradigm is in Supplementary Fig. 1b. During training, Top3β-KO mice exhibited a significant longer freezing time when placed in context B, unlike the WT mice that showed similar short freezing time in both contexts (**i**). In the test day, Top3β-KO mice exhibited reduced discrimination index (**j**), as defined by a ratio in difference in freezing time between context A and B vs. the total freezing time. 11 WT and 12 KO mice were used. **k, l** Top3β-KO mice exhibited reduced learning and memory behaviors in water maze test, as revealed by longer time in finding the hidden platform (**k**), and explored longer average distance to the position of the platform (**l**) (7 mice/group). Two-tail Student *t* test was used for (**a**–**h**, **j**–l). Two-way ANOVA test was used for (**i**) in statistical analysis. *P*-value (**a**) 0.0414, (**b**) 0.0002, (**c**) 0.0149, (**d**) 0.0217, (**e**) 0.0369, (**f**) 0.0416, (**g**) 0.0131, (**h**) 0.0282, (**i**) 0.0136, (**j**) 0.0323, (**k**) 0.0455, (**l**) 0.0389. *P*-values < 0.05, 0.01, and 0.001 are marked as: *, **, ***, respectively. Data are presented as mean values ± SEM. Source data are provided as a Source Data file.

We subjected Top3β-KO mice^17^ to a panel of behavior tests. Compared to wild-type (WT) mice, in the elevated-plus maze, Top3β-KO mice spent significantly less time in the open arms (Fig. 1a), and had fewer entries to the center (Fig. 1b). In the light-dark box test, Top3β-KO mice entered the light box significantly fewer times than did WT mice (Fig. 1c). Together, these results indicate that Top3β-KO mice have greater generalized anxiety level, a phenotype prevalent in schizophrenia patients^20,21^.

In the three-chamber sociability test, Top3β-KO mice showed increased latency before the first investigation with the target mouse (Fig. 1d). In the free social interaction test, they exhibited longer nose-to-nose sniffing time in the initial 4 min of interactions with the other mice (Fig. 1e). These data suggest that Top3β-KO mice have abnormal social interactions, a hallmark of autism^22^.

Next, we performed a fear conditioning (FC) test (Supplementary Fig. 1a), which evaluates the extent to which mice can learn and remember the context (test chamber) or an auditory cue that are associated with an aversive stimulus (electric shock) by training. During the training phase, Top3β-KO mice exhibited longer freezing times (complete absence of motion) (Fig. 1f), indicating increased fear behaviors. Notably, in both contextual and cue tests, Top3β-KO mice displayed significantly longer freezing times (*p* < 0.05) (Fig. 1g, h), suggesting that they have enhanced memory to FC, which has been observed in some mouse models of schizophrenia^23^. The data support the link between Top3β deletion and the disorder.

### Top3β-KO mice have defective hippocampus-dependent cognition

Conditioned fear memory is influenced by hippocampus function^24^, and Top3β mRNA is highly expressed in hippocampus [http://mouse.brain-map.org]. This prompted us to perform two tests that examine hippocampus-dependent cognitive function. One is context discrimination test, measuring the ability of the mice to distinguish two contexts (A and B) that are paired and unpaired with electric shocks, respectively (Supplementary Fig. 1b)^25^. During the initial training to electric shocks, all mice showed similar short freezing time in context A prior to the shock (Fig. 1i). Notably, when placed in context B 4 h later, Top3β-KO mice froze significantly longer than in context A, in contrast to wild-type mice that show short freezing time comparable to that of pre-shock freezing in context A, indicating that Top3β-KO mice are defective in discriminating the two contexts. In the test day, both Top3β-KO and WT mice displayed significantly shorter freezing time in context B than that in A (Supplementary Fig. 1c). However, the discrimination index, which is defined by the difference in freezing time between A and B vs. the total freezing time, was significantly lower in Top3β-KO (Fig. 1j). The data are consistent with the results from the training day and suggest that Top3β inactivation impairs hippocampus-dependent cognitive function. Moreover, Top3β-KO displayed longer freezing time in both contexts (Supplementary Fig. 1c), supporting the findings from FC test that these mice have heightened conditioned fear.

The 2nd test is Morris water maze, which measures the ability of the mice to learn and remember the location of an escape platform hidden in the water. Top3β-KO mice had a longer latency to find the platform over days of training than WT mice (Fig. 1k), indicating defective learning. Moreover, when the platform was removed for a 60-s probe trial 4 h after the last training session, the KO mice showed impaired retention of the spatial memory, because they explored in areas further away from the platform, as measured by average distance to the platform (Fig. 1l). Since impaired learning and memory has been observed frequently in schizophrenia^26^, autism, and dementia, the data support the human genetic data that Top3β mutations may cause psychiatric and cognitive disorders.

Top3β-KO mice exhibit normal behaviors in other tests (Supplementary Fig. 2a-k). These include two tests in which schizophrenia or autism mouse models show abnormal behaviors: prepulse inhibition^27,28^, and olfactory function^29^, indicating that Top3β-KO mice have overlapping but not identical phenotypes as the other disease models.

### Top3β-KO mice have abnormal hippocampal synaptic plasticity

**Fig. 2:**
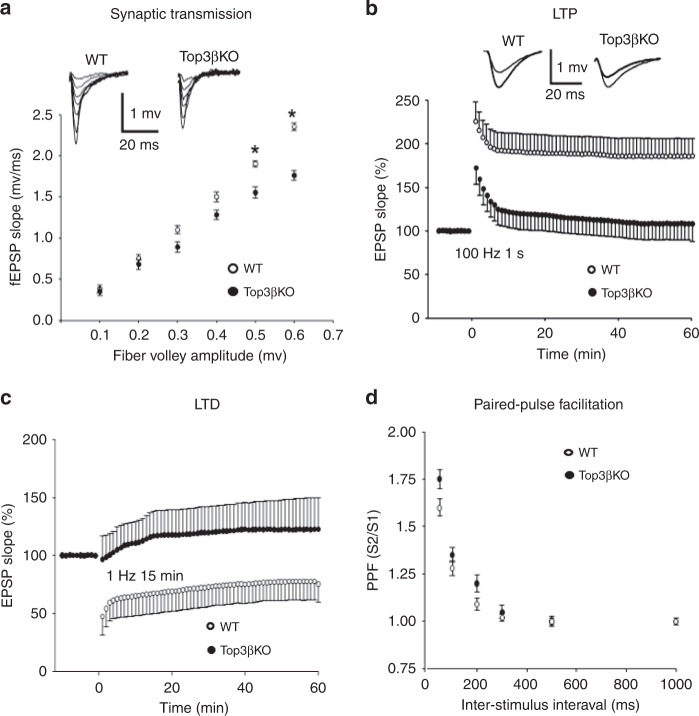
Top3β-KO mice have impaired hippocampal synaptic transmission and plasticity. **a** Top3β-KO mice had lower input/output (I/O) curves at higher stimulation intensity in the neuronal strength test, as shown by decreased amplitude of maximal EPSPs at the two highest stimulation points. 6 slices from 4 WT and 5 slices from 4 KO mice were analyzed. One-tail Student's *t* test was performed for (**a** and **d**). **b** Top3β-KO mice showed defective synaptic plasticity in LTP assay, as revealed by lower amplitude (EPSPs) than that of the WT mice in response to a high-frequency stimulus. Top3β-KO mice displayed significantly lower LTP (EPSP slope) compared to WT mice (114 ± 15 % vs. 185 ± 20%, *p* < 0.01). 9 slices from 5 WT and 5 slices from 4 KO mice were analyzed. One-way ANOVA test was performed for (**b**, **c**). **c** Top3β-KO mice showed no obvious depression in the amplitude responding to a low-frequency stimulus in LTD assay, in contrast to the WT mice that showed strong depression (131 ± 24 vs. 69 ± 16%, *p* < 0.05). 5 slices from 5 WT and 6 slices from 4 KO mice were analyzed. **d** Top3β mutant and WT mice displayed similar amplitude in a short-term plasticity assay, Paired-pulse facilitation (PPF). 6 slices from 4 WT and 6 slices from 6 KO mice were analyzed. Data are presented as mean values ± SEM. *P*-values < 0.05 is marked as*.

The abnormal behaviors above suggest that Top3β-KO mice have impaired hippocampal synaptic functions^30^. Field recordings from CA1 neurons in hippocampal slices revealed that Top3β-KO mice had significantly reduced input/output (I/O) curves at higher stimulation intensities (Fig. 2a), suggesting that hippocampal neurons of Top3β-KO mice have reduced basal synaptic transmission. In the long-term potentiation (LTP) analysis, Top3β-KO mice displayed significantly lower LTP (EPSP slope) (Fig. 2b), in response to a high-frequency stimulation. In the long-term depression (LTD) assay, Top3β-KO mice showed no depression of the EPSP slope in response to a low-frequency stimulus, in contrast to the WT mice which exhibited robust LTD (Fig. 2c). There was no difference in paired-pulse facilitation (PPF) in Top3β-KO and WT mice (Fig. 2d), suggesting that Top3β deficiency does not affect glutamate release from presynaptic terminals. Together, these data suggest that Top3β-KO mice have defective synaptic transmission and plasticity in hippocampal neurons, which could contribute to the learning and memory defects observed in the behavior assays^31,32^.

### Top3β-KO mice have defective adult hippocampal neurogenesis

**Fig. 3:**
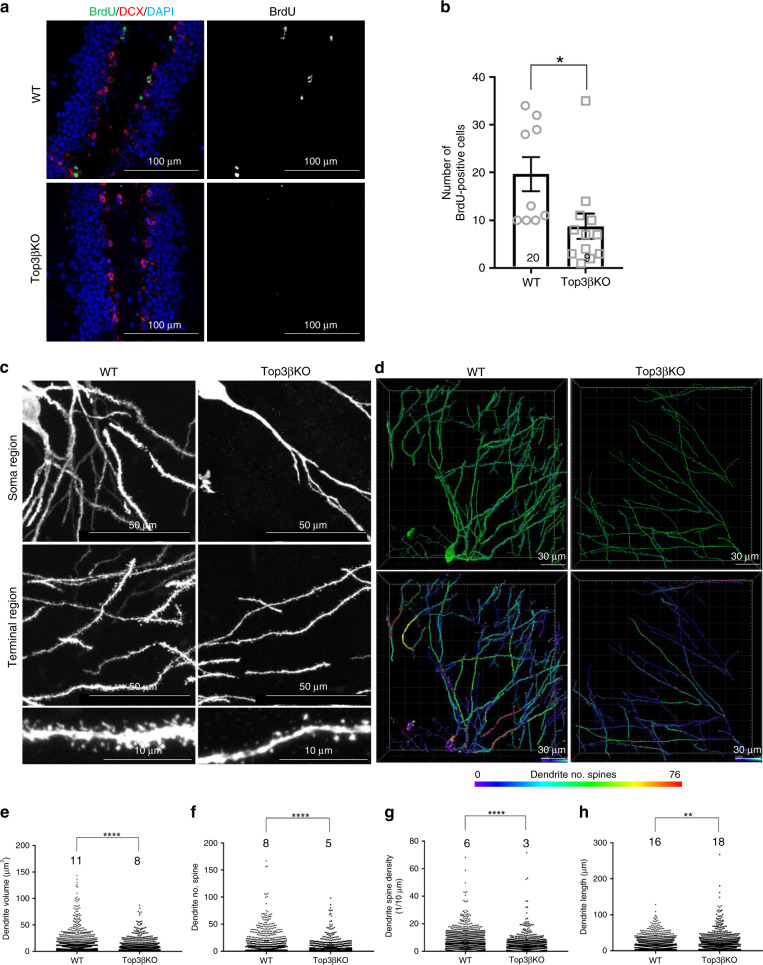
Top3β-KO mice have defective adult neurogenesis in hippocampal neurons. **a**, **b** Immunostaining (**a**) and quantification (**b**) show that Top3β-KO mice have reduced number of BrdU-labeled adult neural stem cells (green) in hippocampus. The graph shows the average number of BrdU postitive cells per slice. DCX (doublecortin) is co-stained as a marker for newborn neurons. 4 slices from each mouse with 3 mice per each group were analyzed. **c**–**h** Representative images of GFP-labeled adult-born neurons (**c**, **d**), and their quantification of different morphological features (**e**–**h**) show that those from Top3β-KO mice have lower complexity. The images visualized and analyzed by Bitplane IMARIS software (Oxford instruments) are shown (d), which use color code to illustrate the difference in number of spines per dendrite between WT and Top3β-KO mice. The warmer color represents higher spine numbers. Graphs of quantifications include: dendrite volume (**e**), spine number per dendrite (**f**), spine density (number/10 μm of dendrite length) (**g**), and dendrite length (**h**). 6 slices from WT and 7 slices from KO were analyzed (*n* = 5 per group). Data are presented as mean values ± SEM. *P*-values < 0.05, 0.01, 0.001, and 0.0001 are marked as: *, **, ***, and ****, respectively. Two-tail Student's *t* test was performed for statistical analysis. *P*-value (**b**) 0.0210, (**e**) <0.0001, (**f**) <0.0001, (**g**) < 0.0001, (**h**) 0.0039. Source data are provided as a Source Data file, which describes detailed calculations including *p*-values.

We next examined hippocampal adult neurogenesis in Top3β-KO mice, which is important for synaptic plasticity involved in spatial learning and memory, and mood^33,34^. We found that the number of BrdU-labeled adult neural stem cells was reduced by about 50% in the dentate gyrus (DG) of hippocampus of Top3β-KO mice (Fig. 3a, b). We also cultured the adult neural stem cells isolated from the hippocampus of Top3β-KO mice, and found that they are severely defective in proliferation and die within 7 days, in contrast to those from WT mice that grow normally (Supplementary Fig. 3a). We then analyzed the morphology of the newly generated neurons in the DG of hippocampus of Top3β-KO mice 1 month after injection into the DG of retrovirus expressing green fluorescent protein (GFP) to label dividing progenitor cells. We found that the new neurons exhibited overall lower complexity in Top3β-KO mice (Fig. 3c, d), with smaller dendrite volume (Fig. 3e), reduced number and density of spines (Fig. 3f, g), and longer dendrite lengths (Fig. 3h). Impaired adult hippocampal neurogenesis might contribute to mental and cognitive dysfunction in individuals with a Top3β mutation^33^.

Ventricular enlargement is a common feature of schizophrenia^35^. MRI imaging analyses showed that the average percentage of ventricle volume in Top3β-KO mice is increased, but this increase did not reach statistical significance (Supplementary Fig. 3b, c).

### Top3β-KO mice have defective transcription in response to FC

Next, we studied whether Top3β resembles Top2β in facilitating NADT of 4 NER genes, *Arc*, *Fos*, *Egr1*, and *Npas4*, which are important in anxiety, learning and memory, and synaptic function^36,37^; and have also been associated with schizophrenia (EGR1)^38^. We used FC training paradigm (Supplementary Fig. 1a) to stimulate neuronal activity because this stimulus can induce Top2β-mediated DSBs (indicative of strong topological constraint)^13^. We measured transcription in mouse whole brains using RNA polymerase II (Pol II) ChIP-seq and total RNA-seq. We mainly relied on the first approach because it directly measures transcription, and is particularly sensitive to defects of topoisomerases, as polymerase progression depends on topoisomerases to resolve topological stress. Conversely, the latter approach detects levels of total RNAs, which reflect not only transcription, but also degradation.

**Fig. 4:**
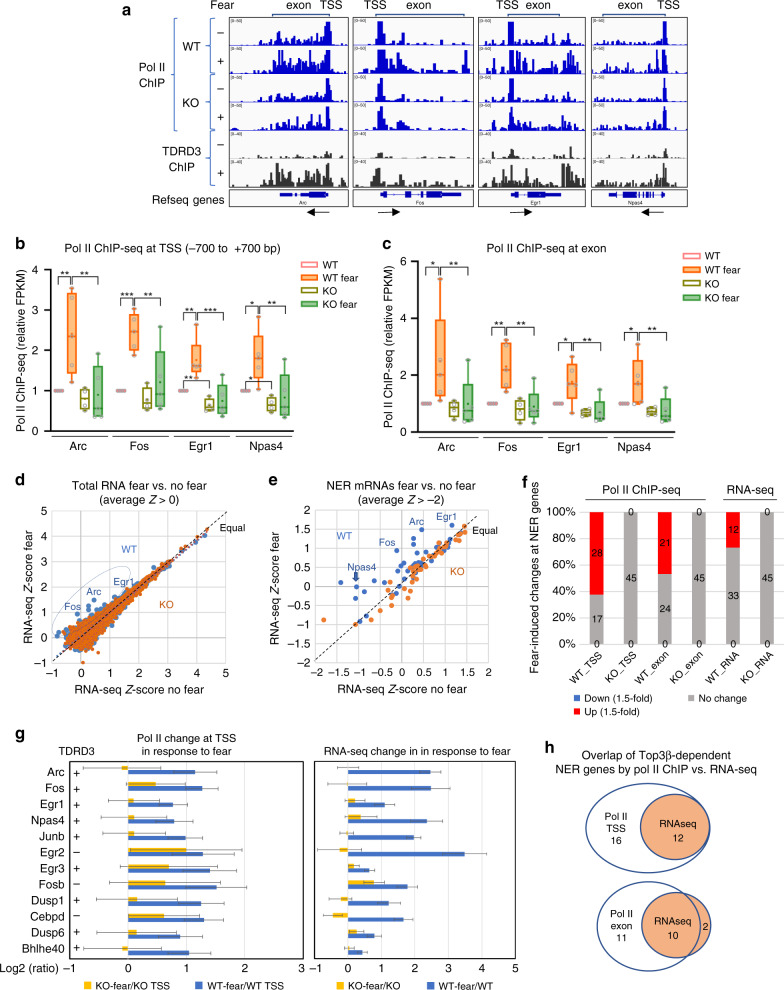
Top3β-KO mice have reduced NER gene transcription under fear conditioning by Pol II ChIP-seq and RNA-seq. **a**–**c** Top3β-KO have reduced Pol II signals in 4 NER genes by bedgraph (**a**; Supplementary Fig. 4a) and Box and Whisker plots (**b**, **c**) analysis of ChIP-seq data at TSS and exons. Untreatment (−) or FC treatment (+) are marked. TDRD3 ChIP signals are included, which are induced by FC similarly as Pol II signals in WT but not Top3β-KO (a; Supplementary Fig. 4b). The data of (**b**, **c**) are from four pairs of untreated, and five pairs of FC-treated mice. Pol II signals relative to the untreated WT are shown. The Whiskers mark the highest and lowest of data points; the Box marks the first and third quantile; and the center line marks the median. *P*-values (Supplementary Table 1) < 0.05, 0.01, and 0.001 (pair-wise comparison based on 2-way ANOVA analyses of log-transformed data) are marked as: *, **, ***. **d**, **e** Representative scatter plots of all genes (**d**), or NER genes (**e**), show that RNA-seq signals for some NER genes are induced by FC in WT (blue) but not Top3β-KO (orange). The data points from the 4 NER mRNAs of WT are above the equal line, indicating that they are induced by FC (2 mice/group). **f** A graph shows the percentage and number of NER genes that were upregulated, no change, or downregulated in response to FC for WT and Top3β-KO. The data are based on Pol II ChIP and RNA-seq signals. The threshold is 1.5-fold, and *p* < 0.05. **g** Graphs show that 12 NER genes that exhibit significant induction (*p* < 0.05) by FC in WT but not Top3β-KO based on Pol II ChIP signals at TSS (left) and RNA-seq signals (right). The TDRD3-bound and unbound genes are indicated by “+” and “−”, respectively. The log2_ratios of Pol II signals between fear and no-fear treated WT or Top3β-KO are shown. Error bars represent standard errors. 2-way ANOVA test was performed. (**h**) Venn diagrams show strong overlap between FC-induced NER genes determined by Pol II ChIP-seq and those by RNA-seq, as revealed in (**f**). Source data are provided as a Source Data file.

Similar to previous findings^16^, we detected Pol II peaks at transcription start-sites (TSS) and exons of all four NER genes in WT mouse brains; and these peaks were increased when mice were treated with FC (Supplementary Fig. 4a; Fig. 4a). Quantification of data from 4 pairs of untreated and FC-treated WT mice revealed that these increases are significant (*p* < 0.05) (Fig. 4b, c), consistent with robust NADT of these genes. Notably, the same analysis failed to show significant increase in Top3β-KO mice with FC (Supplementary Fig. 4a; Fig. 4a–c), indicating defective NADT. Moreover, with FC, the Pol II signals at both exons and TSS in the Top3β-KO mice are significantly lower than those of WT mice for all four genes; which is contrast to the no treatment group where Pol II signals in the mutant are significantly reduced for only 2 genes (*Egr1* and *Npas4*) at TSS (Fig. 4b, c). These data suggest that Top3β is critical for NADT, but its importance is reduced for basal transcription.

We have observed between-animal variability in Pol II ChIP signals in response to FC treatment, as well as in the fear behaviors (Fig. 1f–h). One possible explanation for the variability is that the hormonal levels of the animals are different.

To determine which step is defective during transcription in Top3β-KO mice, we performed ChIP-seq with phospho-specific antibodies against initiation or elongation forms of Pol II, which are marked by phosphorylation at Ser-5 (p-Ser5) or Ser-2 (p-Ser2), respectively, within the CTD repeats of the polymerase. We observed significant FC-induced signals of Pol II-Ser2 for *Arc* and *Npas4* (Supplementary Fig. 5a, b), but not p-Ser5 (Supplementary Fig. 5c, d), in WT mice, suggesting that elongation could be the step stimulated by neuronal activity. We also observed significant reduction of p-Ser2, but not p-Ser5, signals for the same two genes, in Top3β-KO mice without or with FC (Supplementary Fig. 5b, c). This suggests that Top3β can enhance the elongation step during basal and NADT.

Scatter plots of RNA-seq data for all genes or NER genes revealed FC-induced RNA increase for all four NER genes in WT, but not Top3β-KO mice (Fig. 4d, e; their data points are above the equal line in WT but not Top3β-KO; 4g, right). RT-qPCR confirmed significant reduction of FC-induced RNA increase in Top3β-KO mice for *Arc, Egr1*, and *Npas4* (*p* < 0.05); and a strong trend of reduction for *c-fos* (*p* = 0.055) (Supplementary Fig. 6a-d). These data are largely consistent with Pol II ChIP data, indicating that Top3β is required for NADT.

### TDRD3 and Pol II bind NER genes similarly in response to FC

To determine if the Top3β-TDRD3 complex directly binds the above NER genes, we performed ChIP-seq and detected a peak of TDRD3 at TSS of these genes in WT mice (Fig. 4a). Notably, TDRD3 showed increased signals at both TSS and exons in WT mice treated with FC, which resemble the increased Pol II signals, suggesting that in response to FC, the Top3β-TDRD3 complex may bind TSS, and then move along with Pol II on DNA to stimulate both transcriptional initiation and elongation. In comparison, TDRD3 ChIP signals from brains of Top3β-KO mice were lower than those of WT mice under FC (Supplementary Fig. 4b), which is expected because TDRD3 stability is lower in the absence of Top3β^1^, indicating that the TDRD3 signals observed are specific.

We noted TDRD3 binding to some but not all NAR enhancers identified in cultured neurons^16^(Supplementary Fig. 4b), including those in *c-fos* and *Npas4* genes (Supplementary Fig. 7a, b). This binding was increased by FC at several enhancers, and also co-localized with Pol II binding at some of them (*Fos*-e2, e5; *Npas4*-e2), suggesting that Top3β-TDRD3 may directly bind these enhancers to work with Pol II during NADT. In support of this, Pol II signals showed a trend of increase at two of these enhancers (*p* = 0.07–0.08) in response to FC (Supplementary Fig. 7a-c), which are consistent with the earlier findings^16^; although the Pol II difference between WT and Top3β-KO mice was not significant.

### Top3β is needed for transcription of many NER genes

We expanded our analysis to 45 NER genes in FC response, and found that 28 (62%) and 21 (47%) showed a significant increase of Pol II binding at their TSS and exons (*p* < 0.05; >=1.5 fold), respectively, in WT mice; whereas none showed significant increase in Top3β-KO mice (Fig. 4f, Supplementary Fig. 8a, b; Supplementary Table 1). The average of Pol II signals for 45 NER genes was increased by 1.9- and 1.6-fold at TSS and exons, respectively, in WT mice; but 1.1-fold and no significant change in Top3β-KO mice (Supplementary Fig. 9a). Similarly, the average of RNA-seq signals for the 45 NER genes was increased by 2.0-fold in WT, but unchanged in Top3β-KO mice (Supplementary Fig. 9b). 12 of the 45 NER genes exhibited significant increase of RNA-seq signals in WT mice, whereas none did in Top3β-KO mice (Fig. 4f, g). These 12 Top3β-dependent NER genes identified by RNA-seq overlapped with those by Pol II ChIP-seq (Fig. 4g, h), providing additional evidence that they depend on Top3β for NADT. Notably, majority of NER genes (about 80%) that depend on Top3β for FC-induced Pol II binding or RNA induction exhibited TDRD3 binding under FC (Fig. 4g; Supplementary Fig. 8a, b), suggesting that these genes may be directly regulated by Top3β-TDRD3 complex during NADT. Consistent with this, 21 of 45 NER genes (47%) displayed at least 1.5-fold lower Pol II signals in exons in Top3β-KO mice compared to WT mice under FC, in contrast to the no-treatment group in which only one gene showed significantly lower signals (*Jun*) (Supplementary Fig. 9c). The latter findings support the notion that Top3β is more important for activated than basal transcription. As a comparison, only 4 of 30 (13%) actin-related house-keeping genes showed significant reduction in Pol II signals in Top3β-KO mice under FC (Supplementary Fig. 9d).

### Top3β is required for global NADT

**Fig. 5:**
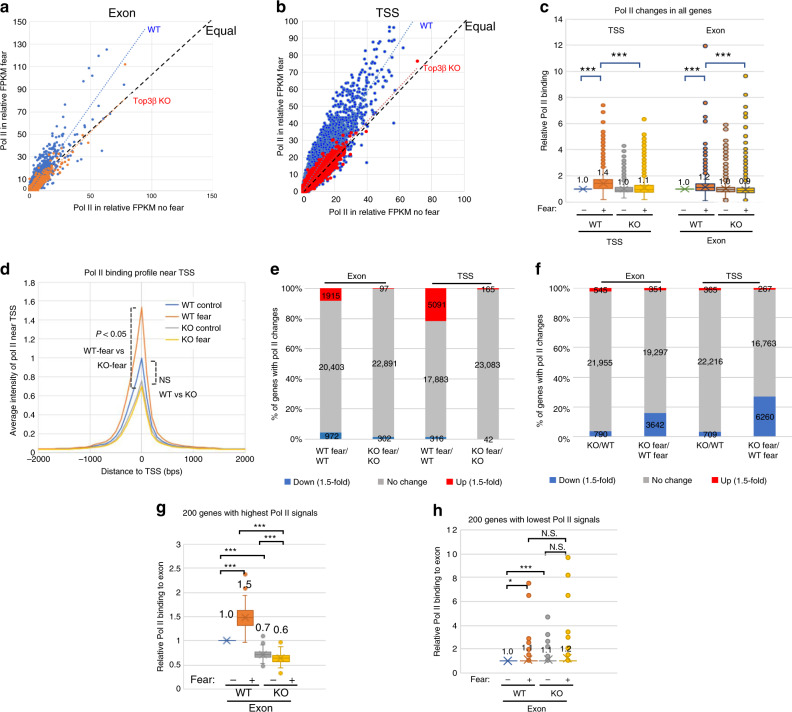
Top3β is required for global NADT. **a**, **b** Representative scatter plots show that a global increase Pol II signals (in FPKM) at exons (**a**) and TSS (**b**) in FC-treated WT mice, but not in Top3β-KO mice. This is evidenced by that WT mice have Pol II trend line and more data points above the equal line, whereas Top3β-KO mice have similar number of data points above or below equal line. **c** A graph illustrates an FC-induced increase of relative Pol II ChIP signals at TSS and exons of all genes in WT but Top3β-KO mice. The data used geometric means derived from four pairs of untreated mice, and five pairs of FC-treated mice. They are normalized using data from untreated WT mice as the standard (set as “1”). **d** A graph shows that average intensity of Pol II signals peak at TSS of all genes. Note that the Pol II peak is increased by FC treatment in WT but not Top3β-KO mice. **e** A graph shows the number of genes that have upregulated, no-change, or downregulated Pol II signals by FC at exons and TSS of WT or Top3β-KO mice. The threshold is 1.5-fold (*p* < 0.05). **f** A graph shows the number of genes that have upregulated, no-change, or downregulated Pol II signals between Top3β-KO and WT mice, without or with FC. (**g**) same as (**c**), except that only the top 200 genes with the strongest Pol II signals at all exons in untreated WT mice are shown. (**h**) Same as (**c**), except that only bottom 200 genes with lowest Pol II signals at all exons in untreated WT mice are shown. The relative average intensity is indicated in the graphs. No significant difference (*p* > 0.05) is marked as “N.S.”. *P*-values < 0.05, 0.01, and 0.001 are marked as: *, **, ***, respectively. Two-tail Student's *t* test was used in statistical analysis. Source data are provided as a Source Data file, which describes detailed calculations including *p*-values.

Detailed analysis of our Pol II ChIP-seq data showed that Top3β is critical for NADT of not only NER genes, but also thousands of other genes under FC. First, scatter plots revealed that in WT mice, a large fraction of genes showed higher Pol II signals at both exons and TSS in response to FC (Fig. 5a, b; more data points are above the equal line), indicating that FC-induced global NADT. In contrast, in Top3β-KO mice, the number of genes with increased and decreased Pol II signals are about equal, indicating defective global NADT. Second, the averages of Pol II signals at both TSS and exons of all genes were significantly increased in response to FC in WT mice, but this increase became smaller or absent in Top3β-KO mice (Fig. 5c). Third, the averages of Pol II signals in Top3β-KO mice were lower than those in the WT mice with FC, but they were identical without FC (Fig. 5c). Fourth, the average intensity of the Pol II peak near TSS is higher in WT than Top3β-KO mice under FC, but not significantly different without FC (Fig. 5d). Fifth, the number (and percentage) of genes that are induced by FC by >1.5 fold are about 20–30 times more in WT mice than Top3β-KO mice (Fig. 5e). Sixth, the number (and percentage) of the genes showing decreased Pol II signals (1.5-fold, *p* < 0.05) in the Top3β-KO mice with FC are about 5–9 times more than those without FC (Fig. 5f). These data further support the notion that Top3β is critical for global NADT, but less so under basal transcription.

We noticed that the number of genes with higher Pol II signals after FC are much more in WT than Top3β-KO mice (Fig. 5a, b), indicating that Top3β is more needed for highly expressed genes. To verify this, we calculated the averages for the top and bottom 200 genes with the highest and lowest Pol II signals, respectively; and observed significant Pol II reductions only in the former but not the latter group (Fig. 5g, h). The data support the notion that highly expressed genes produce more topological stress, and thus have stronger dependence on Top3β.

### TDRD3 preferentially binds TSS of genes activated by FC

**Fig. 6:**
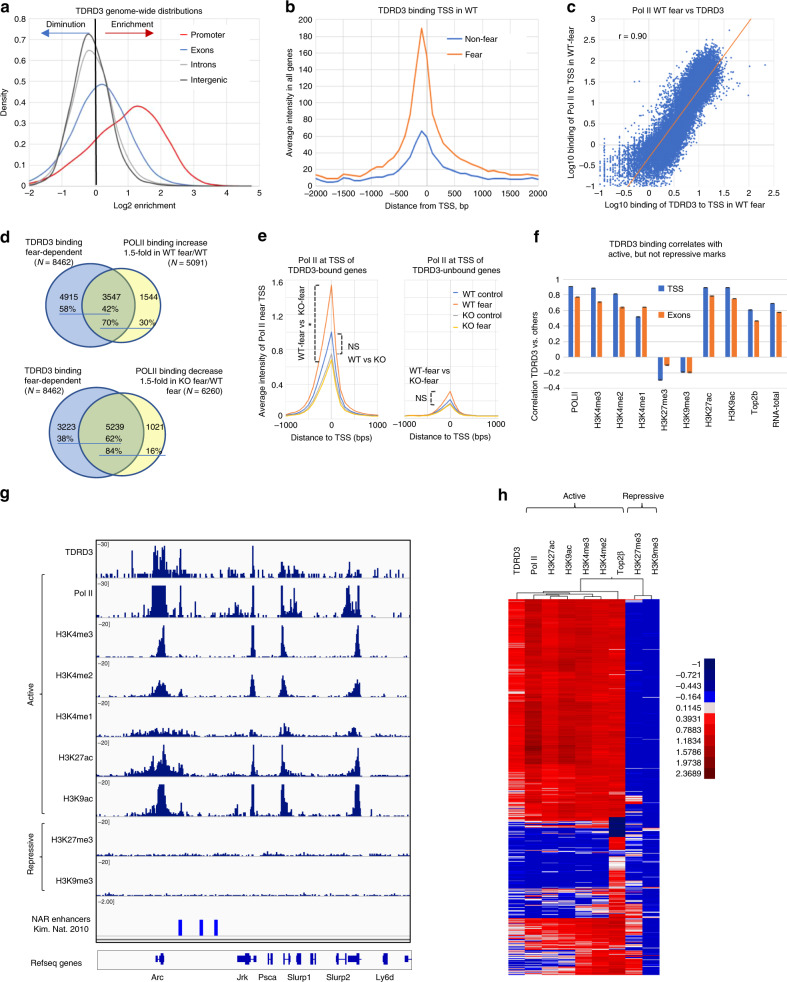
TDRD3 preferentially binds transcriptionally active genes depending on Top3β in NADT. **a** A graph shows genome-wide distribution of TDRD3 ChIP-seq signals in WT mice treated with FC. Notably, these signals are enriched in promoters and exons. **b** A graph shows that average intensity of TDRD3 ChIP signals peak at TSS, and this peak is increased by FC treatment. **c** A scatter plot shows strong correlation (*r* = 0.90) between log-transformed Pol II and TDRD3 signals at TSS in FC-treated WT mice. **d** Venn diagrams show overlap between the genes bound by TDRD3 under FC, and either genes with FC-induced Pol II increase in WT mice (top), or genes that have reduced Pol II signals in Top3β-KO mice under FC in comparison to WT mice (bottom). (**e**) Same as (**b**) except that Pol II signals at TSS of TDRD3-bound (left) and -unbound (right) genes from both WT and Top3β-KO mice were used in the analysis. The difference between WT and Top3β-KO is significant only for TDRD3-bound but not -unbound genes under FC treatment (*; *p* < 0.05). Note that the Pol II signals of TDRD3-unbound genes are lower than those of TDRD3-bound genes. 2-way ANOVA analysis of Log-transformed data was performed. **f** A graph shows average correlation coefficients between TDRD3 signals at TSS or exons of FC-treated WT mice and those of active (H3K4me1-3, H3K9Ac, H3K27Ac) or repressive (H3K27me3 and H3K9me3) chromatin marks, Pol II, RNA-seq. The published Top2β ChIP data from ES cells^14^ was included in analysis. **g** A representative bedgraph shows colocalization of TDRD3 signals in FC-treated WT mice colocalize with those of Pol II, and several active but not repressive chromatin marks, at TSS, exons, and enhancers within intergenic regions. **h** Clustering analysis shows that genes with strong TDRD3 signals at TSS are preferentially clustered with transcriptionally active genes, which have higher signals of Pol II, active but not repressive chromatin marks, and Top2β. *N* = 2 for the RNA-seq. *N* = 2 for ChIP-seq of TDRD3 and the active and repressive chromatin marks of the ChIP-seq. Source data are provided as a Source Data file.

We found that TDRD3 signals are enriched strongly at promoters, weakly in exons, but not in introns or intergenic regions (Fig. 6a). The average intensity of TDRD3 signals for all genes peaked immediately upstream of TSS, and became stronger when mice were under FC (Supplementary Fig. 10a; Fig. 6b). This pattern mimics that of Pol II (Fig. 5d), suggesting that in response to neuronal activation, Top3β-TDRD3 may be co-recruited with Pol II to the same TSS to activate their transcription. This notion is supported by the following evidence. First, TDRD3 and Pol II signals are co-localized at the same TSS of many genes (Fig. 4a; Supplementary Figs 11c, d and 12c). Second, TDRD3 signals at TSS of all genes have very strong correlation with Pol II signals under FC (correlation coefficient *r* = 0.9) (Fig. 6c), and a modest correlation with FC-induced increase of Pol II signals (r = 0.5) (Supplementary Fig. 10b, left). Third, Venn analysis shows that a large number of genes (42%) with FC-induced TDRD3 binding also have FC-induced Pol II increase; and majority (70%) of genes with FC-induced Pol II signals also have TDRD3 binding (Fig. 6d, top). Fourth, the TDRD3-bound genes have significantly higher average of intensity of Pol II signals at TSS than TDRD3-unbound genes in FC-treated WT mice (Supplementary Fig. 10c, left).

### TDRD3-bound genes show stronger dependence on Top3β for NADT

Our Venn analysis revealed that majority of genes (62%) with FC-induced TDRD3 binding also have reduced Pol II signals in FC-treated Top3β-KO mice; and even more genes (84%) with reduced Pol II signals in FC-treated Top3β-KO mice have TDRD3 binding (Fig. 6d, bottom; Supplementary Table 2). These strong overlaps suggest that Top3β-TDRD3 likely directly binds and promotes NADT of its target genes. In support of this, the average Pol II intensity at TSS for TDRD3-bound genes is significantly lower in Top3β-KO than WT mice under FC, in contrast to that for TDRD3-unbound genes that shows no significant difference (Fig. 6e). Moreover, TDRD3 signals at TSS exhibited a negative correlation with the ratio between Pol II signals of Top3β-KO mice vs. WT mice under FC (*r* = −0.67) (Supplementary Fig. 10d, left), suggesting that Top3β-TDRD3 preferentially binds genes that show reduced NADT without Top3β. Furthermore, the correlation and slope between TDRD3 binding at TSS vs. FC-induced increase of Pol II signals was smaller in Top3β-KO than WT mice (Supplementary Fig. 10b, right vs. left), providing additional evidence that Top3β-TDRD3-bound genes are more impaired in NADT in the absence than presence of Top3β.

We noted that without FC, the average intensity of Pol II peak at TSS was not significantly changed in Top3β-KO mice for either TDRD3-bound or -unbound genes, in contrast to the FC-treated group, which showed significant Pol II reduction for TDRD3-bound genes in Top3β-KO mice (Supplementary Fig. 10c, right vs. left). These data suggest that Top3β-TDRD3-bound genes have stronger dependence on Top3β for activated than basal transcription, which resemble the results from all genes (Fig. 5d). This conclusion is also supported by findings that the negative correlation and slope between TDRD3 signals and the ratio of Pol II signals of Top3β-KO vs. WT mice are smaller without FC than with the treatment (Supplementary Fig. 10d, right vs. left).

### TDRD3 preferentially binds active genes and chromatin marks

We found that TDRD3-bound genes have higher average Pol II signals than TDRD3-unbound genes, with or without FC (Fig. 6e**;** Supplementary Fig. 10b, c). This is confirmed by a very strong correlation between TDRD3 and Pol II signals at both TSS (*r* = 0.9) and exons (*r* = 0.8) in FC-treated WT mice (Fig. 6f); and also by strong correlations between TDRD3 signals at either TSS or exons vs. RNA-seq signals (*r* = 0.7, or 0.6, respectively) (Fig. 6f). These data together with those in Fig. 4 indicate the Top3β-TDRD3 complex has a strong preference to bind and regulate highly transcribed genes.

Our findings above that TDRD3 binding is enriched at promoters and strongly correlates with Pol II and RNA-seq signals are similar to the profiles of Top2β^14^. This prompted us to investigate if TDRD3 resembles Top2β in preferentially binding to active marks^14^. We found that TDRD3 signals colocalize (Fig. 6g) and correlate (Fig. 6f) with four active marks (H3K4me2, H3K4me3, H3K9ac, and H3K27Ac), very strongly at TSS (*r* > 0.8), and strongly in exons (*r* = 0.6–0.8); but not with two repressive marks (H3K9me3 and H3K27me3)(r < 0). TDRD3 also has weaker but still positive correlation with an enhancer mark, H3K4me1 (Fig. 6f). Moreover, TDRD3 signals exhibit modest correlation with the published Top2β signals^14^ at TSS; and TDRD3-bound genes clustered mainly with genes with active marks, including those with Top2β (Fig. 6h). Thus, Top3β−TDRD3 resembles Top2β in preferentially binding active genes.

### Top3β regulates dementia and schizophrenia-related genes

**Fig. 7:**
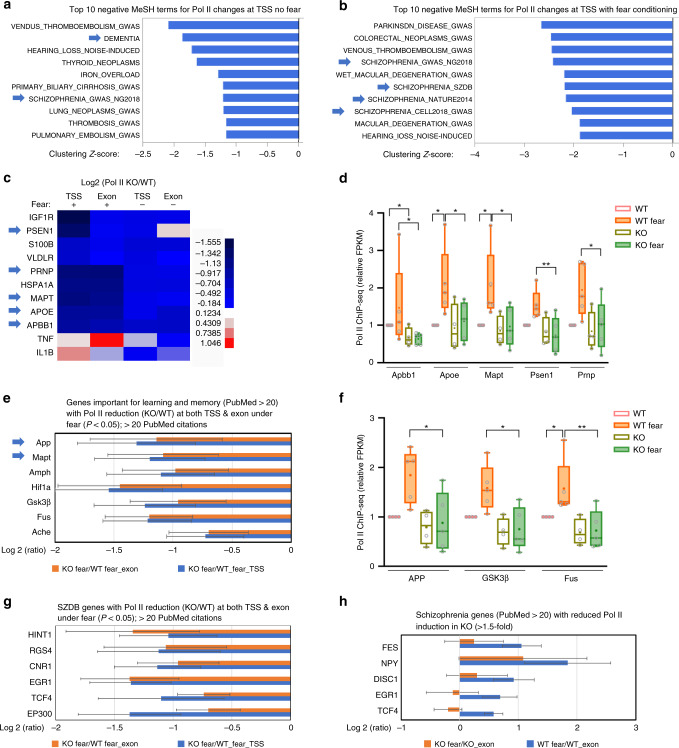
Top3β-KO mice have reduced Pol II signals in genes important for dementia, schizophrenia, and learning and memory. **a**, **b** A list of top10 negative MeSH terms (gene sets) identified using log2 fold-change between Pol II signals at TSS of Top3β-KO vs. those of WT mice, without (**a**) or with FC (**b**) treatment. Arrows indicate MeSH terms related to mental disorders. **c** A heatmap shows that dementia-related gene sets display overall reduction of Pol II signals at both TSS and exons, with or without FC. Genes related for AD are marked by arrows. **d** A Box-Whisker plot shows reduced Pol II signals at 5 dementia-related genes in Top3β-KO mice. The Whiskers mark the highest and lowest of data points; the Box marks the first and third quantile; and the center line marks the median. The data are from four pairs of untreated mice, and five pairs of FC-treated mice. Pol II signals (FPKM) relative to the untreated WT mice are shown. No-treatment control or FC treatment are marked. *P*-values <0.05, 0.01, and 0.001 are marked as: *, **, ***, respectively (2-way ANOVA test of log-transformed data). (**e**) A graph shows 7 commonly studied learning and memory-related genes (Pubmed citation ≥ 20) that show at least 1.5-fold reduction of Pol II signals in Top3β-KO mice under FC. The log2_ratios of Pol II signals between Top3β-KO vs. WT mice under FC are shown. Error bars represent standard errors (ANOVA). (**f**) same as (**d**), except 3 learning and memory-related gens were analyzed. (**g**) Same as (**e**) except that 6 commonly studied schizophrenia-related genes (Pubmed citation>20) from SZDB database were analyzed. **h** A graph shows reduced induction of Pol II signals of commonly studied schizophrenia genes by FC treatment in Top3β-KO mice. Source data are provided as a Source Data file, which describes detailed calculations including *p*-values.

We performed MeSH analysis^39^ to determine if genes with altered Pol II signals in Top3β-KO mice are associated with any gene sets important for psychiatric disorders. We identified dementia and schizophrenia as Top10-ranked negative MeSH terms among 1661 total terms (Fig. 7a, b**;** Supplementary Fig. 11a,b), suggesting that genes related to these two diseases exhibit strong patterns of reduced transcription in Top3β-KO mice.

Examination of the dementia gene set revealed that 10 and 9 genes showed reduction in Top3β-KO mice without and with FC, respectively (Fig. 7c). These reductions were significant with FC for 5 genes critical to Alzheimer’s disease (AD): *Apbb*1, *ApoE*, *Mapt* (Tau), *Psen1*, and *Prnp* (Fig. 7d). Because impaired learning and memory is a hallmark of dementia and has been observed in individuals and mice carrying Top3β deletion, we analyzed Pol II changes for 169 genes involved in this process. We found that 7 (4.1%) and 31 (18.3%) showed at least 1.5-fold reduction without and with FC, respectively, in Top3β-KO mice (Supplementary Table 3). Among them are 7 commonly studied learning and memory genes (PubMed citations>20) that have at least 1.5-fold decrease of Pol II signals at both TSS and exons in Top3β-KO mice under FC (Fig. 7e), including those important for AD (*App*, *Mapt, Gsk3b*) and dementia (*Fus*) (Fig. 7f). Bedgraph analysis confirmed the decrease of Pol II at TSS of these genes under FC in Top3β-KO mice (Supplementary Fig. 11c, d). Moreover, induced TDRD3 binding at TSS of these genes were also observed under FC. These data suggest that Top3β-TDRD3 binds and promotes transcription of multiple genes important for dementia and learning and memory.

Examination of one gene set from the schizophrenia database SZDB ([http://www.szdb.org]; genes with scores > =2) revealed that among 279 SZDB genes, 5 (1.8%) and 60 (21.5%) showed reduction (>1.5 fold; *p* < 0.05) in Top3β-KO mice without and with FC, respectively (Supplementary Fig. 12a; Supplementary Table 4). 34 (12%) SZDB genes showed at least 1.5-fold induction (*p* < 0.05) in WT mice in response to FC, whereas only one gene responded to FC in Top3β-KO mice (Supplementary Fig. 12b; Supplementary Table 4). We further identified several schizophrenia-related genes (>20 PubMed citations) that have either significantly reduced Pol II signals (*p* < 0.05) at both TSS and exons under FC (Fig. 7g), or decreased induction of Pol II signals by FC, in Top3β mutant (Fig. 7h); and confirmed the results using bedgraph analysis (Supplementary Fig. 12c).

One schizophrenia-risk gene with reduced Pol II signals in Top3β-KO mice is p300, an acetyltransferase for H3K27Ac. We did not observe drastic reduction of H3K27Ac for most of NER genes in Top3β-KO mice (Supplementary Fig. 13a–d), arguing that p300-mediated H3K27 acetylation is not the major mechanism by which Top3β mutation causes the disease.

Because Top3β-KO mice have increased anxiety behaviors (Fig. 1), we analyzed 235 anxiety disorder-related genes. A fraction of these genes showed either reduction of Pol II signals, or decreased induction of Pol II, in Top3β-KO mice under FC (Supplementary Fig. 14a, b; Supplementary Table 5). Among them are 7 well-characterized anxiety-related genes (PubMed citation > 100) (Supplementary Fig. 14c-e). These data suggest that Top3β is essential for NADT of a large number of genes related to psychiatric disorders and dementia.

### Top3β regulates synapse and neurogenesis-related genes

**Fig. 8:**
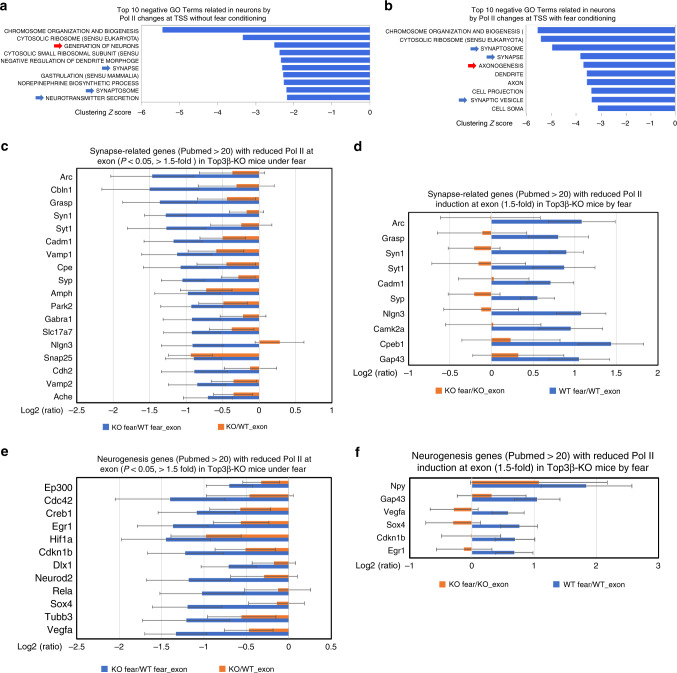
Top3β-KO mice have reduced Pol II signals in genes important for synapse and neurogenesis. **a, b** Lists of top10 negative GO terms (gene sets) identified using Log2 fold changes between Pol II signals at TSS of Top3β-KO vs. those of WT mice, without (**a**) or with FC treatment (**b**). Red and blue arrows indicate GO terms related to neurogenesis and synapse, respectively. **c** A graph shows 18 well-studied synapse-related genes (Pubmed citation ≥ 20) that have stronger reduction Pol II signals at exons in Top3β-KO mice treated with FC (*P* < 0.01) than that without the treatment. Log2_ratios of signals between Top3β-KO vs. the WT mice are shown. 2-way ANOVA test was performed for this and other graphs. **d** A graph shows 10 well-studied synapse-related genes (Pubmed ctation ≥ 20) that have at least 1.5-fold induction in WT mice by FC (*p* < 0.05), but not in Top3β-KO. Log2_ratios of signals between FC-treated and untreated mice are shown. **e**, **f** Same as (**c**, **d**), exc**e**pt that the data from well-studied neurogenesis-related genes are shown. Error bars represent standard errors (ANOVA). The data are from four pairs of untreated mice, and five pairs of FC-treated mice. Source data are provided as a Source Data file, which describes detailed calculations including *p*-values.

We performed Gene Ontology (GO) analysis and found that several gene sets related to synaptic function and neurogenesis, which are defective in Top3β-KO mice, are ranked in the top 10 negative neural terms using Pol II signals at TSS without or with FC (Fig. 8a, b). The data suggest that synapse and neurogenesis-related genes may exhibit overall reduced transcription in Top3β-KO mice. Consistent with this notion, among commonly studied synapse-related genes (Pubmed citation>20, *N* = 70), 18 had reduced Pol II signals (*p* < 0.05) (Fig. 8c), and 10 exhibited reduced induction of Pol II signals (<1.5-fold) by FC in Top3β-KO mice (Fig. 8d; Supplementary Table 6). Four of the most commonly studied synapse-related genes (Pubmed > 100) not only had reduced Pol II signals under FC in Top3β-KO mice (Supplementary Fig. 15a), but also were bound by TDRD3 (Supplementary Fig. 15b), suggesting that they could be directly regulated by Top3β-TDRD3 complex. Similarly, among neurogenesis-related genes (Pubmed > 20, *N* = 69), 12 had reduced Pol II signals (Fig. 8e), and 6 exhibited reduced induction of Pol II signals by FC (Fig. 8f; Supplementary Table 7), in Top3β-KO mice. At least two of these genes had TDRD3 binding upon FC (Supplementary Fig. 16a, b). Together, these data suggest that Top3β-TDRD3 directly binds and stimulates transcription of multiple genes important for synapse and neurogenesis.

## Discussion

Top3β mutations have been linked to several psychiatric and cognitive disorders, but whether they are causal remains unclear. Here we show that Top3β-KO mice display several abnormal behaviors (Fig. 1) present in schizophrenia and/or autism-spectrum disorders^20,21,40–42^, suggesting that Top3β deletion could be causal for these disorders in its human carriers.

The abnormal behaviors displayed in Top3β-KO mice overlap but are not identical to those of known mouse models of schizophrenia and autism (Supplementary Table 8). For example, although Top3β interacts with FMRP, the phenotypes of Top3β-KO mice are not identical to those of the Fmr1 mice (an autism model). In the light-dark test, Top3β-KO mice spent more time in the dark than light boxes, whereas Fmr1 mice show no preference^43^. In the FC test, Top3β-KO mice displayed an increase of contextual fear memory, whereas Fmr1 mice showed a decrease^43^. Furthermore, the divergence of Top3β-KO mice in memory behaviors—enhanced fear memory in FC test but reduced spatial memory in Morris water maze and contextual discrimination tests—resemble an AD mouse model (3xTg-AD) at young age^44^. Overall, these data argue that Top3β-KO mouse is not a simple schizophrenia or autism model, but rather, shares features with several psychiatric and cognitive disorders. This fits well with human genetic data that Top3β mutation carriers have a spectrum of behavioral alterations related to multiple psychiatric disorders. It is also consistent with our Pol II ChIP-seq data that Top3β deletion disrupts NADT of many genes, which include those from several neurological disorders.

It is known that adult neurogenesis and synaptic functions in hippocampus are important for learning and memory^31,32,34,45^. We found that both processes are defective in Top3β-KO mice, which could explain the cognitive dysfunctions observed in humans and mice with Top3β deletion. Notably, the impaired synaptic plasticity phenotype in Top3β-KO mice resembles those observed in patients^46^ and a mouse model (DISC1)^47^ of schizophrenia, as well as in several autism mouse models^48,49^. Similarly, the decreased adult neurogenesis phenotype was also seen in the schizophrenia model^50^. We found that both LTP and LTD at hippocampal CA1 synapses were impaired in Top3β-KO mice, whereas PPF was not, suggesting a postsynaptic deficit in the Top3β-KO mice.

**Fig. 9:**
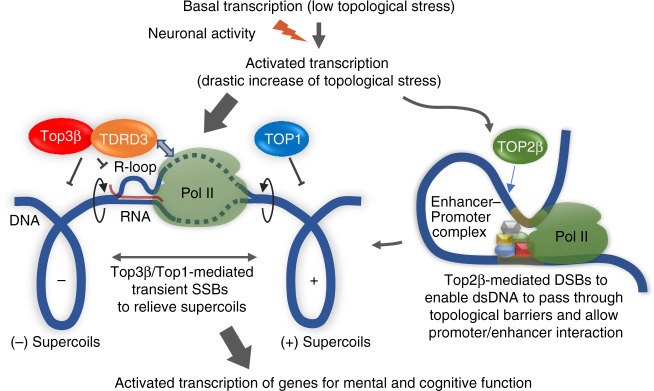
A model illustrates how Top3β-TDRD3 complex and Top2β works together to promote NADT. The model suggests that basal transcription produces low topological stress, so that the requirement for topoisomerases is low. Neuronal activity activates transcription of thousands of genes and enhancers, and thus induces a drastic increase of topological stress, including positive and negative supercoils, within a very short period. Top3β-TDRD3 complex is then recruited to these genes through Tudor domain-mediated interactions with arginine-methylated CTD of Pol II. Top3β can create transient SSBs to relax the negative supercoils generated during transcription and reduce R-loop formation (which inhibits transcription). Top1 can also generate transient SSBs to relax the positive supercoils. Transcription can then proceed normally. For some NER genes, there exist other topological barriers that prevent interactions between transcription machinery at enhancers and promoters. These genes require participation of Top2β to produce DSBs to overcome the topological barriers and enable assembly of activated transcription complex^13^. In the Top3β-mutant mice, global neuronal activity-activated transcription is impaired, due to failure to efficiently relieve topological stress. The affected genes include many that are important for neural development, mental health, and cognitive function. The defective transcription of these genes may contribute to the mental and cognitive disorders.

This study shows that Top3β plays a crucial role in NADT, which is essential for developmental and adult neuroplasticity, and has been linked to multiple mental disorders^19^. We propose a model that neuronal activity activates transcription of thousands of genes and enhancers^16^, producing a sharp increase of topological constraint in the genome within a very short period of time (Fig. 9). Top3β-TDRD3 complex may bind these genes and enhancers via TDRD3-mediated interactions with arginine-methylated CTD motifs of Pol II^51^ to relieve the topological constraint, leading to transcription of genes important for mental health and cognitive function. In support of this model, many genes linked to mental disorders, such as dementia, AD, schizophrenia, and anxiety disorders, show reduced Pol II signals in Top3β-KO mice. Moreover, a number of genes important for learning and memory, synapse function and neurogenesis also show reduction. The defective transcription of these genes may provide another explanation for the observed behavioral abnormalities in humans and mice carrying Top3β deletion.

It has been demonstrated that Top2β-mediated formation of DSBs is required for NADT of several NER genes^13^. We found that NADT of these NER genes also depend on Top3β (Fig. 4; Supplementary Fig. 17). The data suggest that for these genes, different types of topological constraint may have been produced, which require actions by both Type I (Top3β) and II (Top2β) topoisomerases to resolve (Fig. 9). Indeed, the two topoisomerases share several common features: enriched at promoters, preferentially binds active genes, and localizes in active chromatin marks. Moreover, the signals of these two topoisomerases at TSS showed positive correlation, and their bound genes showed co-clustering. Together, these data suggest that TDRD3-Top3β may work with Top2β to stimulate transcription of many genes in neurons.

## Methods

### Animals

Top3β-KO mouse is originally produced at Harvard University by targeted disruption of mouse Top3β on a C57BL/6J background^17^. Age-matched male mice were used in all experiments, whereas female mice were included in some behavior tests, as indicated below. 2–4 months old mice were used for this study. Target mice in social testing were ordered from Jackson Laboratories. All animal procedures were approved by the NIA animal care and use committee (ACUC) and followed the NIH animal guidelines.

### Mouse behavioral tests

*Elevated-plus maze test*: The apparatus is at 50 cm height and consists of two open arms and two closed arms radiating out at 90-degree angles. The closed arms are protected by 30 cm high walls, whereas the open arms have 2.5 cm high walls. Mice were placed in the center of the apparatus and allowed to explore for 5 min. ANY-Maze video system measures the number of entries and the time spent in each arm. (18 WT (13 males and 5 females) and 18 Top3β-KO (12 males and 6 females) were examined.

*Light-dark box test*: Equipment and test procedures were adapted from a previous protocol^52^. The light-dark box has a light compartment (26 cm long × 22.5 cm wide × 23.5 cm tall) with transparent walls illuminated by overhead fluorescent lights, a dark chamber (13 cm long × 22.5 cm wide × 23.5 cm tall) with black walls, and a small (5.5 × 5.5 cm) opening that connects different compartments. Mice were placed in the light box and allowed to explore the environment for 10 min. An overhead camera connected to ANY-Maze software recorded the number of transitions between compartments, a measure that is sensitive to anxiolytic drugs^52,53^. 11 WT (6 males and 5 females) and 11 Top3β-KO (5 males and 6 females) were examined.

*Three-chamber sociability test*: Equipment and procedures were based on a previous publication^54^. The sociability apparatus (59 cm long × 29 cm wide × 22 cm tall) constructed of clear Plexiglas was divided into 3 equal-sized chambers with small doorways (5 cm wide × 8.5 cm tall) connecting each side chamber to the central chamber. Target mice were placed in custom-built cylinders (6.5 cm diameter × 12 cm tall) with walls of 3mm-wide stainless-steel rods spaced 7.5 mm apart to permit visual and olfactory interaction between test and target mice while preventing aggressive interactions. Each test session began with a 10 min habituation of test mouse into the sociability apparatus. Test mice were briefly removed from apparatus, and then cylinders were placed in both side chambers, with one empty and one containing a 4–5-week-old male C57Bl/6 J wild-type target mouse. The test mouse was then returned to the center chamber to commence a 5-min sociability test. An overhead camera was used with ANY-Maze software to score the length of time in chambers by the test mouse. 12 WT and 12 Top3β-KO mice of all males are used in this assay.

*Reciprocal social interaction*: Social interaction test was performed in a 25 × 25 × 20 cm opaque Plexiglas chamber under dim white lighting. After 30 min of habituation alone in the chamber, a 4–5 weeks old male juvenile mouse (C57Bl/6J albino, homozygous for Tyrc-2J gene; Jax stock 000058) was added in the chamber with the testing animal. The social interaction behavior was recorded for 10 min using a video camera. Frequency and duration of each social parameter detected in the test subject mouse were subsequently automatically analyzed using Noldus Ethovision XT (Noldus Information Technology, Leesburg, VA). Because mice were identified by the software by different colored fur, no paint or other markings were required. Parameters scored included following, nose-to-nose sniffing, nose-to-anogenital sniffing, sniffing of other body regions, and close following. 11 WT (6 males and 5 females) and 11 Top3β-KO (5 males and 6 females) mice were examined.

*Fear conditioning test*: Mice were tested for fear conditioned memory using a Video Fear Conditioning system (Med Associates). Conditioning chambers consist of plexiglass enclosure with stainless-steel grid rod flooring. In the conditioned training, mice were placed into the chamber for a total of 300 s. After 120 s of habituation, mice were presented with a 30 s tone that co-terminated with a 2 s, 0.5 mA electric foot shock. This was repeated for a total of three tone-shock pairings with an interval of 30 s between tone presentations. Twenty-four hours after conditioning, the mice were tested by a contextual experiment in the same chamber for 300 s without tone and electric foot shock stimuli. Three hours after the contextual test, cued testing was performed. Total time of the cued test was 600 s and the testing environment was altered by a smooth plastic floor covering the metal rods and a triangle shaped plastic insert above the inside of the chamber. After 300 s of resting time, animals were exposed to 3 tones of 30 s each with 30 s intervals. The Video Freeze software measured and recorded freezing behavior displayed by the mice during testing. 9 WT (4 males and 5 females) and 10 Top3β-KO (4 males and 6 females) mice were examined.

*Context discrimination test*: Mice were tested for ability to distinguish between a shock-paired environment (context A) and a neutral environment (context B). The specificity of freezing response to the correct environment is impaired by lesion of dorsal hippocampal tissue^25^, and enhanced following increased hippocampal neurogenesis^55^. Two distinct contexts were used (“A” and “B”) that differed in floor texture, wall shape, white vs infrared lighting, and odor. Housing rooms and transport method to and from the fear conditioning room were also different depending on which context was being used. Prior to training, mice were placed in context A for 10 min in the morning and context B in the afternoon for 10 min. 24 h later, training began and mice were put in context A for a total of 180 s. After 148 s of habituation (pre-shock period), mice were shocked by a 2 s, 0.5 mA electric foot shock. Context A chamber consists of straight 90-degree walls with stainless-steel grid rod flooring. White lighting in the box was on and 2 μl of mint extract (McCormick; Hunt Valley, MD) was put in the collection tray of the box. Four hours later, the mice were placed in context B for 180 s without shock. Context B consists of smooth plastic floor covering the metal rods and a triangle shaped plastic insert above the inside of the chamber. White lighting in the box was off and it was illuminated by near infrared lighting only. Additionally, 2 μl of orange extract (McCormick) was put on the ceiling of the chamber. Video Freeze software (MED-Associates; St Albans, Vermont) was used to measure and record freezing behavior displayed by the mice during testing. All freezing scoring was done using default settings of the software. The analysis was made on the pre-shock time between context A and Context B on training and test days. 11 WT (all males) and 12 Top3β-KO, mice of all males were examined.

*Morris water maze test*: This test followed a previous protocol^56^. The platform was 15 × 15 cm square and the water temperature was 23 °C. We ran one trial for each mouse before moving to the next trial, resulting in inter-trial intervals of approximately 15 min. The tub was filled with water to 0.5–1.0 cm above platform. Non-toxic white paint was used to render the platform invisible to mice in the water. Each animal performed 4 trials/day, starting from each of the four compass directions. During each day, mice were tested following a pseudorandom order that was maintained for all mice on that day. ANY-Maze software recorded and measured the time required to each mouse to reach the platform. Mice that did not reach the platform within 60 s, were gently guided towards it and left there for additional 15 s. 4 h following the last training, mice were placed in water without platform for the first probe trial. ANY-Maze recorded the time in each quadrant for 60 s. 7 WT and 7 Top3β-KO mice of all males were examined.

*Open field test*: The test mouse was placed on the square box (43 × 43 cm, height 30 cm) for open field test. Traveling distance was automatically measured by three 16-bean IR arrays for 30 min for each mouse. Time in center, number of center entries, and center distance were tracked. Recording and analysis were performed by Activity Monitor Software (Version 4.0, Med Associates, St. Albans, VT). 13 WT and 12 Top3β-KO mice of all males were used in the test.

*Rotarod test*: Five-lane rotarod (Med Associates, St. Albans, VT) was used to define motor function in Top3β-KO mice. Individual mouse was tested for 5 min per day for 3 days. After the mouse was placed on the rod for 10 s, the rod speed was accelerated from 3 to 30 rpm. We recorded the latency to the first fall and the total number of falls. 7 WT and 7 Top3β-KO mice of all males were tested.

F*orced swim test*: A cylinder beaker (10 cm in diameter and 25 cm height) with water (24~25 °C temperature) was used for the test. Individual mouse was placed in the water for 6 min. Their behaviors were recorded by a camera. The data were analyzed for time of immobility and mobility in the last 4 min of testing. Genotype information was blinded during the analysis. 7 WT and 7 Top3β-KO mice of all males were tested.

*Spontaneous alternation*: Y maze (each arm: 7.62 cm × 38.1 cm, height 12.7 cm) with non-visible gray color was used for spontaneous alternation test. Each mouse was placed in the center area to start the test. Their behaviors were recorded by performers. The arms that are entered and explored by the test mouse consecutively for 10 min are determined. 7 WT and 7 Top3β-KO mice of all males were tested.

*Prepulse inhibition*: Mice were contained in plexiglas cylinders that allowed moderate movement, which were housed within sound-attenuated cubicles. After a 5 min habituation period mice were presented with 40 ms pulses of white noise at increasing amplitudes, with 20–30 s intervals between each presentation. Noises were presented in order from low to high amplitude, with the full sequence repeated a total of five times. Startle response was measured by accelerometer that measured maximal force applied (Vmax) in the 50 ms period following noise presentation (SR-Lab; San Diego Instruments). The next day mice were tested for prepulse inhibition. Following habituation, mice were presented with 105 dB, 40 ms startle noise presentations in the presence or absence of 20 ms “prepulse” noise presentations that occurred 30 or 70 ms prior to the startle presentation and were 70 or 80 dB in amplitude. Each of the five possible trial types was presented in randomized order within a block, for six blocks. Data was analyzed and graphed as percent inhibition of Vmax response during no-prepulse trials. 6 WT and 5 Top3β-KO mice of all males were tested.

*Buried food test:* Procedures were based on a published protocol (Yang & Crawley 2009; Curr Prot Neurosci). Mice were habituated for 3 days with exposure to food overnight (50 mg pieces of cheese) by introducing three pieces of cheese per mouse per cage. Mice were fasted for 16–20 h prior to testing. One hour before testing, mice were transferred to new cages containing 5 cm deep corncob bedding, where they were individually housed. Mice were temporarily removed from the cage and a single 50 mg piece of cheese was buried at the bottom of one end of the cage. The mouse was returned to the cage and the time for the mouse to find and retrieve the food was measured. 6 WT and 5 Top3β-KO (all male) mice were tested.

### Electrophysiology

Transverse hippocampal slices (350 um) were prepared from mice brain, and maintained in Artificial cerebrospinal fluid (ACSF; in mM: 120 NaCl; 2.5 KCl; 1.25 NaH_2_PO_4_; 26 NaHCO_3_; 1.3, MgSO_4_; 2.5 CaCl_2_ and 10 glucose, pH 7.4). The osmolality was adjusted to 290 mmol/kg, using a Vapro Pressure Osmometer (Wescor). Slices were allowed to equilibrate for 1 h prior to recordings, and kept in a holding chamber for up to 6 h.

All recording solutions were without picrotoxin. Slices were recorded at 30–32 °C. Field excitatory postsynaptic potentials (fEPSPs) were recorded in CA1 stratum radiatum^57^. Stimuli (30 μs duration every 20 s) were delivered through a fine bipolar tungsten electrode to activate Schaffer collateral/commissural afferents, with stimulation intensity that evoked about 30–40% of the maximum of fEPSP. Long-term potentiation (LTP) was induced by high-frequency stimulation (HFS, 100 Hz, 1 s); NMDA-long term depression (LTD) was induced with 900 pulses at 1 Hz^58^. In PPD study, two stimulation conditions were used, either 30 or 50% of max subthreshold EPSP. The plots were normalized to the initial slope of the EPSPs; each data point represents the averaged values for 1 min (three consecutive sweeps with an interval of 20 s). Values are reported as mean ± SEM. Data were collected using a MultiClamp 700B amplifier (Molecular Devices); signals were filtered at 2 kHz, digitizedat 10 kHz and analyzed using pCLAMP 10 software (Molecular Device). Only males were used in the test, and their numbers are: 6 slices from 4 WT and 5 slices from 4 KO in neuronal strength test; 9 slices from 5 WT and 5 slices from 4 KO in LTP; 5 slices from 5 WT and 6 slices from 4 KO in LTD; and 6 slices from 4 WT and 6 slices from 6 KO mice in PPF.

### BrdU labeling of adult neural stem cells

One month old male mice were used for this experiment. Bromodeoxyuridine (BrdU, Sigma) was diluted in PBS to make a 10 mg/ml stock solution. After filtration, 0.2 ml of the BrdU stock solution (2 mg/day/mouse) was injected intraperitoneally for 3 days with a 24 h interval between each injection. At 6 h after the last injection, the  mice were deeply anesthetized and perfused transcardially as described in detail below in the section ‘Immunohistochemistry and imaging analysis’. The number of the BrdU positive cells were counted from 4 slices of the dentate gyrus of hippocampus region from each mouse (3 WT and 3 KO mice).

### Preparation of viral vectors to infect newborn neurons

Dividing progenitor cells in the dentate gyrus of the hippocamous were labeled using a replication-defective retroviral vector CAG-GFP, expressing enhanced green fluorescent protein (GFP) as previously described^59,60^. Plates of 90% confluent human embryonic kidney (HEK293T) cells were transfected with the vector CAG-GFP (7.5 μg), CMV-GagPol (5 μg) and CMV-VSVG (2.5 μg), using Lipofectamine 2000 (Invitrogen). Virus-containing supernatant was harvested 36 h later filtered and concentrated by ultracentrifugation. Virus titers were estimated to be ~ 1 × 108 i.u./ml by serial dilution into HEK293T cells.

### Stereotaxic surgery and viral vector injections

Intra-cranial injections of retroviral vector were performed as previously described^61^. We used male mice between 4–5 weeks old for this experiment. Experimental mice were housed individually before stereotaxic surgery and allowed to acclimatize for 3–5 days. Mice underwent surgery during which they were anesthetized with avertin (0.4 mg/g i.p.) and placed within a stereotactic frame (Stoelting stereotaxic Instruments, USA). 1 μl of retrovirus CAG-GFP at a rate of 0.1 μl/min was injected into the right dorsal and ventral dentate gyrus (DG). The coordinates used to target the dorsal and ventral DG relative to Bregma were as follows: Dorsal DG, anterior–posterior (AP) = − 2.10 mm; medial–lateral (ML) = 1.9 mm; dorso-ventral (DV) = − 2.10 mm, and ventral DG, AP = − 3.10 mm; ML = 2.8 mm; DV = − 3.10 mm. One month after viral vector infusion, the injected mice were anesthetized with isofluorane and perfused transcardially with 0.9% saline followed by 4% paraformaldehyde in 0.1 M PBS (phosphate-buffered saline). They were then subject to immunohistochemistry and imaging analysis. 5 WT and 5 Top3β-KO mice were used for retroviral injection. 1 or 2 slices/each mouse were analyzed (6 slices from the WT and 7 slices from the KO).

### Immunohistochemistry and imaging analysis

Immunohistochemistry was performed on hippocampus region of the brain. Briefly, following mice sedation by isoflurane, the brain was perfused by injecting 10 ml of PBS and 10 ml of 4% paraformaldehyde into protrusion of left ventricle with 10 ml syringe. After injection, brain was isolated and incubated overnight at 4 °C in 4% paraformaldehyde/PBS. The brain was transferred in 15% sucrose/PBS solution and incubated for 24 h at 4 °C. At the end of the incubation, the solution was substituted with a 30% sucrose/PBS solution and brain was incubated at 4 °C until they sank into the bottom of the tube. The brain was then frozen in OCT (optimal cutting temperature) compound, cryo-sectioned by 30 μm setting, and washed 3 times in PBS. Brain layers were heated at 80 °C in 10 mM sodium citrate buffer (pH 8.5) for 30 min for antigen retrieval, and placed at room temperature for 30 min. After washing 3 times with PBS, tissues were incubated at room temperature for 1 hr in blocking solution (10% normal goat serum and 0.3% Triton X-100 in PBS) in order to block non-specific binding of the antibodies. Tissues were then incubated overnight at 4 °C with primary antibodies in 10% normal goat serum/PBS. The antibodies are: rabbit anti-GFP conjugated with Alexa Fluor488 (1:200; Life technologies, A-21311), rat anti-BrdU (1:50, Abcam ab6326) and rabbit anti-doublecortin (1:200, Abcam, ab18723). After incubation, specimens were washed 3 times with PBS and incubated at room temperature for 1 hr with a secondary antibody: Fluorescein anti-rabbit IgG (Vector Labs, FI-1000); anti-rat Alexa Fluor 488; anti-rabbit CY3 (1:200, Jackson ImmunoResearch). Finally, brain layers were washed 3 times with PBS and incubated at room temperature for 10 min with DAPI (Life technologies, D1306) at 1:10,000 dilution. Images were acquired by confocal microscope, ZEISS LSM 800. The number of the BrdU positive cells were counted in 3-4 slices of the dentate gyrus of hippocampus region from each mouse. Tracing of dendrites and spines of GFP positive adult-born neurons was performed in coronal sections using Bitplane Imaris software (Oxford instruments).

### ChIP-seq

Mouse brain tissue was homogenized in 1 ml of 1% formaldehyde for 15 min. The sample was added of 50 μl of 2.5 M glycine (final concentration 125 mM), incubated at room temperature for 5 min, and centrifuged at 1000*g* for 5 min at 4 °C. The pellet was collected and washed with PBS. The pellet was then resuspended in 400 μl of RIPA buffer with protease inhibitor cocktail and 0.5 mM phenylmethylsulfonyl fluoride and centrifuge at 1000*g* for 10 min at 4 °C. The lysates were sonicated to shear the DNA to lengths between 200 and 400 bp, and were centrifuged at 1,000 x g for 10 min at 4 °C. In order to pre-clear the chromatin, 100 μl of Protein A beads were added to the sample and incubated for 60 min at room temperature. During preclearing step, Protein A beads were incubated with blocking solution (0.5% BSA in PBS) for 60 min at room temperature. 5 μl of antibody (RNA Pol II, Biolegend, 664906; Anti-RNA Pol II phospho S2 Ab, abcam, ab5095; Anti-RNA Pol II Phospho S5 Ab, Abcam, ab5131; Anti-Top3β Ab, Sigma, WH0008940M1; Anti-TDRD3 Ab, Rabbit polyclonial Ab was made against MBP-fused proteins, New England Biolabs; H3K4me1, Abcam, Ab8895; H3K4me2, Abcam, ab32356, H3K4me3, Abcam, ab8580; H3K9me3, Abcam, ab8898; H3K9AC, Abcam, ab4441; H3K27Ac, Abcam, ab4729; H3K27me3, Millipore/Sigma 07-449) and 20–30 μl of Protein A beads were added into the precleared lysate and incubated for 4 h at 4 °C and mixed on a rotator during all the incubation step. Each sample was washed one-time with 0.3 M NaCl/RIPA buffer, one-time with RIPA buffer, one-time with 0.25 M LiCl and twice with TE. The beads were incubated for 8 h at 65 °C with 100 μl TE, 3 μl of 10% SDS and 3μl of Protease K in a thermocycler, and the liquid was transferred into a new tube. 100 μl of TE was added to the beads, resuspended, and the supernatant was transferred again to the tube in order to reach a final volume of 200 μl. For DNA extraction, each sample was vortexed with 200 μl of Phenol/Chloroform and centrifuged. The supernatant was mixed with 20 μl of 3 M NaAc, 2 μl of GlycoBlue (Invitrogen, AM9515) and 3 volume of 100% Ethanol, and incubated for 30 min at −80 °C. After centrifuging for 25 min at 4 °C, the pellet was collected and washed with 70% Ethanol and centrifuged again for 5 min at 4 °C. Finally, the pellet was dried and resuspended with 50 μl of TE, and is used for sequencing analysis.

### RNA-seq and RT-qPCR

For RNA-seq, one brain hemisphere from two-month old male mouse was collected and homogenized in 2 ml of PBS containing RNase inhibitor (80 U) (Thermo Scientific, AM2696). Total RNA was extracted from 200 μl lysate using 1 ml of Trizol (Invitrogen, 15596026). The RNA was precipitated in 1.5-fold volume of 2-propanol together with 10% volume of 3 M Sodium Acetate. Genomic DNA was removed using DNase I (Sigma Aldrich, AMPT1-1KT). Ribosomal RNA was removed from total RNA using rRNA-depletion kit (New England biolab, E6310S). Then, the double-stranded cDNA was synthesized from the rRNA-depletion RNA using double-stranded cDNA synthesis kit (Invitrogen, 11917010). Double-stranded cDNA obtained was sonicated using Bioruptor for 4 times and 10 min each time with medium power settings (cycles of 15 s off and 15 s on). They were then used as templates for library preparation and deep sequencing.

For RT-qPCR, a hemisphere of 2 month old mouse brain was collected and homogenized in 2 ml of PBS with 80 U of RNase inhibitor (Thermo Scientific, AM2696). Total RNA was extracted from 200 μl lysate using 1 ml of Trizol (InvVitrogen, 15596026). RNA was precipitated in 1.5-fold volume of 2-propanol together with 10% volume of 3 M Sodium Acetate. cDNA was synthesized from 1 μg RNA using Taqman Reverse Transcription Reagents (Applied Biosystems, N8080234). After 10-fold dilution, the cDNA was used as a template to perform qPCR with SYBR Green PCR Master Mix (Applied Biosystems, 4309155). The PCR primer sequences are in Supplementary Fig. 18.

### Library preparation for deep sequencing

Forty microliters of sonicated DNA were mixed with 6 μl of 10X End repair buffer, 6 μl of 2.5 mM dNTPs, 6 μl of 10 mM ATP and 1.2 μl of End-Repair Enzyme mix (Invitrogen, IVGN2504), and incubated at room temperature for 45 min. The sample was purified by MinElute Reaction Cleanup Kit (Qiagen, 28204). 40 μl of DNA was added of 5 μl of NEB buffer 2, 1 μl of 10 mM dATP and 3 μl of Klenowexo- (NEB, M0212L) and incubated for 30 min in water bath at 37 °C. The DNA was then purified by MinElute Reaction Cleanup Kit (Qiagen, 28204) following the manufacturer instruction. 20 μl of eluted DNA was mixed with 3.1 μl of 10X T4 DNA ligase buffer, 5 μl of Index PE Adaptor oligo mix and 3 μl of T4 DNA ligase (400 units/μl), and incubated at room temperature for 30 min. Following the reaction, the sample was run in 2% agarose gel and the band positioned at 200–400 bp size representing the fragment of interest was excised for purifying the isolated DNA. The DNA was extracted from the agarose gel using MinElute Gel Extraction Kit (Qiagen, 28604) and following the instructions reported in the kit. Purified DNA was then amplified PCR using Solexa primers and enzyme mix, and the following conditions: 1 incubation cycle at 98 °C for 30 s, 15 incubation cycles at 98 °C for 10 s, 65 °C for 30 s and 72 °C for 30 s. The sample was loaded on agarose gel and the DNA fragment of interest was further extracted and purified by using MinElute Gel Extraction Kit (Qiagen, 28604). The DNA concentration was measured by Qubit 3.0 fluorometer (Life technologies, Q33216).

### Sequencing data analysis

Reads were mapped to mouse genome version mm10 using BOWTIE2 under “very sensitive” option and the maximum of 2 mismatches^62^. Reads with >3 matches in the genome were ignored, and the weight of other reads was set to 1/*n*, where *n* = the number of matches. No more than 5 reads with identical start and end coordinates were accepted in the calculation to avoid PCR-amplification bias. For ChIP-seq calculation, the intensity of protein binding to gene exons and TSS region (from −700 to +700 bp) was estimated in FPKM units, where total reads were counted in all transcripts, using a custom Perl program. To combine multiple replications of ChIP-seq data (5 replications under FC and 4 replications without treatment), we normalized values to wild-type mice without treatment in the same experiment. Statistical significance was evaluated using 2-way ANOVA test of log-transformed ChIP-intensity values, followed by pair-wise comparison of means using mean square error for each gene (or for each point in profiles at TSS). To select genes with FC-dependent binding of Pol II at the TSS region, we used False Discovery Rate (FDR) method^63^. Log2 ratios were calculated between Pol II signals of mutant and WT mice without or with FC treatment. They are then used as input to do (Parametric Analysis of Gene set Enrichment) PAGE analysis^64^ against Gene Ontology (ftp://ftp.geneontology.org/go/www/GO.database.shtml#dbtypes) and Disease MESH term gene set^65^ to find physiology and pathology effects of Pol II changes at these genes.

We also analyzed the ChIP-seq data use SICER 1.1 against mouse genome mm10 with windows of 100 bp-width and 600 bp-gap. The area with significant SICER scores (*p*-value < = 0.05, SICER score > = 200, and false discovery rate < = 0.05) are marked as “binding islands”. The input-subtracted scores for Top3β-KO mice were compared with those of the control, without or with FC treatment. The islands are further mapped to gene bodies to identify the possible effect of Pol II changes on specific genes.

For RNA-seq analysis, we first used Tophat 2.1.1 against UCSC mouse genome GRCm38/mm10 and sort /index the data files by Samtools 1.9. The sorted bam files are converted to bed format by Bamtools 2.5.1. The gene count data are obtained by htseq 0.11.2. The alignment data are assembled by cufflinks 2.2.1. The gene differential analyses have been done by both cuffdiff 2.2.1_patched version with fpkm normalization, and edgeR package with TMM normalization package. The significant gene lists are selected from both analyses by cutoff |log2_foldchange | > = 0.58, corrected q-value < = 0.05. The functional analysis was carried out by PAGE algorithm against Gene Ontology gene set. The principal component analysis and heatmap/cluster were done based on log2 transformed Z-scores of gene expression on the normalized FPKM values.

### MRI analysis

The WT/heterozygous and Top3β-KO mice underwent MRI scanning using a 7 T/30 cm preclinical Bruker Biospec scanner (Bruker Biospin, Ettlingen, Germany); *n* = 8 per group. Data were acquired when the mice were 2–3 months of age. A 72 mm diameter birdcage-design resonator was used for pulse transmission (Bruker), while signal reception was performed with a two-channel array coil designed for the mouse head (RAPID MRI International, Columbus, Ohio). Animals were anesthetized throughout using a snout mask. Induction was performed using isoflurane in oxygen in a 2.5% v/v mixture. After induction and during acquisition of MRI data, anesthesia was maintained with a 1–2% v/v mixture. Homeostasis to maintain a breathing rate of 40–60 breaths per minute was achieved by ongoing adjustment of the concentration of isoflurane. Heart rate and temperature were also monitored throughout the experiments using an SA Instruments MR-compatible model 1025 physiological monitoring system (SA Instruments, Stony Brook, NY), with a pneumatic respiration transducer and a fiber optic rectal temperature probe. This also permitted control of body temperature throughout the MRI scans; the temperature unit provided input to a feedback system regulating a flow of warm air (SA instruments blower) around the anesthetized animal. A set of 12 contiguous coronal slices, each of 1 mm thickness, was positioned to obtain imaging data covering the brain from the olfactory bulb to the brain stem. Imaging parameters included a 30 mm × 20 mm (left-right x anterior–posterior) field of view. Diffusion-weighted scans were acquired with voxels of size 117 × 156 × 1000 μm, with pixel resolution of 256 × 128. A spin-echo diffusion pulse sequence with fat suppression was implemented with parameters including repetition time TR = 4 s, echo time TE = 30 ms, gradient separation Δ = 14 ms, gradient duration δ = 7 ms, b value for diffusion-sensitization in the slice (H/F) direction of 200 s/mm^2^, with signal averaging over two acquisitions, resulting in a 17 min scan duration. Inversion-prepared spin-echo scans were also obtained. Imaging parameters included a voxel size of 313 × 156 × 1000 μm, with pixel resolution of 96 × 128 pixels. Sequence parameters of TR = 6 s, TE = 7.5 ms, and inversion time TI = 604 ms were used. Data were again averaged over two acquisitions, resulting in a scan duration of 25 min. After data acquisition, analysis of the images was performed using Bruker ParaVision 5.1 software. Regions of interest (ROI’s) defining the ventricles were drawn on all ventricle-containing slices obtained with the inversion-prepared sequence. After defining these ROIs, a calculation of the number of pixels contained in them was performed. This number was multiplied by the voxel volume, resulting in a measurement of ventricular volume. Total brain volume was determined similarly, using ROI’s that outlined the brain on each slice of the diffusion-weighted images. Brain volume was then calculated from the total number of pixels contained in these ROI’s through multiplication by voxel volume. 4 WT and 8 knockout male mice were used in the analysis.

### Statistical analysis

Statistical significance was assessed using Student’s *t* test or an ANOVA test with Tukey-Kramer post-hoc analysis. For RNA-seq analysis, the negative binormal distribution model test is used through Bioconductor/edgeR package with corrected q-value as statistical cutoff.

### Reporting summary

Further information on research design is available in the Nature Research Reporting Summary linked to this article.

## Supplementary information

Supplementary Information

Peer Review File

Supplementary Table 1

Supplemental Table 2

Supplemental Table 3

Supplemental Table 4

Supplementary Table 5

Supplementary Table 6

Supplementary Table 7

Supplementary Table 8

Reporting Summary

## Data Availability

All relevant data supporting the key findings of this study are available within the article, and its Supplementary Information files, source files, or from the corresponding author upon reasonable request. The next-generating sequencing data are deposited at GEO database (GSE145730) [https://www.ncbi.nlm.nih.gov/geo/query/acc.cgi?acc=GSE145730]. Source data are provided with this paper.

## Source data

Source Data
