# Supplementary material for: Topoisomerase 3β knockout mice show transcriptional and behavioural impairments associated with neurogenesis and synaptic plasticity
Source: Nat Commun. 2020 Jun 19;11:3143. doi: 10.1038/s41467-020-16884-4 (PMC7305123; doi:10.1038/s41467-020-16884-4)
Supplement: Supplementary file 1 — Supplementary Information [file 41467_2020_16884_MOESM1_ESM.pdf]

Topoisomerase 3β knockout mice show transcriptional and behavioural impairments associated with neurogenesis and synaptic plasticity

Joo et al.

Inventory of Supplementary Information

Supplementary Figures 1-18 and Legends

Supplementary Figure 1: Paradigms of fear conditioning and context discrimination tests
Supplementary Figure 2: Top3β-KO mice show no significant abnormality in several behavior assays.
Supplementary Figure 3: Top3β-KO mice have defective proliferation of adult neural stem cells *in vitro;* and enlarged ventricles.
Supplementary Figure 4: Pol II and TDRD3 ChIP-seq signals are induced by fear conditioning in neuronal early response genes; and this induction is defective in Top3β-KO mice.
Figure Supplementary 5: The elongation (pSer2) but not initiation (pSer5) form of Pol II is significantly reduced in Arc and Npas4 in Top3β-KO mice without or with fear conditioning treatment.
Supplementary Figure 6: RT-qPCR data show that induction of mRNAs of several NER genes by fear conditioning is significantly reduced in Top3β-KO mice.
Supplementary Figure 7: TDRD3 and Pol II co-bind some but not all neuronal activity- regulated enhancers of NER genes in response to fear conditioning.
Supplementary Figure 8: Fear conditioning significantly increased Pol II binding to TSS and exons of many neuronal early response genes in WT but not Top3β-KO mice.
Supplementary Figure 9: Top3β-KO mice have reduced induction of Pol II and RNA-seq signals for neuronal early response genes in response to fear conditioning.
Supplementary Figure 10: Correlation analyses show that TDRD3-bound genes have stronger dependence on Top3β in neuronal activity dependent transcription than TDRD3-unbound genes.
Supplementary Figure 11: Top3β-KO mice have reduced Pol II signals in genes important for dementia, learning and memory.
Supplementary Figure 12: Top3β-KO mice have reduced Pol II signals in multiple schizophrenia-related genes.
Supplementary Figure 13: Induction of genome-wide H3K27 acetylation by fear conditioning is largely normal in Top3β-KO mice; but this induction may be reduced at some specific genes.
Supplementary Figure 14: Top3β-KO mice have reduced Pol II signals in multiple anxiety disorder-related genes.
Supplementary Figure 15: Top3β-KO mice have reduced Pol II signals in genes important for synapse.
Supplementary Figure 16: Top3β-KO mice have reduced Pol II signals in multiple genes important for neurogenesis.
Supplementary Figure 17: About half of the genes activated by a Top2β poison (etoposide) show reduced Pol II signals in Top3β-KO mice under fear conditioning.
Supplementary Figure 18: Oligomer sequences for the RT-qPCR

Supplementary Tables 1-8

Supplementary Table 1: Top3β-KO mouse brain shows significant reduction of RNA polymerase II signals at many neuronal early response genes under fear conditioning based on ChIP-seq.
Supplementary Table 2: Genes with fear conditioning-induced TDRD3 binding strongly overlap with the genes that show reduction in Top3β-KO mice under fear conditioning.
Supplementary Table 3: Top3β-KO mouse brain shows significant reduction of Pol II signals at many learning and memory genes as shown by ChIP-seq.
Supplementary Table 4: Top3β-KO mouse brain exhibits significant reduction of Pol II signals for many schizophrenia-related genes as shown by ChIP-seq.
Supplementary Table 5: Top3β-KO mouse brain exhibits significant reduction of Pol II signals for many anxiety disorder-related genes as shown by ChIP-seq.
Supplementary Table 6: Top3β-KO mouse brain shows significant reduction of RNA polymerase II signals at many synapse-related genes under fear conditioning based on ChIP-seq.
Supplementary Table 7: Top3β-KO mouse brain exhibits significant reduction of Pol II signals for many neurogenesis-related genes as shown by ChIP-seq.
Supplementary Table 8: Top3β-KO mice have common and distinct phenotypes compared to autism and schizophrenia animal models.

Supplementary Figure 1. Paradigms of fear conditioning and context discrimination tests

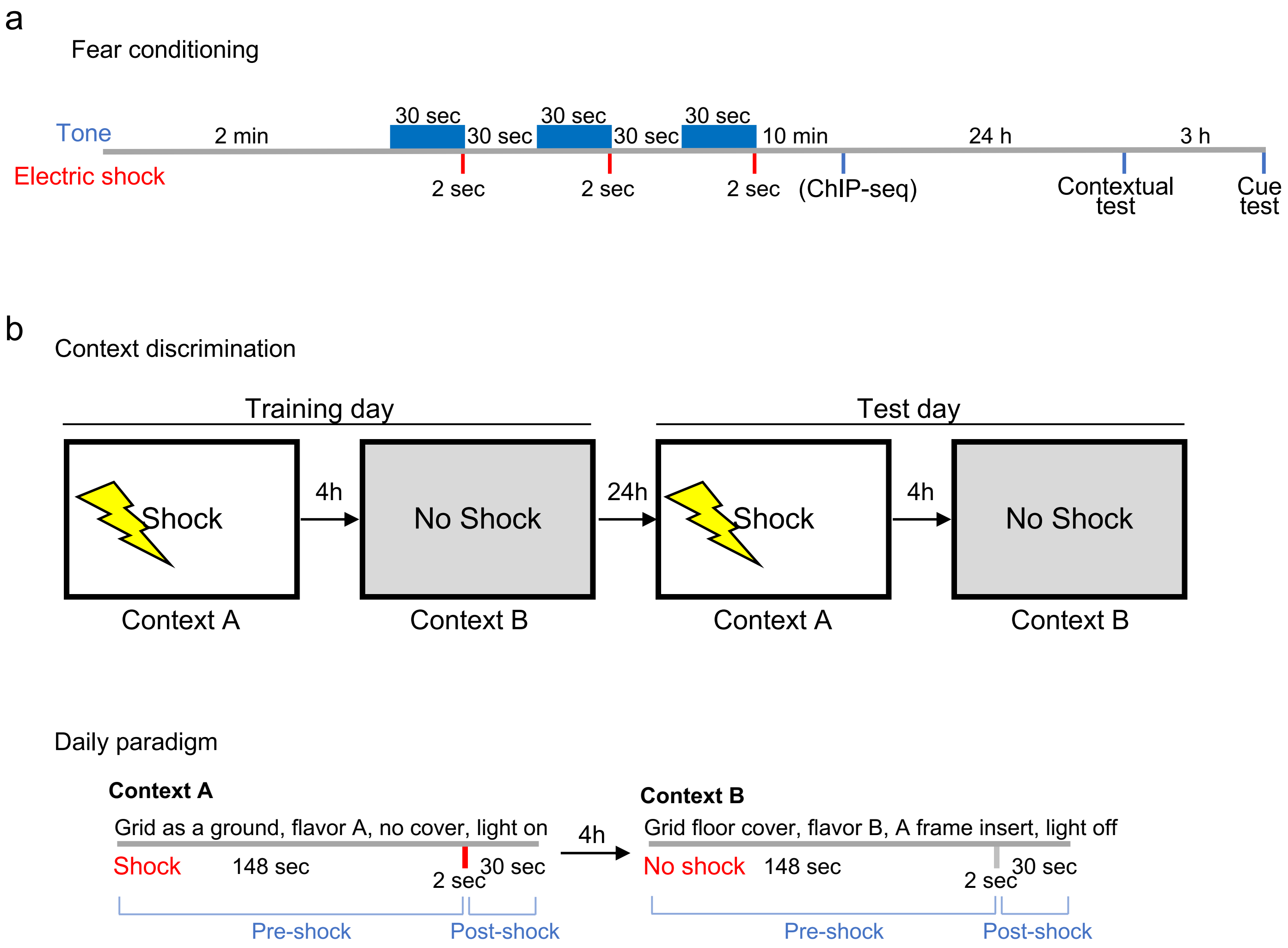

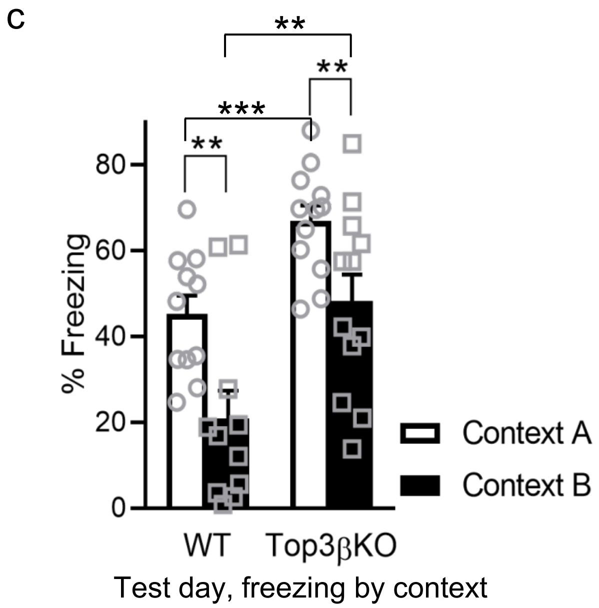

Supplementary Figure 1. Paradigms of fear conditioning and context discrimination tests. **(a)** A flowchart shows the paradigm of fear conditioning test. **(b)** Flow charts show the paradigm of context discrimination test. The top chart illustrates the two distinct contexts that are paired with the electric shock (context A) or a no-shock control (context B) in training and test days. The bottom illustrates the daily paradigm of testing and monitoring the mouse in the two contexts. **(c)** A graph shows that both WT and Top3β-KO mice have significant difference in percentage of freezing time in total time between Context A vs. B. 11 WT and 12 KO mice were used. 2-tail student T test was performed. Data are presented as mean values +/- SEM. Source data are provided as a Source Data file with detailed calculations.

Supplementary Figure 2. Top3β-KO mice show no significant abnormality in several behavior assays

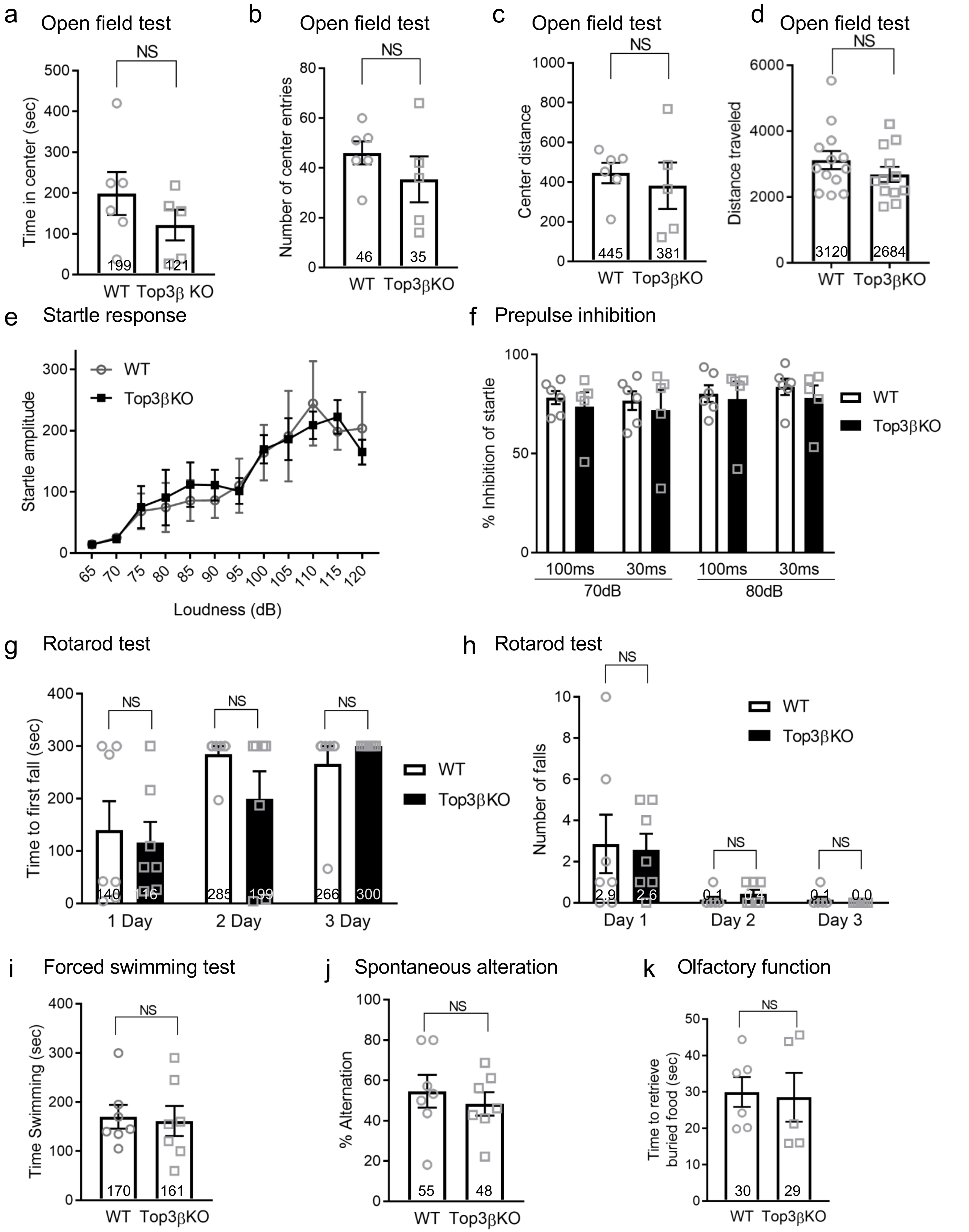

Supplementary Figure 2. Top3β-KO mice show no significant abnormality in several behavior assays. **(a-d)** Graphs show that Top3β-KO mice exhibit no significant differences in open field test ($p>0.05$). The length of time when mice stayed in the center of the field (a), the number of times when they entered the center (b), the distance which they traveled from the center (c), and the total distance traveled (d), are not significantly different from those of the WT. 6 WT and 5 KO were used for (a-c), and 13 WT and 12 KO were used for (d). **(e)** A graph shows that Top3β-KO mice display normal behaviors in startle response test, as they display a similar response curve in amplitude vs. the loudness of the noise in decibel (dB). **(f)** A graph shows that Top3β-KO mice have normal behaviors in Prepulse-inhibition test, as the percentage of inhibition of the startle by the prepulse is not significantly different between mutant and WT mice. The strength and length of the prepulse was indicated at X-axis. 6 WT and 5 KO were used. **(g, h)** Graphs show that Top3β-KO mice exhibit normal behaviors in Rotarod test, as the time to the first fall (g) and the number of the falls (h) are not significantly different between mutant and WT mice. (N=7) **(i)** A graph illustrates that normal behaviors of Top3β-KO mice in forced swimming test, measured by the time of the swimming. (N=7) **(j)** A graph shows that normal behaviors of Top3β-KO mice in spontaneous alteration test. (N=7) **(k)** A graph shows normal olfactory function of Top3β-KO mice. This is measured using the time to retrieve the buried food. 6 WT and 5 KO were used. Averages are indicated inside each bar. Error bars represent standard errors. Two-tail Student T-test was used to calculate p-values in all graphs. NS: statistically not significant ($p>0.05$). Data are presented as mean values +/- SEM. Source data are provided as a Source Data file with detailed calculations.

Supplementary Figure 3. Top3β-KO mice have defective proliferation of adult neural stem cells *in vitro*, and enlarged ventricles

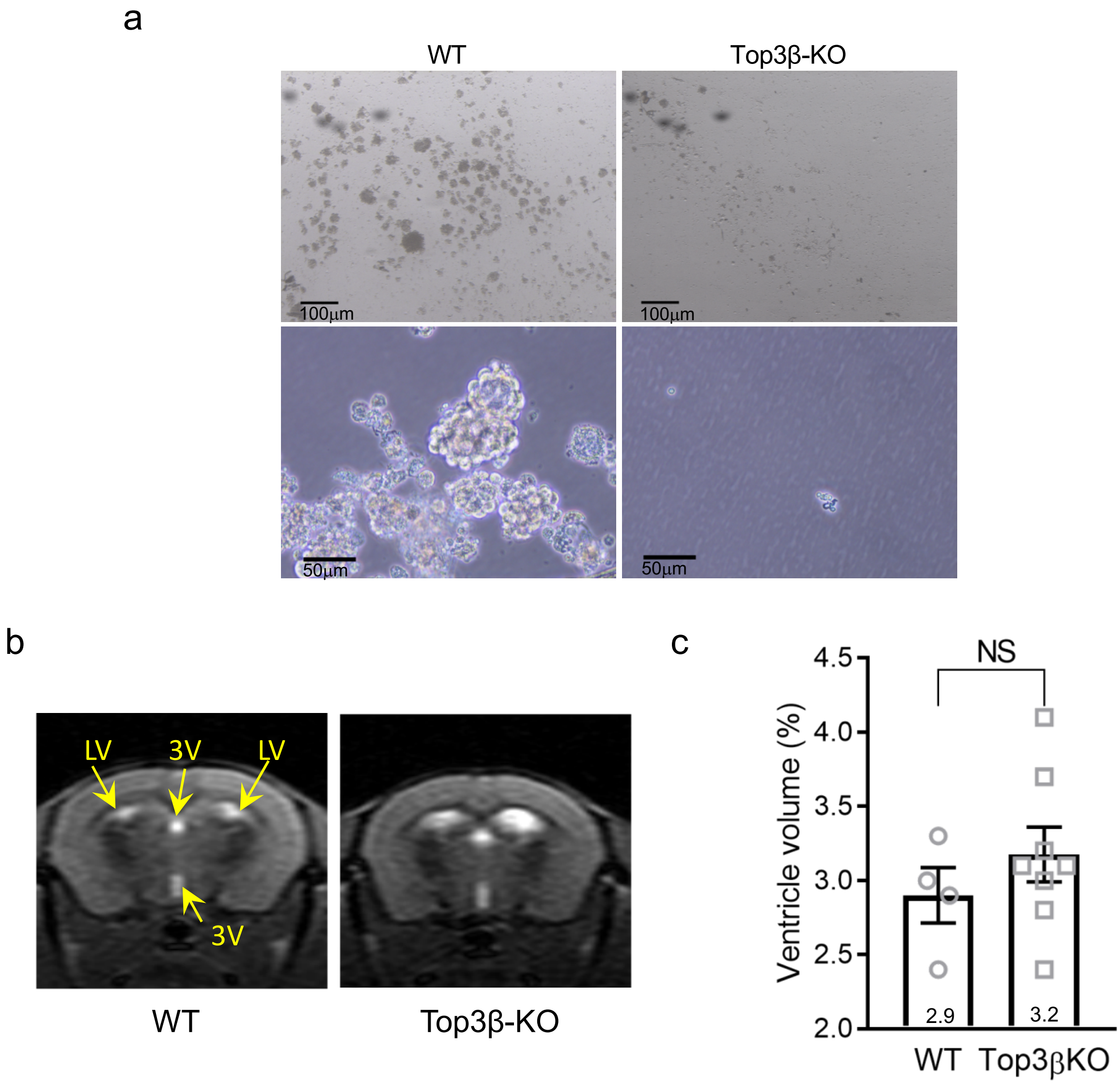

Supplementary Figure 3. (a) Representative images of *in vitro* cultured adult neural stem cells isolated from hippocampus of WT and Top3β-KO cells. The cells from Top3β-KO mice grew poorly and died after 7 days, in contrast to those from WT mice, which proliferate normally. This was confirmed with 4 mice per each group. **(b)** Representative images show enlarged ventricles in Top3β-KO mice vs. those from WT. Inversion-prepared, T1-weighted spin echo MR images of the mouse brain are shown. At 7 Tesla (300 MHz for 1H), inversion time TI = 604 ms results in dark contrast for white matter, bright contrast for cerebrospinal fluid (CSF) and gray contrast for gray matter. Images shown are for a 1 mm thick coronal slice 1 mm caudal from bregma. Bright areas correspond to CSF in the lateral ventricles (LV) and third ventricle (3V), as indicated by arrows. An image acquired for a Top3β-KO mouse (right) shows enlarged ventricles when compared to an age-matched WT mouse (left). The percentage of ventricle volume in total brain volume is shown in **(c)**. 4 WT and 8 KO were used. Data are presented as mean values +/- SEM. NS represents statistically not significant difference (p-value>0.05). Two-tail Student T-test was performed for statistical analysis. Source data are provided as a Source Data file with detailed calculations.

Supplementary Figure 4. Pol II and TDRD3 ChIP-seq signals are induced by fear conditioning in neuronal early response genes; and this induction is defective in Top3β-KO mice

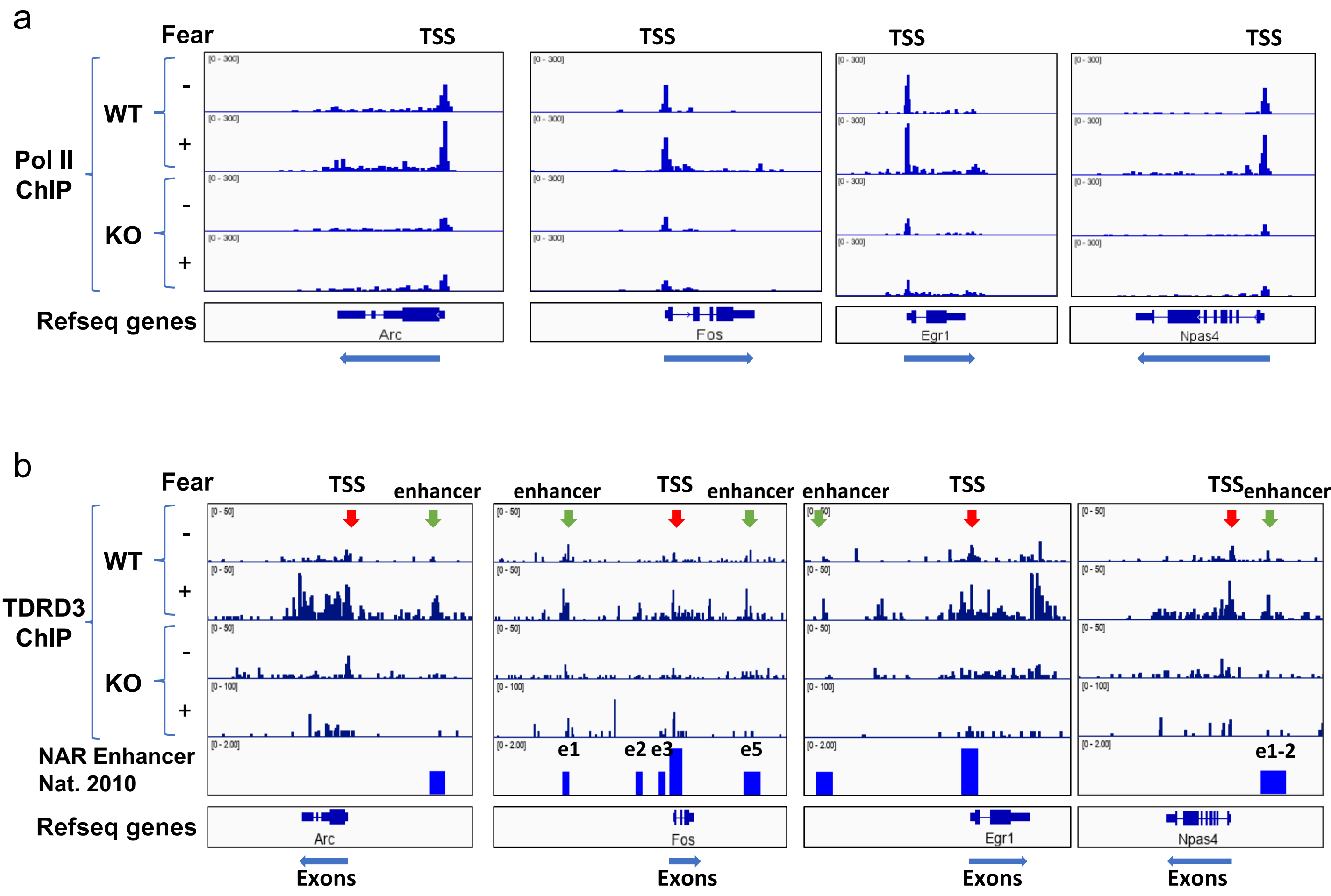

Supplementary Figure 4. (a) Bedgraphs show that 4 NER genes, *Arc, Fos, Egr1 and Npas4*, have FC-induced Pol II ChIP signals at TSS in WT mouse brain; and this induction is defective in Top3β-KO mice. **(b)** Bedgraphs show FC-induced TDRD3 signals at TSS, enhancers and exons of the same 4 NER genes, which are marked by red, green, and blue arrows. Neuronal activity regulated (NAR) enhancers identified in cultured neurons by Greenberg and colleagues (Kim et al., Nature 2010) were included as markers. The colocalization of TDRD3 and Pol II at Fos and Npas4 enhancers are further shown in bedgraphs in Supplementary Figure 7.

Supplementary Figure 5. The elongation (pSer2) but not initiation (pSer5) form of Pol II is significantly reduced in Arc and Npas4 in Top3β-KO mice without or with fear conditioning treatment

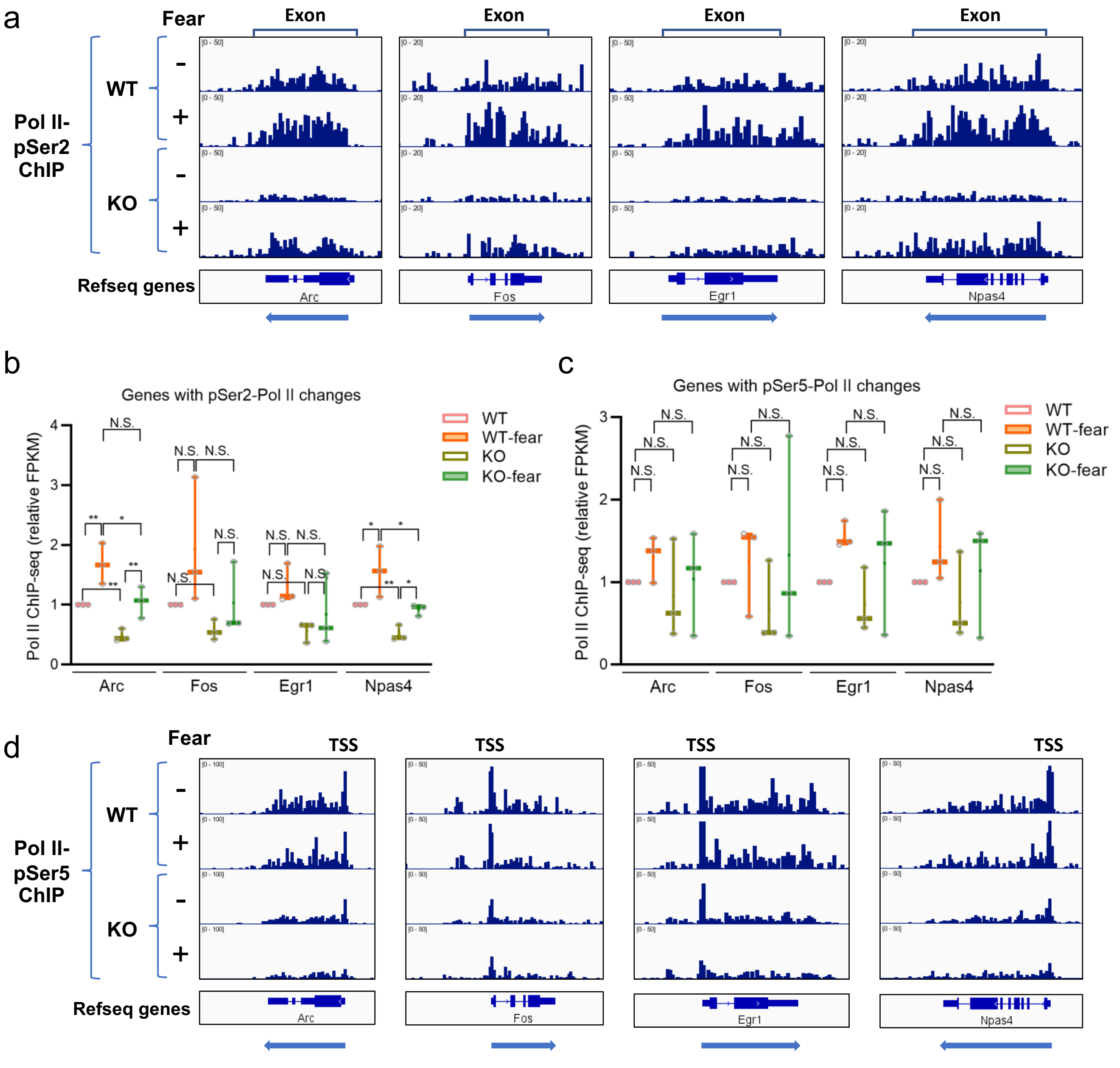

Supplementary Figure 5. (a, b) Bedgraphs (a) and bar graphs (b) show that 4 NER genes have FC-induced phosphorylated Pol II-Ser2 ChIP signals at all exons in WT mouse brain; and this induction is reduced in Top3β-KO mice. The graph (b) also shows significantly reduced signals of phosphorylated Pol II-Ser2 ChIP signals for Arc and Npas4 in Top3β-KO mice without or with FC treatment (p<0.05), but the reduction is not significant for Fos and Egr1. **(c, d)** same as (b, a) except that phosphorylated Pol II-Ser5 ChIP signals are shown. The graph (c) reveals no significant difference (N.S.) (p>0.05), in comparison between FC vs. no treatment, or between Top3β-KO vs. WT mice. 3 mice per group were used. P-values <0.05, 0.01, and 0.001 are marked as: *, **, ***, respectively. 2-way ANOVA test was used in statistical analysis. The Whiskers mark the highest and lowest of data; the box marks the first and third quantile; and the center line marks the median. Source data are provided as a Source Data file with detailed calculations and p-values.

Supplementary Figure 6. RT-qPCR data show that induction of mRNAs of several NER genes by fear conditioning is significantly reduced in Top3β-KO mice

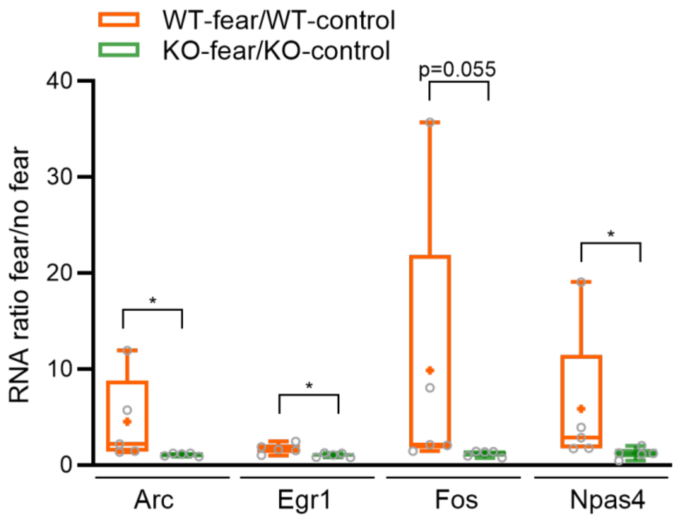

Supplementary Figure 6. Graph of RT-qPCR data shows that induction of mRNAs of several NER genes by fear conditioning is significantly reduced in Top3β-KO mice. There is also a strong trend of reduced induction of c-fos mRNA, but the reduction does not reach statistical significance (p=0.055). The Whiskers mark the highest and lowest of data; the box marks the first and third quantile; and the center line marks the median. RT-qPCR was performed from 5 independent sets of WT and Top3β-KO mice, which are untreated or treated by fear conditioning. The ratios between RT-qPCR signals of treated vs. untreated mice, fear/no fear, were calculated and shown in the graphs. These ratios were log-transformed, and analyzed by 2-tail Student T-test. P-value<0.05 is marked by "*". The P-value for *Fos* mRNA difference is very close to be statistically significant (p=0.055). Source data are provided as a Source Data file with detailed calculations.

Supplementary Figure 7. TDRD3 and Pol II co-bind some but not all neuronal activity- regulated enhancers of NER genes in response to fear conditioning

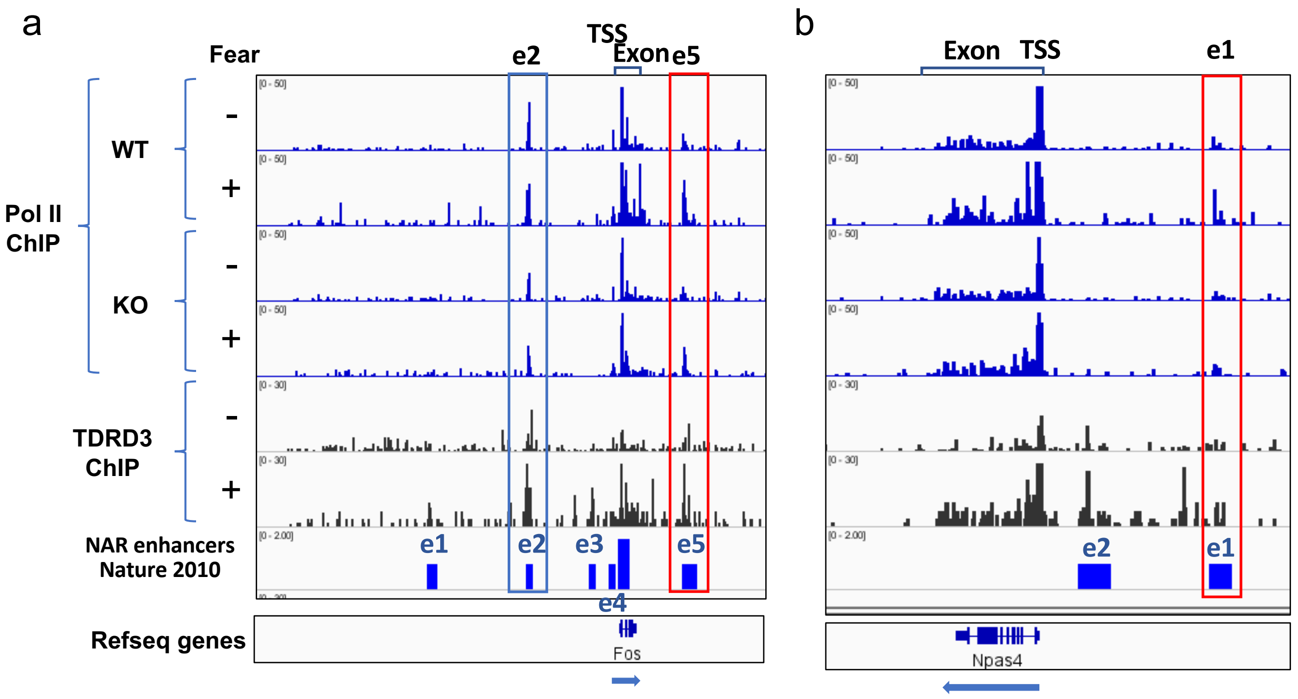

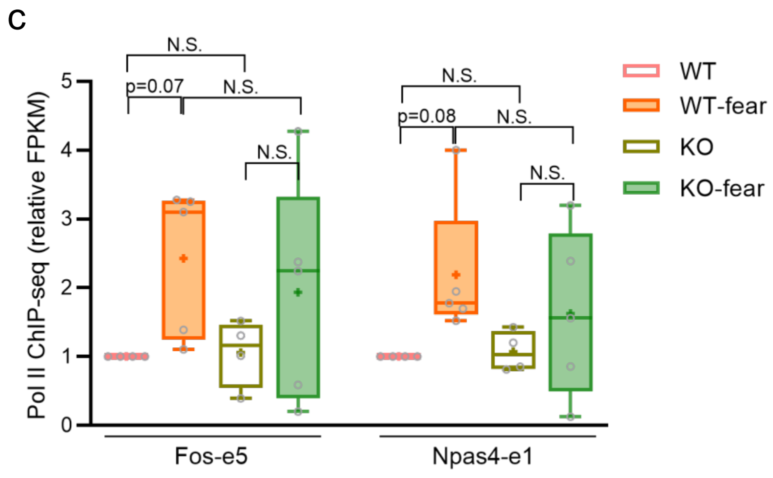

Supplementary Figure 7. (a-b) Representative bedgraphs of Fos and Npas4 loci show that fear conditioning-induced TDRD3 and Pol II ChIP signals colocalize at some but not all its neuronal activity-regulated (NAR)-enhancers (Kim et al., Nature 2010) in WT mice. The enhancers are marked as e1-e5. The FC-induced TDRD3 and Pol II signals at one enhancer for each gene (Fos-e5 and Npas4-e1; marked by a red box) showed localization in WT mouse brains; and this induction of Pol II signals is reduced in Top3β-KO mice. The reduced induction of TDRD3 signals in Top3β-KO mice are shown in Figure S4b. **(c)** A box-Whisker graph show that Pol II signals at Fos-e5 and Npas4-e1 display a strong trend of induction by FC (p=0.07-0.08) in WT mice. The data were derived from 4 pairs of untreated mice, and 5 pairs of FC-treated mice. No significant difference (p>0.05) is marked as "N.S.". 2-way ANOVA test was used in statistical analysis. The Whiskers mark the highest and lowest of data; the box marks the first and third quantile; and the center line marks the median. Source data are provided as a Source Data file with detailed calculations.

Supplementary Figure 8. Fear conditioning significantly increased Pol II binding to TSS and exons of many neuronal early response genes in WT but not Top3β-KO mice

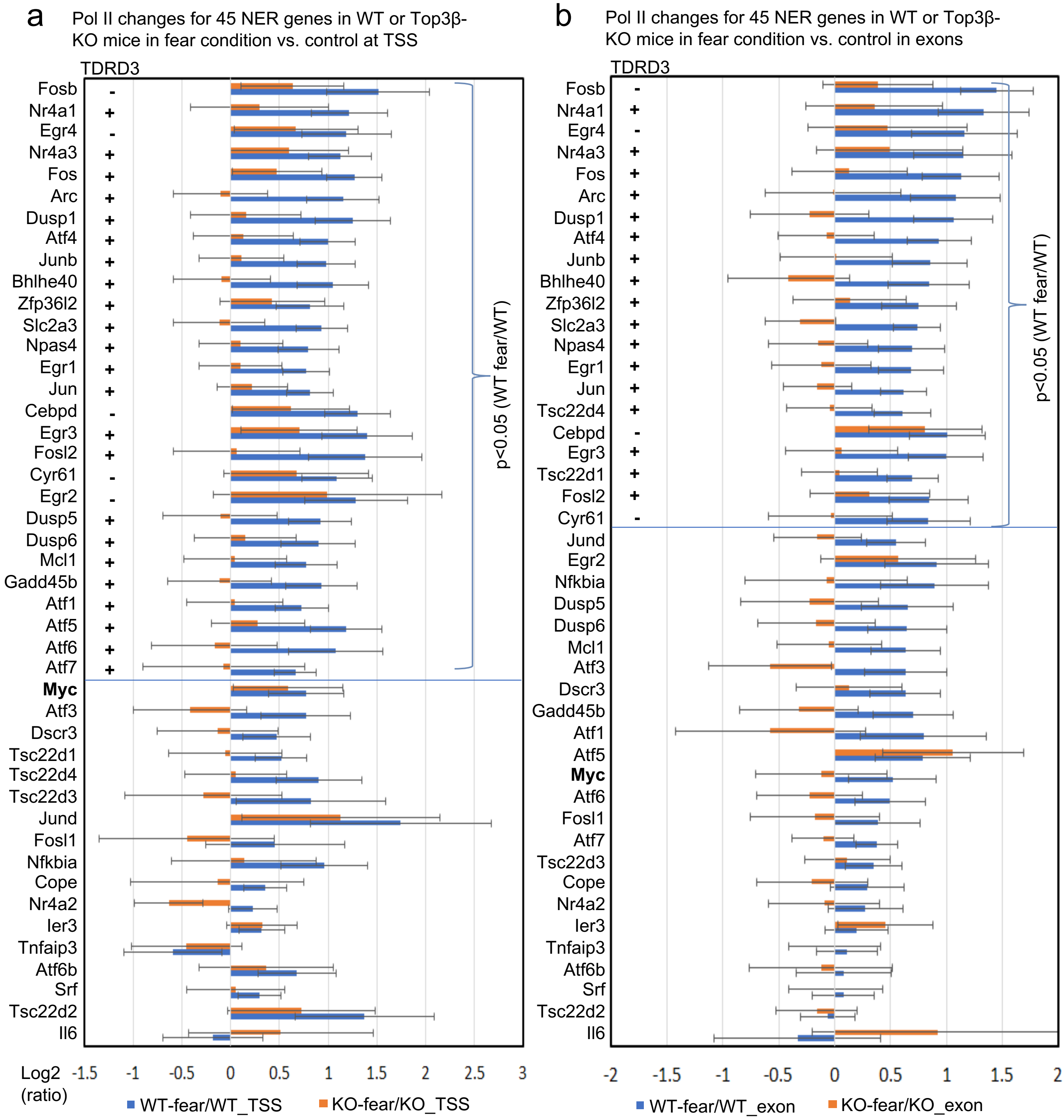

Supplementary Figure 8. (a) A graph shows that 28 of the 45 NER genes display FC-induced Pol II signals at their TSS in WT (marked by blue bracket and corresponding p-values). In contrast, no gene shows significant Pol II induction in Top3β-KO mice. The presence (+) or absence (-) of TDRD3 binding is indicated on the left. **(b)** Same as (a), except Pol II data at exons are analyzed, and only 21 NER genes show FC-induced increase in WT but not Top3β-KO mice. The data were derived from 4 pairs of untreated, and 5 pairs of FC-treated mice. Error bars represent standard errors (ANOVA). 2-way ANOVA test was performed. Source data are provided as a Source Data file with detailed calculations. We did not see significant reduction in *Myc* gene, which was proposed to depend on Top3b to stimulate its transcription (Yang et al., Mol. Cell., 2014).

Supplementary Figure 9. Top3β-KO mice have reduced induction of Pol II and RNA-seq signals for neuronal early response genes in response to fear conditioning

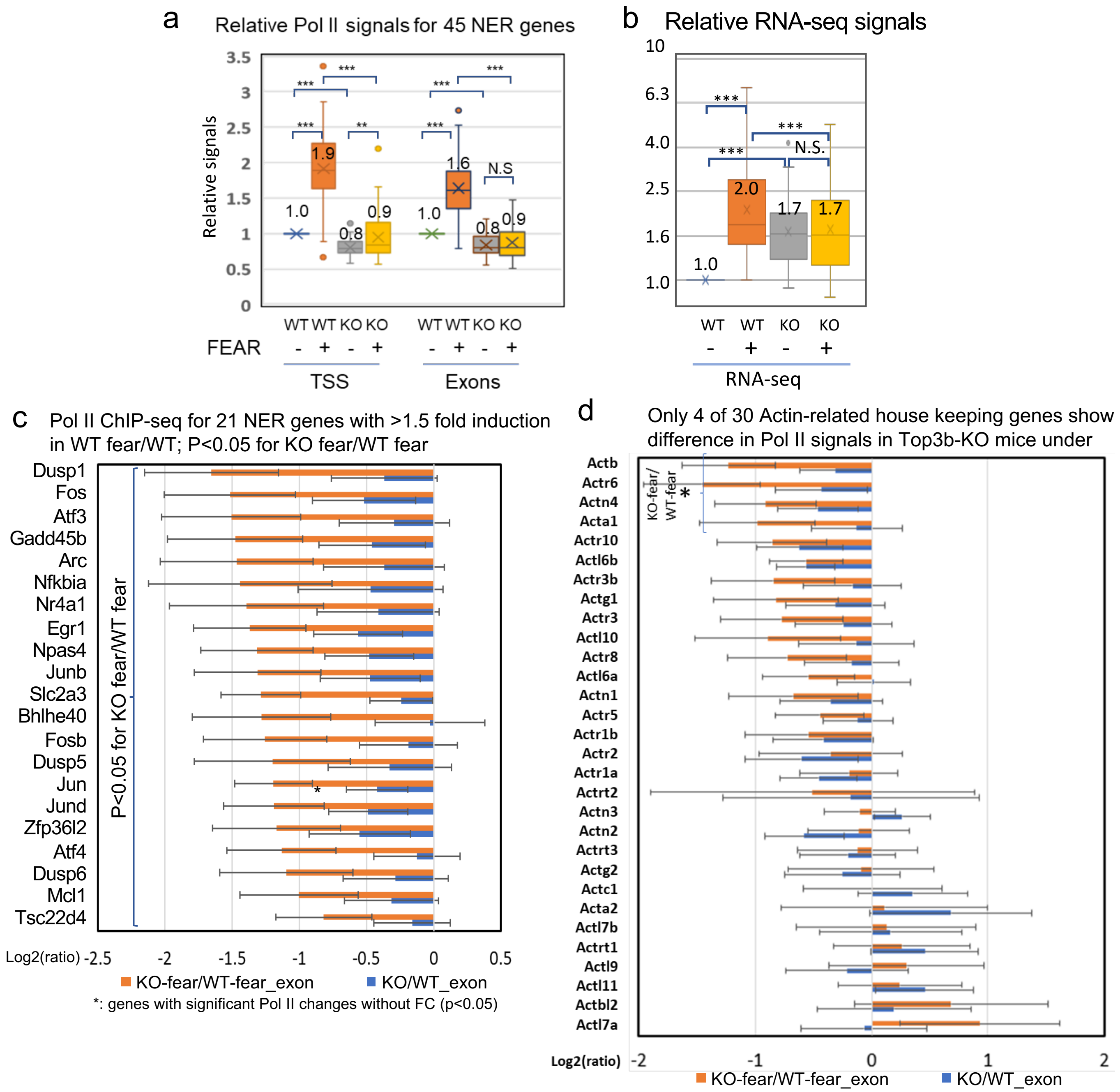

Supplementary Figure 9. (a) A box-Whisker graph illustrates relative Pol II ChIP signals at TSS and exons of 45 neuronal early response (NER) genes in WT and Top3β-KO mice, without or with fear conditioning treatment, as indicated. The data used geometric means were derived from 4 pairs of untreated mice, and 5 pairs of FC-treated mice. They are normalized using data from untreated WT mice as the standard. 2-tail student T-test was used. The Whiskers mark the highest and lowest of data; the box marks the first and third quantile; and the center line marks the median. **(b)** Same as (a) except Total RNA-seq data were analyzed from 2 sets of untreated or FC treated mice. **(c)** A graph shows 21 NER genes that have at least 1.5-fold induction of Pol II signals in WT mice, and significant reduction in Top3β-KO mice under fear conditioning (p<0.05). Log2_ratios of Pol II signals between fear and no fear, or between Top3β-KO and WT are shown in X-axis. **(d)** same as (c) except 30 actin-related house-keeping genes are analyzed, and only 4 genes showed significant difference. Asterisks mark genes that also show significant difference (p<0.05) using data without FC. P-values <0.05 is marked as: *. 2-way ANOVA test was used in statistical analysis for (c-d). Source data are provided as a Source Data file with detailed calculations.

Supplementary Figure 10. Correlation analyses show that TDRD3-bound genes have stronger dependence on Top3β in neuronal activity dependent transcription than TDRD3-unbound genes

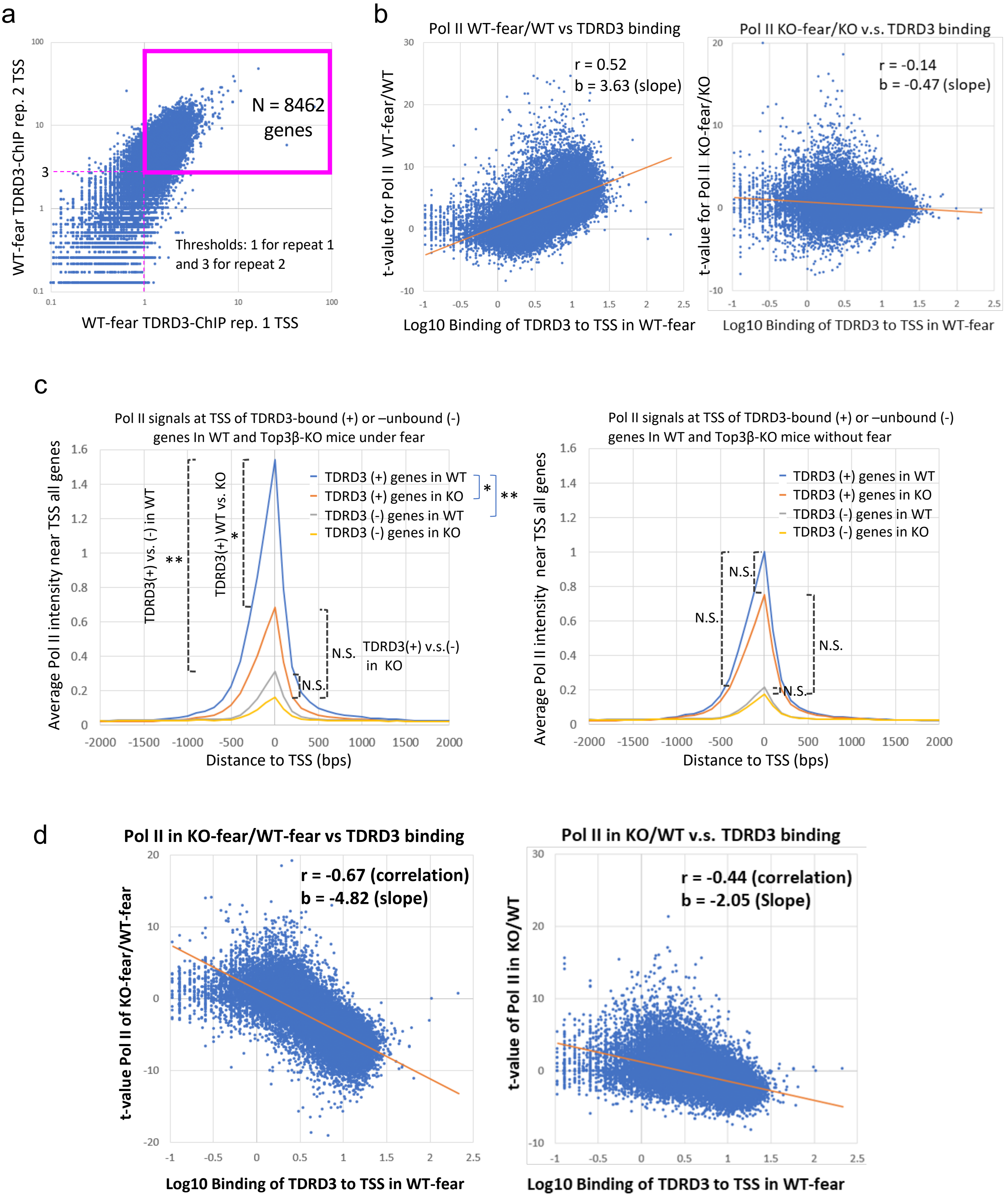

Supplementary Figure 10. (a) A scattered plot depicts selection of TDRD3-bound genes based on two independent TDRD3 ChIP-seq signals at TSS from WT mice (replicate 1 and 2). The threshold is 1 and 3 for the replicate 1 and 3, respectively. The box marks the TDRD3 ChIP datapoints from 8462 genes that meet the threshold. The detailed data are in Table S2. **(b)** Two scattered plots show a modest correlation between TDRD3 signals at TSS in WT mice under FC and the change of Pol II signals induced by FC in WT mice (left); but no correlation for the same data in Top3β-KO mice (right). Correlation efficient (r) and slope are indicated. T-value indicates normalized difference of the signal (mean divided by standard deviation). **(c)** Two graphs show that Pol II signals at TSS of TDRD3-bound or -unbound genes in FC-treated (left) or untreated (right) mice. No significant difference ($p>0.05$) is marked as "N.S.". P-values <0.05 and 0.01 are marked as: * and **, respectively. 2-way ANOVA test was used in statistical analysis. Note that only TDRD3-bound genes show significant difference between WT and Top3β-KO mice under FC. There is also significant difference between TDRD3-bound and unbound genes under FC. **(d)** Two scattered plots depict a negative correlation between TDRD3 signals and the ratio of Pol II signals of Top3β-KO vs. WT mice with fear conditioning (left), or without treatment (right). The calculation of T values is the same as (b). Source data are provided as a Source Data file with detailed calculations.

Supplementary Figure 11. Top3β-KO mice have reduced Pol II signals in genes important for dementia, learning and memory

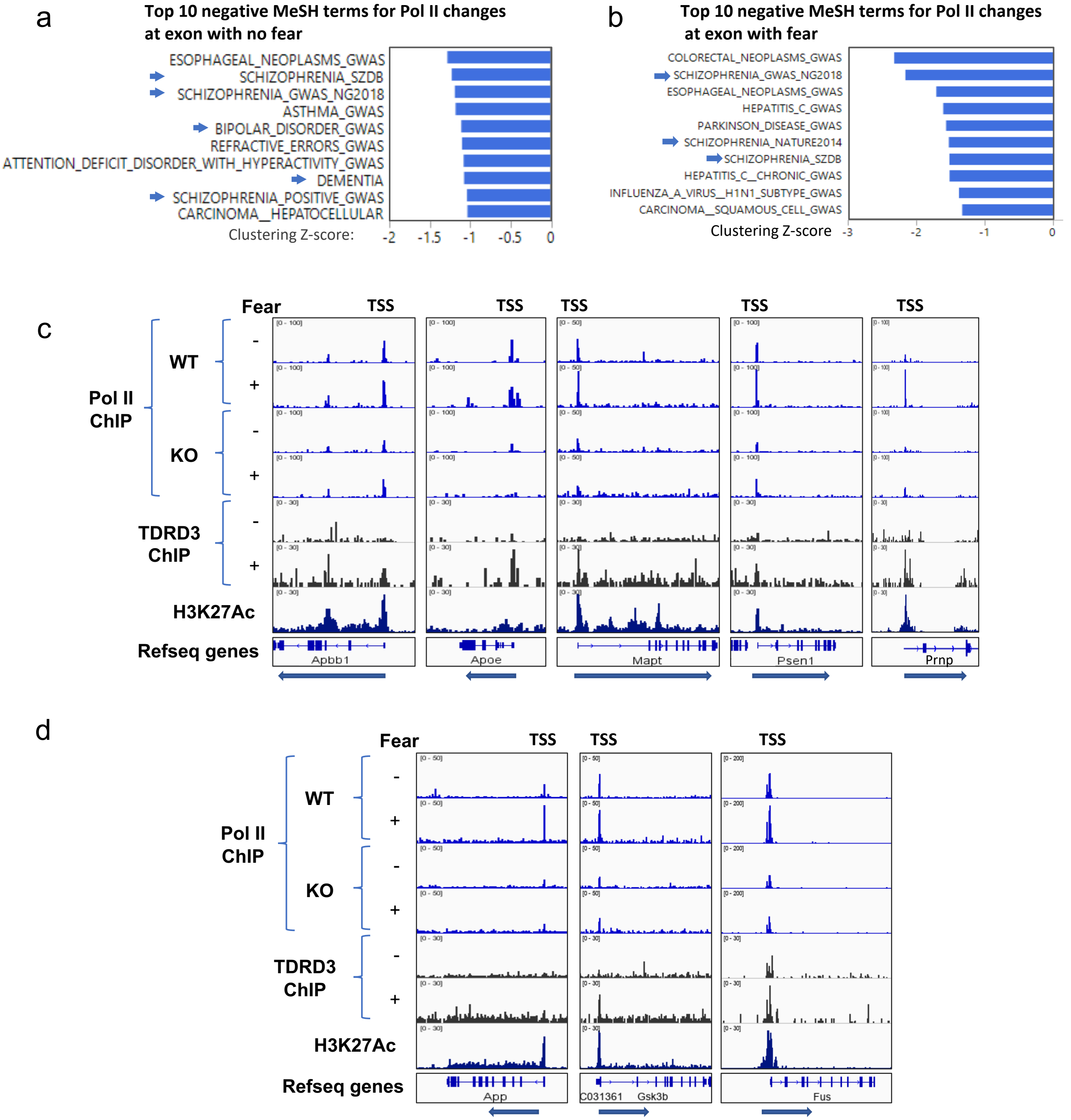

Supplementary Figure 11. (a, b) A list of top10 negative MeSH terms (gene sets) identified by PAGE (Parametric Analysis of Gene set Enrichment) analysis, using log 2-fold change between Pol II signals in all exons of Top3β-KO vs. those of WT mice without (a) or with (b) FC treatment. Arrows indicate MeSH terms related to mental disorders. **(c, d)** Bedgraphs show reduction of Pol II signals in 5 genes critical for dementia, including Alzheimer's disease; and 3 genes important for learning and memory (d), in Top3β-KO mice. The TDRD3 ChIP data are included to show the FC-induced TDRD3 peak at TSS where it co-localizes with that of Pol II. The presence or absence FC treatment is indicated on the left. H3K27Ac ChIP data are also added to mark TSS.

Supplementary Figure 12. Top3β-KO mice have reduced Pol II signals in multiple schizophrenia-related genes

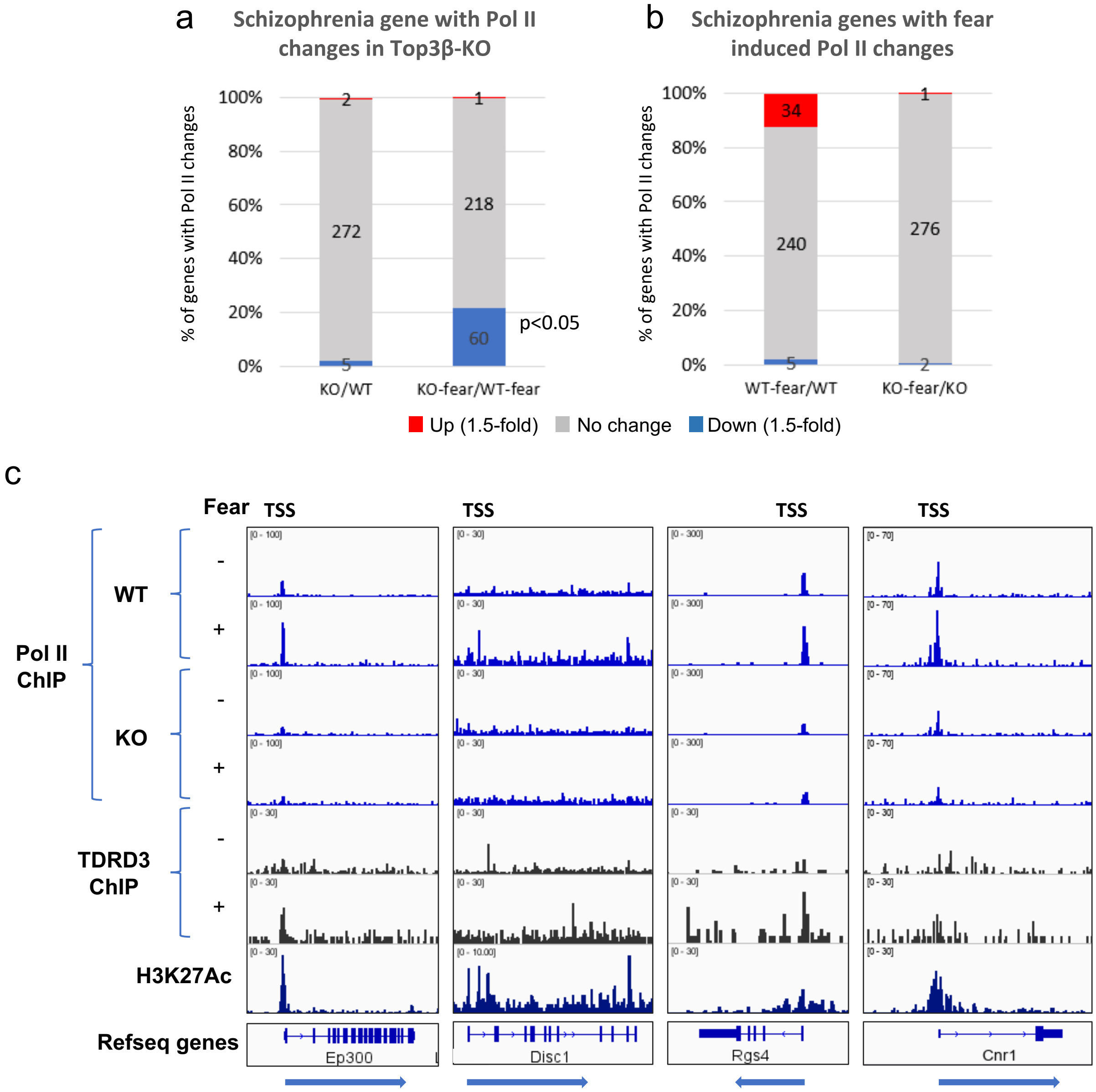

Supplementary Figure 12. (a) A graph shows that the number and percentage of schizophrenia-related genes downloaded from SZDB that are either upregulated, no change, or down regulated, of Pol II signals in Top3β-KO vs. WT mice, without or with treatment of fear conditioning. The cut-off threshold is 1.5-fold ($p<0.05$). 2-way ANOVA test was performed. **(b)** A graph shows the number of schizophrenia-related genes that are upregulated, no change, or downregulated, by fear conditioning in WT or Top3β-KO mice. **(c)** Bedgraphs show reduction of Pol II signals in 4 genes related to schizophrenia in Top3β-KO mice. The TDRD3 ChIP data are included to show the FC-induced TDRD3 peak at TSS where it overlaps with that of Pol II. The presence or absence of FC treatment is indicated on the left. H3K27Ac ChIP data are also added to mark TSS. Source data are provided as a Source Data file with detailed calculations.

Supplementary Figure 13. Induction of genome-wide H3K27 acetylation by fear conditioning is largely normal in Top3β-KO mice; but this induction is reduced at some specific genes

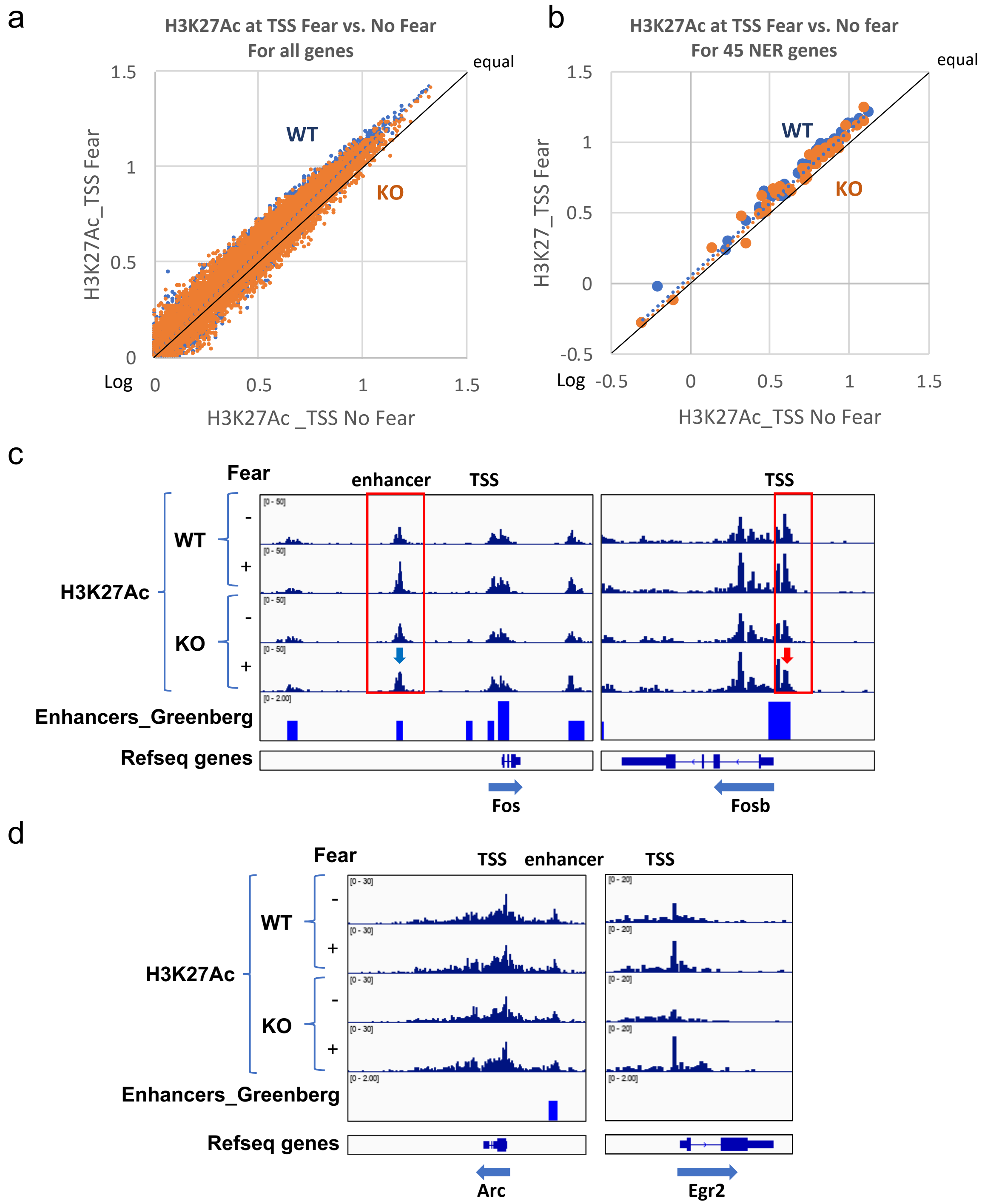

Supplementary Figure 13. (a-b) Scattered plots show FC-induced H3K27Ac mark is similar between Top3β-KO and WT mice for all genes (a), or 45 NER genes (b). Majority of data points and the trend lines for both genotypes are above the equal line and exhibit similar patterns, indicating that induction of H3K27Ac is largely normal in Top3β-KO mice. **(c-d)**. Bedgrahs show that induction of H3K27Ac at two NER genes, Fos and Fosb, is modestly reduced at TSS or enhancers in Top3β-KO mice (c), but this induction is largely normal in Arc and Egr2 genes. The red box and arrows indicate reduction of H3K27Ac peak. The enhancers reported (Kim et al., Nature 2010) are included as markers. Source data are provided as a Source Data file with detailed calculations.

Supplementary Figure 14. Top3β-KO mice have reduced Pol II signals in multiple anxiety disorder-related genes

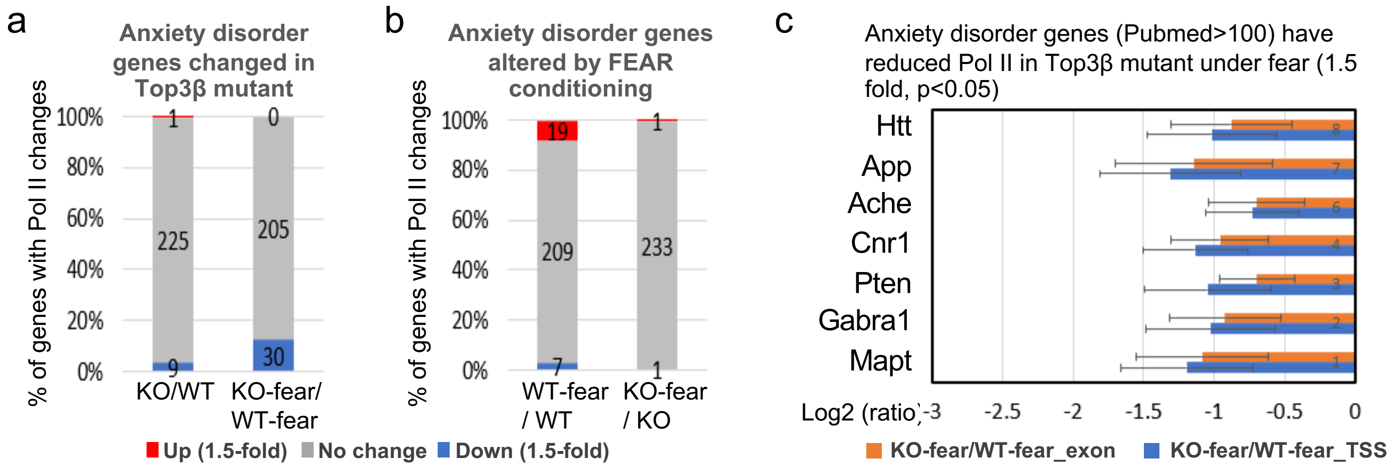

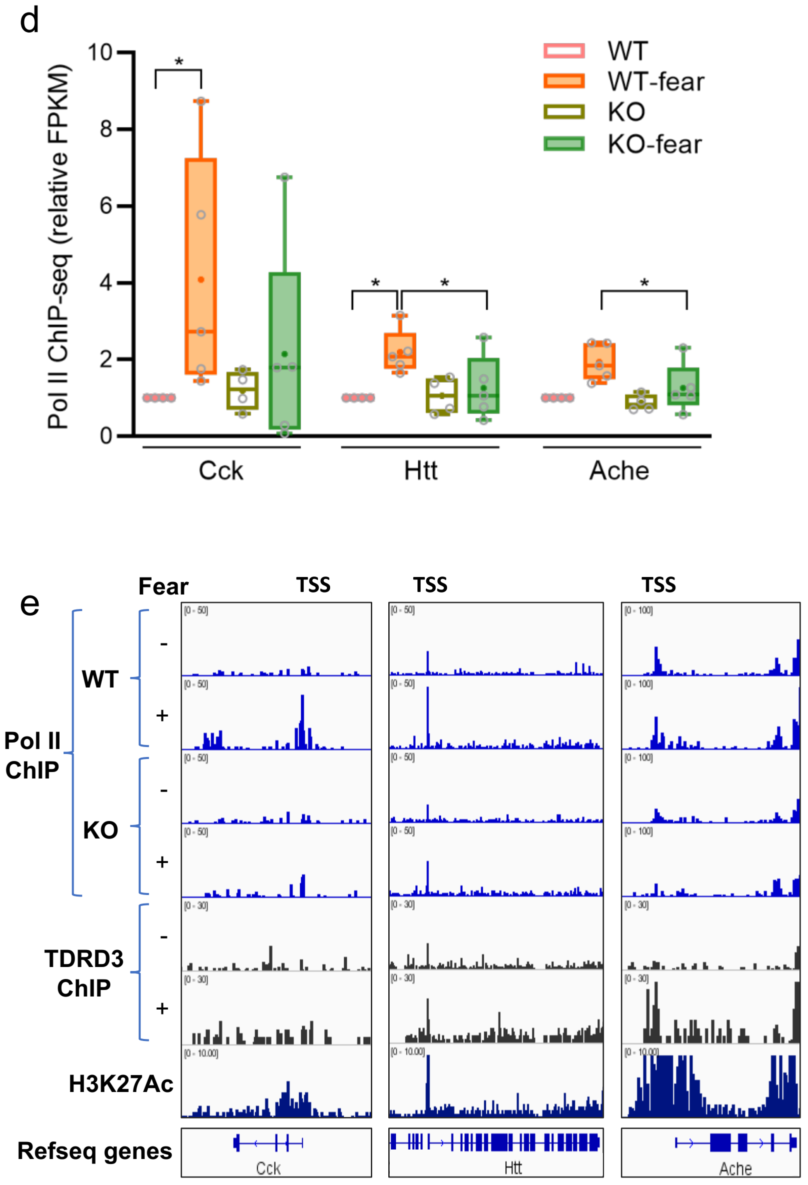

Supplementary Figure 14. (a) A graph shows the number of anxiety disorder-related genes that are upregulated, no change, or downregulated, in Top3β-KO mice without or with FC treatment. Threshold is 1.5 fold ($p<0.05$; 2 way ANOVA). **(b)** A graph shows the number of anxiety disorder-related genes that are upregulated, no change or downregulated by FC treatment in WT or Top3β-KO mice. **(c)** A graph shows 7 commonly-studied anxiety disorder-related genes (Pubmed citation>100) that have reduced Pol II signals at both exons and TSS in Top3β-KO mice under FC. Log2 ratios of the Pol II signals between Top3β-KO vs. WT mice are shown. Error bars represent standard errors (ANOVA). **(d)** A box-Whisker graph shows 3 commonly-studied anxiety disorder-related genes display lower Pol II signals and/or reduced FC-induced of Pol II signals. The Whiskers mark the highest and lowest of data; the box marks the first and third quantile; and the center line marks the median. 4 pairs of untreated mice, and 5 pairs of FC-treated mice were used for (c, d) **(e)** Representative bedgraphs show reduction of Pol II signals in 3 anxiety disorder-related genes in Top3β-KO mice. The TDRD3 ChIP data are included to show the FC-induced TDRD3 peak at TSS where it overlaps with that of Pol II. The presence or absence FC treatment is indicated on the left. H3K27Ac ChIP data are also added to mark TSS. P-values <0.05, 0.01, and 0.001 are marked as: *, **, ***, respectively. Two-way ANOVA was used in statistical analysis. Source data are provided as a Source Data file with detailed calculations.

Supplementary Figure 15. Top3β-KO mice have reduced Pol II signals in multiple genes important for synapse

a

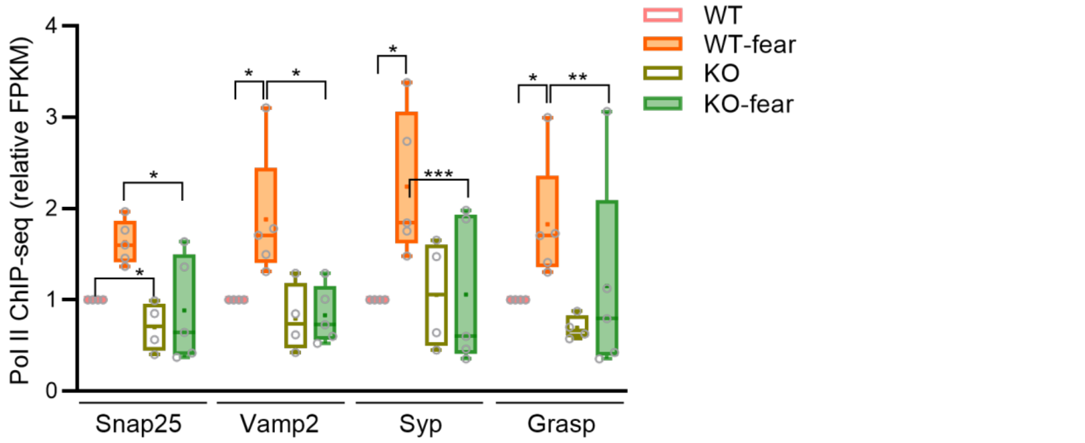

b

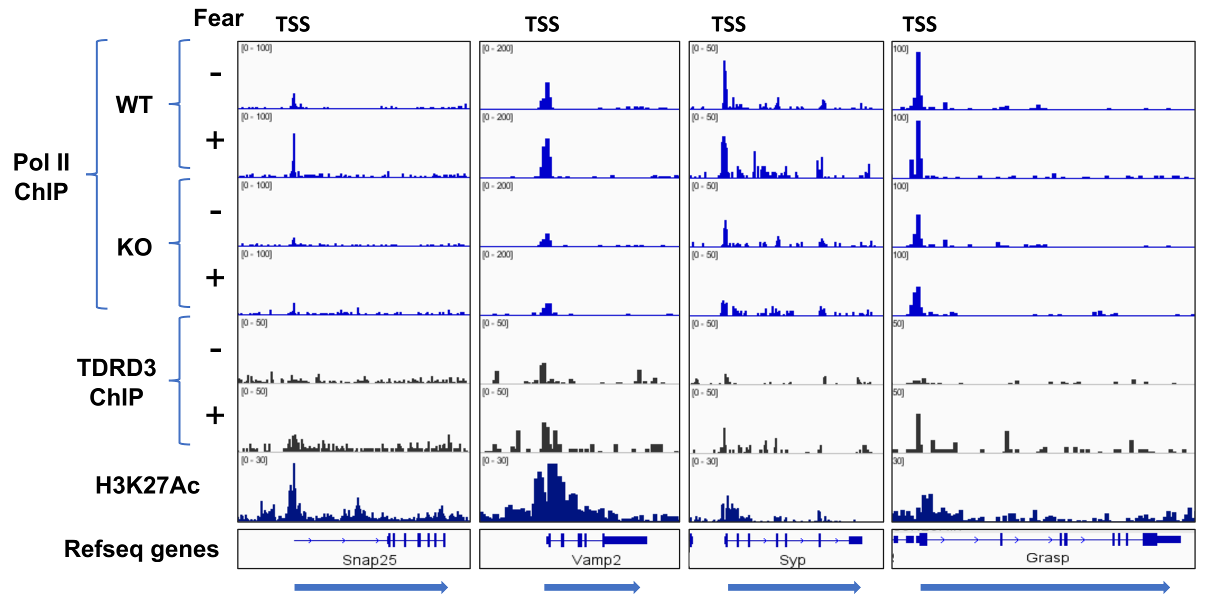

Supplementary Figure 15. (a, b) A Box-Whisker graph (a) and bedgraphs (b) show reduction of Pol II signals in 4 commonly-studied synapse-related genes (Pubmed citation>100) in Top3β-KO mice. The Whiskers mark the highest and lowest of data; the box marks the first and third quantile; and the center line marks the median. The TDRD3 ChIP data are included to show the FC-induced TDRD3 peak at TSS where it overlaps with that of Pol II. The presence or absence FC treatment is indicated on the left. 4 pairs of untreated mice, and 5 pairs of FC-treated mice were used for (a). H3K27Ac ChIP data are also added to mark TSS. P-values <0.05, 0.01, and 0.001 are marked as: *, **, ***, respectively. N.S. indicates not significant difference (p>0.05). 2-way ANOVA test was used in statistical analysis. Source data are provided as a Source Data file with detailed calculations.

Supplementary Figure 16. Top3β-KO mice have reduced Pol II signals in multiple genes important for neurogenesis

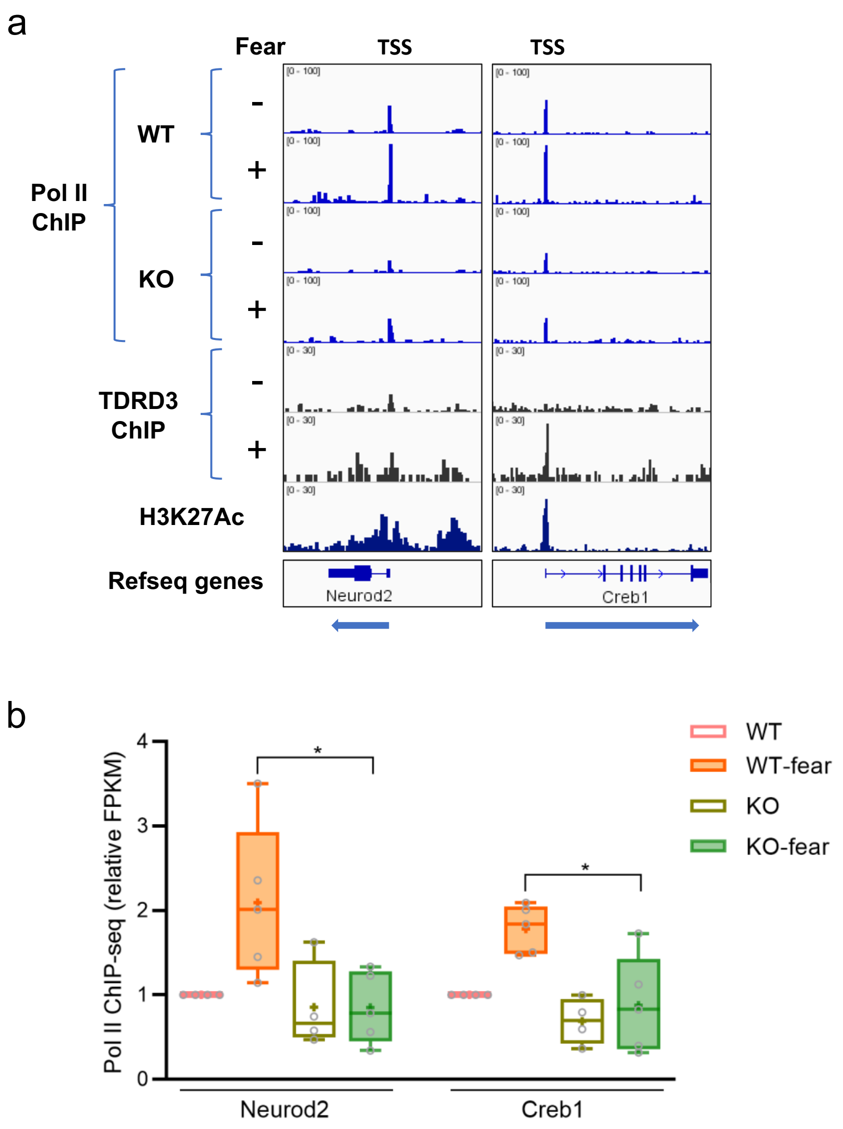

Supplementary Figure 16 (a, b) Bedgraphs (a) and Box-Whiker graph (b) show reduction of Pol II signals in 2 commonly-studied neurogenesis-related genes (Pubmed citations>20) in Top3β-KO mice. The Whiskers mark the highest and lowest of data; the box marks the first and third quantile; and the center line marks the median. The TDRD3 ChIP data are included to show the FC-induced TDRD3 peak at TSS where it overlaps with that of Pol II. The presence or absence of FC treatment is indicated on the left. 4 pairs of untreated mice, and 5 pairs of FC-treated mice were used. H3K27Ac ChIP data are also added to mark TSS. P-values <0.05, 0.01, and 0.001 are marked as: *, **, ***, respectively. N.S. indicates not significant difference (p>0.05). 2-way Anova test was used in statistical analysis. Source data are provided as a Source Data file with detailed calculations.

Supplementary Figure 17. About half of the genes activated by a Top2β poison (etoposide) show reduced Pol II signals in Top3β-KO mice under fear conditioning

a

Gene	log_2(fold change)	Pol II fold change WT-fear/Top3β-KO-fear Exon ($p<0.05$)
Fosb	4.36	2.4
Cort	3.79	Signal too low
Trim72	3.44	Signal too low
Plekhg4	2.65	Signal too low
Fos	2.46	2.9
1700094D03Rik	2.07	Signal too low
Nr4a2	1.97	1.7
Rpl35	1.87	1.7
Npas4	1.41	2.5
Sst	1.34	Signal too low
Dynlt1b	1.32	Signal too low
Rgs2	1.12	1.9

Madabhushi et al., Cell 2015, Fig. 1B

b

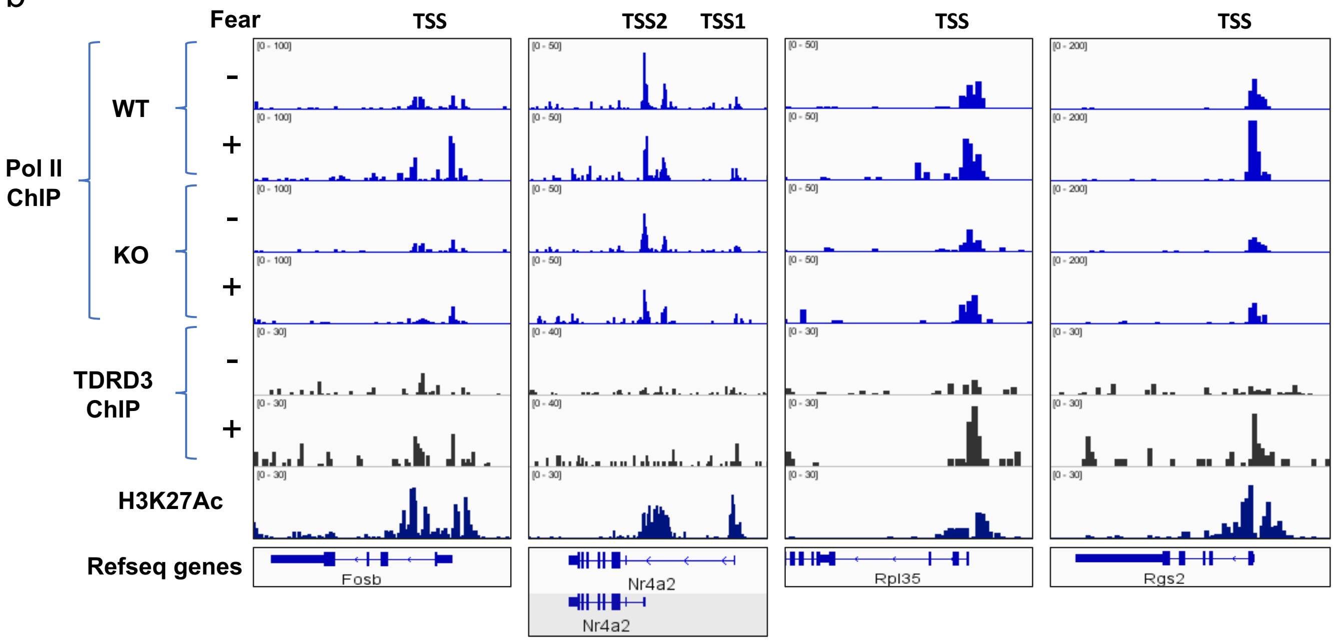

Bedgraphs for Fos and Npas4 are in Fig. 4a; and Fig. S4a

Supplementary Figure 17. (a, b) A table (a) and bedgraphs (b) show that 6 of the 12 genes activated by the Top2β poison, etoposide, exhibit reduced Pol II signals at exons in Top3β-KO mice under fear conditioning. The list of genes was from a previous publication (Madabhushi et al., Cell 2015, Fig. 1B). In (b), The treatment of fear conditioning is indicated on the left. The TDRD3 ChIP data are included to show the FC-induced TDRD3 peak at TSS where it overlaps with that of Pol II. H3K27Ac ChIP data are also added to mark TSS.

Supplementary Figure 18. Oligomer sequences for the RT-qPCR

Species	Gene	Set	Direction	Sequence
mouse	Arc	1	F	TGTGATCCTGCAGATTGGTAAG
mouse	Arc	1	R	CTTGGACACTTCGGTCAACA
mouse	Arc	2	F	TGAGGAGGAGGAGATCATTCA
mouse	Arc	2	R	CAGAAAGCGCTTGAGTTTGG
mouse	Egr1	1	F	GGAGAGGCAGGAAAGACATAAA
mouse	Egr1	1	R	GCTCTGAGATCTTCCATCTGAC
mouse	Egr1	2	F	AGGAGTGATGAACGCAAGAG
mouse	Egr1	2	R	GGATGGGTAAGAAGAGAGTGAAG
mouse	c-Fos	1	F	CAGCTCCCACCAGTGTCTAC
mouse	c-Fos	1	R	TCTGCGCAAAAGTCCTGTGT
mouse	c-Fos	2	F	GTGAAGACCGTGTCAGGAGG
mouse	c-Fos	2	R	GATCTGTCTCCGCTTGGAGT
mouse	Npas4	1	F	GACCCACTAACCAGTTCACTAC
mouse	Npas4	1	R	GCTTCTGGCTGAGCTTTCT
mouse	Npas4	2	F	GTCCTAATCTACCTGGGCTTTG
mouse	Npas4	2	R	TGTAGCAGTCCATACCATGATTTA
