# Supplementary material for: Topoisomerase 3β knockout mice show transcriptional and behavioural impairments associated with neurogenesis and synaptic plasticity
Source: Nat Commun. 2020 Jun 19;11:3143. doi: 10.1038/s41467-020-16884-4 (PMC7305123; doi:10.1038/s41467-020-16884-4)
Supplement: Supplementary file 11 — Reporting Summary [file 41467_2020_16884_MOESM11_ESM.pdf]

nature research

Corresponding author(s): Weidong Wang and Alexei Sharov

Last updated by author(s): Feb 14, 2020

Reporting Summary

Nature Research wishes to improve the reproducibility of the work that we publish. This form provides structure for consistency and transparency in reporting. For further information on Nature Research policies, see Authors & Referees and the Editorial Policy Checklist.

Statistics

For all statistical analyses, confirm that the following items are present in the figure legend, table legend, main text, or Methods section.

n/a	Confirmed	
☐	☒	The exact sample size (*n*) for each experimental group/condition, given as a discrete number and unit of measurement
☐	☒	A statement on whether measurements were taken from distinct samples or whether the same sample was measured repeatedly
☐	☒	The statistical test(s) used AND whether they are one- or two-sided<br>*Only common tests should be described solely by name; describe more complex techniques in the Methods section.*
☒	☐	A description of all covariates tested
☐	☒	A description of any assumptions or corrections, such as tests of normality and adjustment for multiple comparisons
☐	☒	A full description of the statistical parameters including central tendency (e.g. means) or other basic estimates (e.g. regression coefficient) AND variation (e.g. standard deviation) or associated estimates of uncertainty (e.g. confidence intervals)
☐	☒	For null hypothesis testing, the test statistic (e.g. F, t, r) with confidence intervals, effect sizes, degrees of freedom and P value noted<br>*Give P values as exact values whenever suitable.*
☒	☐	For Bayesian analysis, information on the choice of priors and Markov chain Monte Carlo settings
☒	☐	For hierarchical and complex designs, identification of the appropriate level for tests and full reporting of outcomes
☒	☐	Estimates of effect sizes (e.g. Cohen's *d*, Pearson's *r*), indicating how they were calculated

Our web collection on statistics for biologists contains articles on many of the points above.

Software and code

Policy information about availability of computer code

Data collection	The bcl files are converted to fastq files by bcl2fastq. The quality of the fastq files is checked by fastqc 0.11.8. The adapters in the files are trimmed by cutadapt 2.8. The files obtained are used for alignments.
Data analysis	The following softwares are used in data analysis: Tophat 2.1.1, samtools 1.9, bamtools 2.5.1, htseq 0.11.2, cufflinks 2.2.1, cuffdiff 2.2.1_patched, edgeR_3.26.8, limma_3.40.6, JMP13.2.1, and R.3.6.0.

For manuscripts utilizing custom algorithms or software that are central to the research but not yet described in published literature, software must be made available to editors/reviewers. We strongly encourage code deposition in a community repository (e.g. GitHub). See the Nature Research guidelines for submitting code & software for further information.

Data

Policy information about availability of data

All manuscripts must include a data availability statement. This statement should provide the following information, where applicable:
- Accession codes, unique identifiers, or web links for publicly available datasets
- A list of figures that have associated raw data
- A description of any restrictions on data availability

All relevant data supporting the key findings of this study are available within the article and its Supplemental Information files or from the corresponding authors upon reasonable request. The next-generating sequencing data are deposited at GEO database. The access code is GSE145730.

Field-specific reporting

Please select the one below that is the best fit for your research. If you are not sure, read the appropriate sections before making your selection.

☒ Life sciences ☐ Behavioural & social sciences ☐ Ecological, evolutionary & environmental sciences

For a reference copy of the document with all sections, see nature.com/documents/nr-reporting-summary-flat.pdf

Life sciences study design

All studies must disclose on these points even when the disclosure is negative.

Sample size	We used 5-12 mice of each genotype for different behavior tests; 8 mice for MRI studies, 8 mice for electrophysiology studies, 5-6 mice for electrophysiology studies, and 2-5 mice for biochemistry and immunostaining analyses. These numbers are comparable to those used in previous publications.
Data exclusions	In fear context discrimination assay, one of the four cameras for a chamber malfunctioned, and gave readings that were abnormally high. We excluded all three animals from this chamber.
Replication	Some experiments are replicated with a entire new cohort. These include fear conditioning, and fear discrimination. All biochemistry experiments, ChIP-seq, RNA-seq and RT-PCR have done at least twice with independent animals.
Randomization	This is not relevant to our study because we are comparing phenotypes between two groups: wildtype vs. Top3b-KO.
Blinding	We were not blinded to genotype during bevavior experiments that were scored automatically by computers. For human scored tests, we were blind to treatment at the time of testing.

Reporting for specific materials, systems and methods

We require information from authors about some types of materials, experimental systems and methods used in many studies. Here, indicate whether each material, system or method listed is relevant to your study. If you are not sure if a list item applies to your research, read the appropriate section before selecting a response.

Materials & experimental systems

n/a	Involved in the study	
☐	☒	Antibodies
☒	☐	Eukaryotic cell lines
☒	☐	Palaeontology
☐	☒	Animals and other organisms
☒	☐	Human research participants
☒	☐	Clinical data

Methods

n/a	Involved in the study	
☐	☒	ChIP-seq
☒	☐	Flow cytometry
☐	☒	MRI-based neuroimaging

Antibodies

Antibodies used	RNA pol II, Biolegend, 664906; Anti-RNA pol II phospho S2 Ab, abcam, ab5095; Anti-RNA pol II Phospho S5 Ab, Abcam, ab5131; Anti-Top3b Ab, Sigma, WH0008940M1; Anti-TDRD3 Ab, Rabbit polyclonial Ab was made against MBP-fused proteins, New England Biolabs; H3K4me1, Abcam, Ab8895; H3K4me2, Abcam, ab32356, H3K4me3, Abcam, ab8580; H3K9me3, Abcam, ab8898; H3K9AC, Abcam, ab4441; H3K27Ac, Abcam, ab4729; H3K27me3, Millipore/Sigma 07-449
Validation	These antibodies have been used in published papers.

Animals and other organisms

Policy information about studies involving animals; ARRIVE guidelines recommended for reporting animal research

Laboratory animals	Mouse on C57bl/6 background
Wild animals	*Provide details on animals observed in or captured in the field; report species, sex and age where possible. Describe how animals were caught and transported and what happened to captive animals after the study (if killed, explain why and describe method; if released, say where and when) OR state that the study did not involve wild animals.*
Field-collected samples	*For laboratory work with field-collected samples, describe all relevant parameters such as housing, maintenance, temperature, photoperiod and end-of-experiment protocol OR state that the study did not involve samples collected from the field.*

Ethics oversight	National Institute on Aging animal care and use committee (ACUC)

Note that full information on the approval of the study protocol must also be provided in the manuscript.

ChIP-seq

Data deposition

☒ Confirm that both raw and final processed data have been deposited in a public database such as GEO.

☒ Confirm that you have deposited or provided access to graph files (e.g. BED files) for the called peaks.

Data access links<br>*May remain private before publication.*	*For "Initial submission" or "Revised version" documents, provide reviewer access links. For your "Final submission" document, provide a link to the deposited data.*
Files in database submission	We have submitted to GEO database a long list of raw and processed data files of ChIP-seq and RNA-seq files. GSE145730.
Genome browser session<br>(e.g. UCSC)	IGV.2.7.2 with mm10 genome.

Methodology

Replicates	For ChIP-seq and RNA-seq, we have done each experiment at least twice using independent animals.
Sequencing depth	about 20 million reads
Antibodies	RNA pol II, Biolegend, 664906; Anti-RNA pol II phospho S2 Ab, abcam, ab5095; Anti-RNA pol II Phospho S5 Ab, Abcam, ab5131; Anti-Top3b Ab, Sigma, WH0008940M1; Anti-TDRD3 Ab, Rabbit polyclonial Ab was made against MBP-fused proteins, New England Biolabs; H3K4me1, Abcam, Ab8895; H3K4me2, Abcam, ab32356, H3K4me3, Abcam, ab8580; H3K9me3, Abcam, ab8898; H3K9AC, Abcam, ab4441; H3K27Ac, Abcam, ab4729; H3K27me3, Millipore/Sigma 07-449
Peak calling parameters	We have used SICER 1.1 to call peaks. The area with significant SICER scores (p-value <=0.05 , SICER score >=200, and false discovery rate <=0.05) are marked as "binding islands".
Data quality	Our ChIP-seq data from wildtype mice are comparable to the published data. For example, the profiles of Pol II, and active gene marks (H3K4me1-3, H3K9Ac, H3K27Ac) are peaked at transcription start sites, similar to the published profiles, indicating high data quality.
Software	SICER 1.1

Magnetic resonance imaging

Experimental design

Design type	Structural neuroimaging study of anesthetized mice
Design specifications	Study of individual mice at 2-3 months of age without stimulus or interventions
Behavioral performance measures	Not applicable: mice were scanned under anesthesia and without stimulation

Acquisition

Imaging type(s)	Structural
Field strength	7.0
Sequence & imaging parameters	A package of 12 contiguous, 1 mm thick coronal slices were scanned with an in-plane field-of-view (FOV) of 30 mm × 20 mm (left-right × anterior-posterior). Diffusion-weighted scans were acquired with 256 × 128 pixels, resulting in a voxel size of 117 × 156 × 1000 microns. A fat-suppressed spin-echo diffusion pulse sequence was used with repetition time TR = 4 s, echo time TE = 30 ms, gradient duration = 7 ms, gradient separation = 14 ms, 2 averages and 17 min total scan time. Inversion-prepared spin-echo scans were acquired with 192 × 256 pixels, resulting in a voxel size of 156 × 78 × 1000 microns. Acquisition parameters were TR = 6 s, TE = 7.5 ms, inversion time TI = 604 ms, 2 averages and 25 min total scan time.
Area of acquisition	The whole brain was scanned in a body-axial, brain-coronal orientation from the olfactory bulb to the brain stem.

Diffusion MRI ☒ Used ☐ Not used

Parameters: One direction: slice (head-foot), b = 200 s/mm2, single shell, no cardiac gating. This was a diffusion-weighted (DWI) scan to measure total brain volume, not a diffusion tensor (DTI) study.

Preprocessing

Preprocessing software	All processing was performed in Bruker ParaVision 5.1. For each mouse, regions of interest (ROI's) outlining the ventricles were manually drawn on those slices of the inversion-prepared scan where these structures were visible. The total number of pixels in these ROI's was calculated and multiplied by the voxel volume to give the volume of the ventricles. Similarly, ROI's outlining the brain were drawn on each slice of the diffusion-weighted scan and total brain volume was calculated by summing the number of pixels in these ROI's and multiplying by the voxel volume.
Normalization	For each mouse, the total ventricle volume was normalized by the total brain volume. The resulting ventricle volume fraction, expressed in percent, was used in all further analysis.
Normalization template	For each mouse, ventricle and whole brain areas in each slice were manually delineated, without registration to any template.
Noise and artifact removal	No motion correction was performed. The mouse's head was firmly fixed using a tooth bar and ear bars so no respiratory gating was needed. The default Gaussian smoothing in Bruker ParaVision's image processing and display (xtip) interface was applied to the images before manual definition of regions of interest.
Volume censoring	Not applicable: total brain and ventricle areas in each slice were delineated manually.

Statistical modeling & inference

Model type and settings	A simple two-tailed T-test was used to compare the percent ventricle volumes of mice in the wild-type and topoisomerase-3-beta knockout groups.
Effect(s) tested	A two-tailed T-test was used to compare ventricle volume, expressed as a percentage of total brain volume, in the two groups of mice. The goal was to test the hypothesis that the topoisomerase-3-beta knockout mice would have larger ventricles than wild type mice; this was suggested by published neuroimaging data from schizophrenic subjects.

Specify type of analysis: ☐ Whole brain ☐ ROI-based ☒ Both

Anatomical location(s)	Anatomical regions (whole brain and ventricles) were manually delineated without any automatic labeling or segmentation software.
Statistic type for inference (See Eklund et al. 2016)	Not applicable: this was a simple comparison of percent ventricle volume in two groups of mice at a single time point and employed a simple two-tailed T-test.
Correction	Not applicable: only a single comparison (percent ventricle volume) was performed.

Models & analysis

n/a	Involved in the study
☒	☐ Functional and/or effective connectivity
☒	☐ Graph analysis
☒	☐ Multivariate modeling or predictive analysis
