# Supplementary material for: Topoisomerase 3β knockout mice show transcriptional and behavioural impairments associated with neurogenesis and synaptic plasticity
Source: Nat Commun. 2020 Jun 19;11:3143. doi: 10.1038/s41467-020-16884-4 (PMC7305123; doi:10.1038/s41467-020-16884-4)
Supplement: Supplementary file 2 — Peer Review File [file 41467_2020_16884_MOESM2_ESM.docx]

Peer Review Information

**Journal:** Nature Communications

**Manuscript title:**
Topoisomerase 3β knockout mice show transcriptional and behavioural impairments associated with neurogenesis and synaptic plasticity

**Corresponding author name(s):** Dr Weidong Wang

**Editorial notes:**

**Transferred manuscripts**

This manuscript has been previously reviewed at another journal that is not operating a transparent peer review scheme. This document only contains reviewer comments and rebuttal letters for versions considered at *Nature Communications*.

**Reviewer comments & decisions:**

**Reviewer comments, first version:**

Reviewers' comments:

Reviewer #1 (Remarks to the Author):

Topoisomerase 3b is a somewhat unique topoisomerase as its been linked to regulation of both DNA and RNA topology. Its loss is compatible with viability (in contrast to Top1 or Top2b) suggesting some selectivity of function outside of widespread topological regulation of general DNA transactions/processes. Importantly, Top3b inactivation has been linked to schizophrenia and cognitive function. The direct association between these processes has derived from population studies where homozygous gene deletion has been found in schizophrenia cohorts and Top3b loss has also been implicated in cognitive impairment. Thus, understanding the role of Top3b in the nervous system is of high importance.

To directly address the neural role of Top3b, the authors have leveraged an available Top3b germline mutant mouse. This KO is viable, although with reduced lifespan and some other reported neural deficiencies including synapse abnormalities and neurogenesis defects. The strategy for analysis is straightforward and involves investigating behavioral aspects of this mouse and also the transcriptional response to fear conditioning. Overall, this is a valuable study that makes an important contribution to the field. They show that the loss of Top3b functionality results in attenuated neural gene expression associated with fear-conditioning. The data is extensive, well-presented and of high quality. Overall, I think this study is a strong contribution to the literature.


Specific comments.

Figure 4 contains key ChIP-seq data that is also used for Figs 6, 7 & 8 (the identification of genes involved in dementia, schizophrenia and neurogenesis). So, essentially the RNA Pol II data is mined for specific genes in an additional 3 figures. Because this data is central (and essential) for providing important functional impact of Top3b loss, it needs to be very carefully explained. This is critical for individuals not conversant with global chromatin IP work. For instance, the authors should ensure a clear explanation regarding the IGV presentation (one sample?) compared with the graphical presentation (4x and 5x samples?) of the Pol II ChIP-seq data (e.g. Fig 4a vs. b, and 4c vs. d). This is also the case for understanding the graphical presentation in Fig 4f (and similar data sets in Fig 8). The results section introducing Figure 4 needs to better explain the rationale for the approach and the analytical methodology being used to generate conclusions.

Overall the data presentation is quite dense, and it might be beneficial to modify some of the presented content to make for an article with increased readability and digestibility. For instance, Figs 1 & 2 could really be combined without losing the message, as it the case for other figures (e.g. 6, 7 & 8 could be combined or condensed to some extent). A more pared-down and streamlined data set would possibly benefit the conclusions being presented.

In looking at basal Pol II occupancy at gene loci in the Top3b brains from Figures 4, 6, 7 & 8, all genes appear to have reduced Pol II signal (Ser5 and Ser2?), suggesting that the Top3b KO has a global reduction of Pol II at TSS and gene bodies- Is this the case? If so, does this affect interpretation of the effect of Top3b loss on specific genes discussed in this study? If this is not the case, then which genes have Pol II occupancy that isn’t different between KO and WT? (house-keeping genes? Some comments/examples should be shown/mentioned etc)

Comment on the between-animal variability in RNA Pol II ChIP, particularly in the fear conditioned examples and (and untreated mutants) and that all 4 WT samples (blue samples) show no variability.

Include the direction of transcription with an arrow in the Refseq genes

The authors need to state exactly what tissue was used. The manuscript says “brain tissue”. Was it the whole brain? Does this include cerebellum?, was it only cortices or isolated hippocampus etc?


Other comments.

Check the bibliography. The first two refs (#1 and #2) are the same. This may impact numbering etc throughout document.


Reviewer #2 (Remarks to the Author):

Over the last years, several members from the Topoisomerase family have been shown to function in gene regulation. In this manuscript, the authors attempt to understand how mutations in Top3b may result in certain neurological disorders. The authors show that Top3b knockout mice exhibit behavioral phenotypes related to psychiatric disorders and cognitive impairment and defective spatial learning and memory. While characterized to some extent, these observations do not significantly advance our existing knowledge on Top3b mutations and their association with neurological disorders. The most interesting and anticipated part would have been a mechanistic dissection of Top3b action in neurons. However, the authors have limited themselves to showing that the mutant mice exhibit impaired global neuronal activity-dependent transcription in response to fear conditioning stress. Given the previous knowledge that by reducing negative supercoiling and resolving R-loop, Top3b promotes transcription, protects against DNA damage and reduces the frequency of chromosomal translocations- the current manuscript does not fill in the significant gaps in our knowledge.

My additional comments are given below:

Introduction:

The authors miss citing a great deal of relevant literature where Topoisomerases have been implicated in gene expression including in the context of the nervous system. They should seriously consider widening the cited literature. The same applies to the known biology of Top3b.

Results:
• General figure comment: values IN columns rather than floating above? Or relocate them to the legend/results section?
• Fig. 1g, three-chamber sociability test: no statistics for WT mouse side vs Top3β-KO mouse side, or WT empty side vs Top3β-KO empty side. The authors state abnormal social interactions in Top3β-KO, but why are they not directly compared to WT? Data was presumably collected at the same time for this experiment, and although the authors focus on mouse vs empty per genotype, there are still 4 groups so perhaps an ANOVA instead of T-test?
• Fig. 1h-k, Fear Conditioning test: Top3β-KO had increased freezing time vs WT in Context test - conducted 24h post-training, without tone/shock stimuli. May infer that memory is not affected in Top3β-KO, as suggested in MWM data?
• Fig. 2b, MWM: authors state “KO mice showed impaired retention of the spatial memory, because they exhibited no significant difference in time exploring in the platform quadrant versus the no-platform quadrants, whereas the WT mice preferred the platform quadrant” BUT, when you look at the values for the Top3β-KO, they spent the longest time in the platform quadrant vs any other platform (21s) – similar to the WT! Similar trends observed between WT and Top3β-KO in this test (WT values per quadrant = 24, 10, 15, 11; Top3β-KO values per quadrant = 21,14, 17, 8). The ** over the WT value shows significance vs what column? The Top3β-KO column of the same quadrant? Or another WT, or Top3β-KO, quadrant?
• Experiments relating to positive symptoms of schizophrenia, like the locomotor activity? Or locomotor activity in response to novelty? Or sensitivity to psychomimetic drugs? Are any additional tests done on negative symptoms? Like the resident intruder assay (altered aggression behavior)? Was decreased nesting behavior observed in Top3β-KO? In terms of cognitive symptoms, was working memory examined through T-maze or 8 arm radial maze tasks? (Powell and Miyakawa 2006) Why do Top3β-KO behave normally in PPI when there are schizophrenia and autism mouse models that behave abnormally, and when Top3β-KO have increased freezing time in FC test?
• Fig. 2d-f, LTP/LTD: The authors state “suggesting that Top3β deficiency does not affect glutamate release from presynaptic terminals.” NMDA receptor activation leads to synaptic strengthening through LTP and weakening through LTD. Were glutamate levels or NMDA/NMDA receptor levels assessed in Top3β-KO?
• Cultured adult SCs from Top3β-KO and WT: how was proliferation assessed (e.g. BrdU incorporation)? Were Top2α levels assessed in these ASCs and the Top3β-KO in general? (Top2α is highly expressed in proliferating cells) What about Top2β? Were pluripotency markers (Nanog, Klf4, etc) affected by Top3β-KO?
• Was Top3β expression examined in ESCs, NPs, Ns, and other organs? Why were neuronal cells not isolated from the Top3β-KO and examined for markers of neurogenesis/synaptic plasticity? What is the morphology of neurons etc like in these mice? Can ESCs from Top3β-KO differentiate? mRNA/protein expression of Top3β? Hippocampal/other brain regions stained for Top3β in WT mice?
• “We observed enlarged lateral ventricles in some Top3β-KO mouse brains in histology studies” – where’s the figure? Supplemental?
• Fig. 4: were the mRNA of the genes themselves (Npas4, Fos, Arc, Erg1) measured? Were total mRNA levels examined in WT vs Top3β-KO as well as RNA Pol II? If so, was there a correlation between total mRNA and Top3β at TSS and other regions (intergenic, etc)? Is FC a robust method of examining gene transcription? Could the expression of these 4 genes be induced pharmacologically rather than by using the FC behavioral test?
• What if Top3β was catalytically inhibited – what effect would this have? Would it mirror Top3β-KO data?
• Fig. 4a, c: Why was TDRD3 ChIP done for Arc and Fos but not Npas4 or Erg1? Was the TDRD3 ChIP and H3K27ac done in WT or Top3β-KO? (same goes for Fig.6 and Fig.7) Where is the corresponding Pol II ChIP at TSS and exons for Npas4 and Erg1? Fig.4a already shows TSS, why is TSS included in Fig. 4c? – either separate TSS from exon or remove Fig. 4a.
• Fig. 4c: H3K27ac is examined (briefly; only for Arc and Fos), but what about methylation? “We detected a peak of TDRD3 (but not Top3β; data not shown) at TSS of these genes” As TDRD3 contains a Tudor domain – methyl-lysine binding motif – does Top3β bind methylated lysines in H3/H4? Topoisomerases are the second most abundant chromatin protein in eukaryotes after histones and Top2β target chromosomal sites contain H3K4me2 – was there a correlation between Top3β enrichment at TSS and H3K4me2 or other chromatin marks? Were the promoters/introns/intergenic regions of these 4 genes examined for such chromatin marks or Top3β enrichment? Perhaps a Top3β peak would have been observed at intergenic/other regions?
• Fig. S5: what is Top3β binding to at these enhancers; Is there increased H3K27ac at these enhancers? “Top3β-TDRD3 complex can interact with RISC to promote heterochromatin formation” – is there a difference in chromatin accessibility between WT and Top3β-KO?
• The authors state Top3β is required for global neuronal activity-dependent transcription. The authors show reduced EP300 in Top3β-KO with FC vs WT with FC (Fig 7d), but was genome-wide correlation analysis performed to examine this? H3K27ac, H3Kme marks, FAIRE, etc.
• Fig. 6a “Top 10 negative MeSH terms for Pol II changes at TSS no Fear” and Fig. 7a “Top 10 negative MeSH terms for Pol II changes at exons with fear”: easier to interpret data comparing TSS vs exons no fear OR comparing TSS vs exons with fear. Perhaps “…TSS fear (S10a)” and “…exons no fear (S9a)” should be included in Fig.6/Fig.7 respectively, rather than left in supplemental?
• Fig. 7 “(f) A graph shows examples of reduced pol II signals (EP300 and DTNBP1), and reduced induction of Pol signals by FC (BDNF and RGS4), in Top3β-KO mice under FC” – how and why were these examples chosen? Were all genes (the other 19 genes from Fig.7d and Fig.7e) also examined in this context?

Discussion:
• Top3β mutations are mentioned throughout manuscript, e.g.: “Our data suggest that Top3β is crucial for normal brain function and mental health; and that disruption of NADT could be one mechanism by which Top3b mutation causes mental and cognitive disorders.” The authors do not present Top3β mutant data here. Re-word to reflect Top3β deletion. Do the authors have data on Top3β mutants and their role in neuronal activity-dependent transcription? Unreasonable to suggest that Top3β mutations disrupt neuronal transcription without data to back it up (either from authors laboratory or referenced from other research groups).
• “Indeed, etoposide, a Top2β poison that induces DSBs by blocking the topoisomerase from rejoining the broken ends, represses far more genes than it activates [15], arguing that Top2β-mediated DSB formation facilitates only a small fraction of genes during NADT” – the number of genes repressed or activated is somewhat irrelevant. Their function however, is. Not fair to dismiss Top2β so readily, given that genes down-regulated in Top2β−/− neurons show a strong enrichment for gene ontology terms associated with “neurogenesis,” whereas genes up-regulated in Top2β−/− neurons were associated with the GO term “cell division” (Tiwari et al. 2012).
Methods:
• “Top3b KO mouse is originally produced at Harvard University by targeted disruption of mouse Top3b in C57BL/6J background [56]. Age-matched male littermates were used in all experiments, whereas female mice were included in some behavior tests”:
Are the Top3β-KO male or female? Specify! Indicate where females were used; were they used because the Top3β-KO in those tests were female? Light-dark box test was developed with male mice (Bourin and Hascoët 2003). Perhaps the females displayed higher anxiety and/or other behaviour differences? Have male vs female been compared in the tests where females were used? The stage of reproductive cycle, where there are fluctuations in both oestrogens (e.g., 17β-oestradiol) and progestins (e.g., progesterone), and/or hormone concentrations would need to be considered for female mice in behavioural tests – especially the Morris water maze where testing was done across multiple days.
• Elevated plus maze: were mice placed in the maze facing the open or closed arm? Differences can arise if mice are placed facing open vs closed arms. Why are the closed arm walls smaller than standard? (2.5cm vs 15.25cm (Walf and Frye 2007)). Has the maze been validated prior to this study? i.e. have mice that have received either anxiolytic or anxiety-causing pharmacologicals been subjects of the maze?
• Anxiety test: was it feasible/possible to carry out heart-rate monitoring on the Top3β-KO mice as a measure of anxiety? And were cortisol/epinephrine levels measured in Top3β-KO mice post-behavioural tests?
• Morris Water Maze: “4 hrs following the last training, mice were placed in water without platform for the first probe trial.” Should this say 24 hrs? and “when the platform was removed for a 60-sec probe trial after the last day of training” – Ref #59 states a probe trial should be done at least 24 hours after last learning trial if they are intended to assess reference memory. Should both probe trials have been more than 24 hours later?
• Three-chamber sociability test: In Ref #60 - “At the end of the first 10 minutes, each mouse was tested in a second 10-minute session to quantitate social preference for a new stranger. A second, unfamiliar mouse was placed in the chamber that had been empty during the first10-minute session.” Why was this not done here?
• MRIs for ventricular enlargement (a feature of schizophrenia) were done at 2-3 months. Is this time point too early? Perhaps the authors would have seen statistical significance if MRIs were performed at 6 months?
• “Newborn neurons” – X hours after birth? P1? P2?
• For “Stereotaxic surgery and viral vector injections”, what age were the mice used? Ref #63 says 5 weeks? Males? Females?
• Similarly, for BrdU injections – age/sex of mice? Also, BrdU was administered i.p. for 3 days to label adult neural SCs in the brain. Could it have been administered i.v. to increase the chances of BrdU crossing BBB? Only 20 positive BrdU cells in WT… poor route of administration? Clearance of BrdU before it got a chance to label adult neural SCs?
• Sample sizes? – Figures 4, 6 (and presumably Figure 7): “The data are from 4 pairs of untreated mice, and 5 pairs of FC-treated mice” – n = 4-5 per group. Is this sufficient? Individual mice in each group show highly variable measurements. What are the n numbers for the behavioral tests – is each point in the graphs in Fig.1 and Fig.2 an individual mouse? Or an individual trial (e.g. 12 trials from 4 mice?)

Inconsistencies in labeling etc:
• “Top3b” instead of “Top3β” throughout the manuscript
• Fig. 4e: the corresponding main text has mislabelled this as 3e - “Among them, 31 (69%) also showed at least 1.5-fold increase (Fig. 3e). In contrast, only 3 of these 31 genes showed a significant increase in Top3β-KO mice (Fig. 3e and S4a)”
• Fig. 5: main text referring to 5d and 5e needs to be clarified – 5d is not mentioned at all, and there’s a discrepancy between numbers in Fig.5e (exons and TSS in blue bars) and corresponding text.
• Fig. 5d: “We also noted that about 2400 genes (10% of total) showed reduced Pol II signals at exons (>1.5-fold, p<0.05) in response to FC in WT mice (Fig. 5d); and this number was only slightly different in Top3β-KO mice (2100; 9%)” – use exact numbers from data: 2373, 2088.
• Fig. 5g: two percentages in the overlapping part of the Venn diagram? Explained in the main text but confusing if looking at figure alone. Re-draw Venn diagram to reflect the specifics of these percentages. “49%” is not referred to in the main text?
• “Furthermore, TDRD3 signals at TSS have a strong negative correlation with reduced Pol II signals in Top3β-KO mice vs. those of wildtype mice under FC (r=-0.67) (Fig. 5i)” – this is mislabelled – should be Fig. 5k.

**Author rebuttal, first version:**

**Reviewers' comments:**

**Reviewer #1 (Remarks to the Author):**

*Topoisomerase 3b is a somewhat unique topoisomerase as its been linked to regulation of both DNA and RNA topology. Its loss is compatible with viability (in contrast to Top1 or Top2b) suggesting some selectivity of function outside of widespread topological regulation of general DNA transactions/processes. Importantly, Top3b inactivation has been linked to schizophrenia and cognitive function. The direct association between these processes has derived from population studies where homozygous gene deletion has been found in schizophrenia cohorts and Top3b loss has also been implicated in cognitive impairment. Thus, understanding the role of Top3b in the nervous system is of high importance.*

*To directly address the neural role of Top3b, the authors have leveraged an available Top3b germline mutant mouse. This KO is viable, although with reduced lifespan and some other reported neural deficiencies including synapse abnormalities and neurogenesis defects. The strategy for analysis is straightforward and involves investigating behavioral aspects of this mouse and also the transcriptional response to fear conditioning. Overall, this is a valuable study that makes an important contribution to the field. They show that the loss of Top3b functionality results in attenuated neural gene expression associated with fear-conditioning. The data is extensive, well-presented and of high quality. Overall, I think this study is a strong contribution to the literature.*

**Response:** We want to thank Reviewer 1 for his/her positive comments.
*Specific comments.*

*Figure 4 contains key ChIP-seq data that is also used for Figs 6, 7 & 8 (the identification of genes involved in dementia, schizophrenia and neurogenesis). So, essentially the RNA Pol II data is mined for specific genes in an additional 3 figures. Because this data is central (and essential) for providing important functional impact of Top3b loss, it needs to be very carefully explained. This is critical for individuals not conversant with global chromatin IP work. For instance, the authors should ensure a clear explanation regarding the IGV presentation (one sample?) compared with the graphical presentation (4x and 5x samples?) of the Pol II ChIP-seq data (e.g. Fig 4a vs. b, and 4c vs. d). This is also the case for understanding the graphical presentation in Fig 4f (and similar data sets in Fig 8). The results section introducing Figure 4 needs to better explain the rationale for the approach and the analytical methodology being used to generate conclusions.*

**Response:** We have added more texts in Results and explain the rationale for the approach and the analytical methods. The reviewer is correct that we used bedgraphs to display ChIP-Seq results for one representative experiment, consisting of a pair of untreated and a pair of Fear-conditioning-treated wildtype or Top3b-KO mice. We also used graphic presentation to show results from 4 pairs of untreated mice (WT and mutant), and 5 sets of fear-conditioning treated mice (WT and mutant). The IGV browser-generated bedgraphs can reveal the location and strength of the Pol II peaks, whereas the bar graphs show quantification of the results, and whether they are statistically significantly different.

1

We added statements in Results that we used representative bedgraphs generated by IGV genome browser, and the quantification of the data from multiple mice.

*Overall the data presentation is quite dense, and it might be beneficial to modify some of the presented content to make for an article with increased readability and digestibility. For instance, Figs 1 & 2 could really be combined without losing the message, as it the case for other figures (e.g. 6, 7 & 8 could be combined or condensed to some extent). A more pared-down and streamlined data set would possibly benefit the conclusions being presented.*

**Response:** As suggested, we have combined Figures 1 and 2 into one figure; and Figure 6, 7, and 8 into two figures. We transferred some data from main figures into Supplemental Figures.

*In looking at basal Pol II occupancy at gene loci in the Top3b brains from Figures 4, 6, 7 & 8, all genes appear to have reduced Pol II signal (Ser5 and Ser2?), suggesting that the Top3b KO has a global reduction of Pol II at TSS and gene bodies- Is this the case? If so, does this affect interpretation of the effect of Top3b loss on specific genes discussed in this study? If this is not the case, then which genes have Pol II occupancy that isn’t different between KO and WT? (house-keeping genes? Some comments/examples should be shown/mentioned etc)*

**Response:** Based on our statistical analysis (ANOVA) from multiple mice, Top3b-KO does not have significant reduction of pol II, pol II-ser2 or pol II-ser5 at TSS and gene body for most of the genes (See Figure 4f for the number of genes with pol II changes). The genes with reduced pol II signals represent about 15% and 26% of total genes using data at exons and TSS, respectively, under fear conditioning. About 84% and 73% of genes showed no statistical difference using data at exons and TSS, respectively; whereas about 0.1 % showed an increase. To make this point clear to the reviewers and the readers, we have added in Results more description of the data from Fig. 4f, to indicate that Top3b is not needed for transcription of majority of genes.

We should mention that the representative bedgraphs are based on only one set of experiments, and thus do not represent results from all experiments. This is the reason why we displayed bargraphs to summarize data from multiple experiments so that readers can note the variability in our ChIP-seq data, and which difference is statistically significant (see examples in Fig. 3b, 3c; Fig. S5b,c).

In addition, we found an error in our statistical analyses of pol II ChIP-seq data, so that we have previously over-estimated the number of genes that have significant difference between Top3b-KO and wildtype mice. We have corrected the mistake in the revised version. The new analysis showed that the number of genes with significant difference are fewer than the previous version.

As requested by the reviewer, we also added one Supplemental Figure (Fig. S9d) to show that one class of house-keeping genes—actin and actin-related proteins, showed reduction in only 4 of 30 genes (about 13%) in Top3b-KO mice; whereas most of them showed no statistical difference. Moreover, we also included a statement in Results indicating that c-myc gene is not significantly affected in Top3b-KO mice (Fig. S8a,b ). One reason to use c-myc as an example is that a previous study (Yang et al. Mol. Cell 2014) proposed that Top3b may be recruited by TDRD3 to this promoter to reduce R-loops and increase its transcription (see our response to reviewer 2). However, our data do not support this hypothesis.

2

*Comment on the between-animal variability in RNA Pol II ChIP, particularly in the fear conditioned examples and (and untreated mutants) and that all 4 WT samples (blue samples) show no variability.*

**Response:** We have observed between-animal variability in Pol II ChIP signals, but do not have a good explanation for the underlying mechanism. We also noticed that that there is also between-animal variability for both WT and Top3b-KO mice in the fear behaviors (freezing time) in response to fear conditioning treatment (between 20-80 sec). This variation has also been observed by many other studies. One possibility is that the hormonal levels of the animals are different.

Regarding the no variability of 4 untreated WT samples (blue), this is due to data normalization. To compare ChIP-seq data from different sets of experiments, we performed data normalization by using data from untreated WT mice in each experiment as the standard, which is set as “1”, and thus invariable. Data from all other mice in the same data set are divided by the data from untreated WT mice. We added this explanation to the Figure Legend.

*Include the direction of transcription with an arrow in the Refseq genes.*
**Response: W**e have added arrows in the Refseq genes as requested.

*The authors need to state exactly what tissue was used. The manuscript says “brain tissue”. Was it the whole brain? Does this include cerebellum?, was it only cortices or isolated hippocampus etc?*

**Response:** We have indicated in the Results that we used mouse whole brain for various analysis.

We have since analyzed several individual tissues, including isolated hippocampus and cortex, and have obtained consistent results, which will be published in future papers.

*Other comments.*

*Check the bibliography. The first two refs (#1 and #2) are the same. This may impact numbering etc throughout document.*

**Response:** We have corrected this mistake.
*Reviewer #2 (Remarks to the Author):*

*Over the last years, several members from the Topoisomerase family have been shown to function in gene regulation. In this manuscript, the authors attempt to understand how mutations in Top3b may result in certain neurological disorders. The authors show that Top3b knockout mice exhibit behavioral phenotypes related to psychiatric disorders and cognitive impairment and defective spatial learning and memory. While characterized to some extent, these observations do not significantly advance our existing knowledge on Top3b mutations and their association with neurological disorders. The most interesting and anticipated part would have been a mechanistic dissection of Top3b action in neurons. However, the authors have limited themselves to showing that the mutant mice exhibit impaired global neuronal activity-dependent transcription in response to fear conditioning stress. Given the previous*

3

*knowledge that by reducing negative supercoiling and resolving R-loop, Top3b promotes*

*transcription, protects against DNA damage and reduces the frequency of chromosomal translocations-*

*the current manuscript does not fill in the significant gaps in our knowledge.*

**Response:** We respectively disagree with the reviewer on his/her assessment on whether our manuscript represents a significant advancement of knowledge on Top3b mutations and their association with neurological disorders. The reviewer may have made his/her assessment based on “*the previous knowledge that by reducing negative supercoiling and resolving R-loop, Top3b promotes transcription, protects against DNA damage and reduces the frequency of chromosomal translocations”.* We think this previous knowledge is from a single paper by Yang et al., Mol Cell, 2014, which was cited in our Introduction. The paper actually showed only that Top3b recombinant protein can relax negative supercoiled DNA and reduces R-loops *in vitro*. However, the paper lacks the crucial evidence: it has no data to show that Top3b-Knockout or knockdown cells display increased R-loops or reduced transcription. The absence of this crucial evidence is intriguing, because these authors did present data that TDRD3-Knockdown MCF cells have increased R-loops at the c-Myc locus, and about 57% reduction of c-Myc mRNA level; and TDRD3-null mice have increased c-myc/Igh translocation in B cells. Because this **previous paper lacks evidence from Top3b-Knockdown or knockout cells**, the proposal that TDRD3 recruits Top3b to reduce R-loops and stimulate transcription is still a hypothesis, waiting to be approved or disapproved by studies using Top3b-Knockout or knockdown cells. As far as we can tell, no such evidence has been reported from these authors after their original publication 5 years ago.

In our hands, transcription of c-myc gene, by either pol II ChIP-seq or RNA-seq, is not significantly reduced in Top3b-Knockout mouse brains without or with fear conditioning treatment, which is in contrast to many other neuronal early response genes that show reduction (Fig. S8a,b; and RNA-seq data not shown). We also failed to detect reduced c-Myc transcription in Top3b-knockout human cancer cell lines HCT116 and K562 (data not shown). These negative data do not support the hypothesis by Yang and colleagues. We have discussed the discrepancy with an expert in the c-myc field, Dr. David Levens from NCI. We were told that formation of R-loops is not a major issue at c-Myc gene; and Yang’s hypothesis could be wrong, which is consistent with our findings.

To make the readers understand better the previous evidence on Top3b-TDRD3 complex in transcription, we re-wrote this part of the Introduction to make clear to readers that reduced transcription and increased R-loops have been observed only in TDRD3, but not Top3b, knockdown cells, so that the proposal by Yang and colleagues is a hypothesis. We also pointed out in our Results that c-myc transcription is not significantly reduced in our system, unlike other early response genes, which is not consistent with the proposal by Yang and colleagues.

We would like to point out that the most important conclusion of our manuscript is that it is **the first and only study** that uses a mammalian model to demonstrate that Top3b gene inactivation can lead to behavior abnormality and neurological defects observed in human and animal models of schizophrenia, autism and cognitive dysfunction. Without these positive data from our study, Top3b mutations observed by human GWAS studies are just “associations” with the neurological disorders. But with our positive data, Top3b mutations should be considered as “causal” to the disorders. Thus, our findings should provide the crucial evidence that fills the gap in understanding of the relationship between Top3b mutations and neurological disorders.

4

In addition, our study is **the first and only study** that shows Top3b inactivation can lead to disruption in hippocampal synaptic transmission and adult neurogenesis, which have been observed in human or animal models of mental disorders and cognitive dysfunction. Our findings thus suggest that defective hippocampal development and function could be a cellular mechanism that causes the mental and cognitive dysfunction in human. In our opinion, the findings should fill another important gap of our knowledge between Top3b mutation and neurological disorders.

Moreover, our study is **the first and only study** that shows Top3b-knockout animals exhibit deficient neuronal activity-dependent transcription of genes of neuronal early response, as well as genes that are known to be associated with various neurological disorders. We demonstrate that Top3b-TDRD3 can directly bind to a large number of these genes in response to neuronal activation, and stimulate transcription as measured by pol II ChIP-seq, as well as RNA-seq (new data). These findings provide a molecular mechanism of Top3b-TDRD3 complex, and should therefore represent another major advance for our understanding of how Top3b mutations can cause neurological disorders.

*My additional comments are given below:
Introduction:*

*The authors miss citing a great deal of relevant literature where Topoisomerases have been implicated in gene expression including in the context of the nervous system. They should seriously consider widening the cited literature. The same applies to the known biology of Top3b.*

**Response:** We have widened our literature search and found many relevant papers on the subject. One of them is an excellent article by Tiwari and colleagues (PNAS 2012), which dissected molecular mechanism of how Top2b regulates neuronal transcription and survival using Top2b-KO ESC cells. We performed multiple assays and made similar figures as described in this paper, and found that Top3b-TDRD3 complex resembles Top2b in several regards, including enrichment at promoters, strong correlation with pol II and RNA-seq signals, and preferentially binding to genes with active but not repressive chromatin marks (See new Figure 5). We even made a correlation analyses and found a good positive correlation and co-clustering between genes bound by TDRD3 and by Top2b, using the list from Tiwari’s paper. The new figure should add novel mechanism of Top3b-TDRD3 complex, and we thank the reviewer for his/her suggestion.

There are also other relevant publications. To save space, we cited a recent review article by Peter McKinnon (Nat. Rev. Neurosci., 2016), so that readers can find the relevant literature there.

*Results:*

- *General figure comment: values IN columns rather than floating above? Or relocate them to the legend/results section?*

**Response:** We have moved the values into the data bars.

- *Fig. 1g, three-chamber sociability test: no statistics for WT mouse side vs Top38-KO mouse side, or WT empty side vs Top38-KO empty side. The authors state abnormal social interactions in Top38-KO, but why are they not directly compared to WT? Data was presumably collected at the same time for this*

5

*experiment, and although the authors focus on mouse vs empty per genotype, there are still 4 groups so perhaps an ANOVA instead of T-test?*

**Response:** Our three-chamber sociability test was on a smaller cohort of mice (n=7 per group). To verify the findings and perform analysis as suggested by the reviewer, we redid this experiment with a larger cohort (n=12/group) and performed an additional social experiment involving free exploration with a juvenile mouse. Social data presented in the paper is now solely from the new cohort. In three-chamber sociability we found that direct comparison of results from groups gave significant differences in latency to first investigation, with knockout mice being slower to initiate investigation. Groups did not differ in overall time investigating mouse or time in mouse side (data not shown). Unexpectedly, in free social exploration the knockout mice showed increased total social interaction with a juvenile mouse at the beginning of the session. As this overall pattern of results doesn’t illustrate a simple phenotype of being more or less “social”, we now refer to the knockout social phenotype as simply “abnormal.”

- *Fig. 1h-k, Fear Conditioning test: Top38-KO had increased freezing time vs WT in Context test - conducted 24h post-training, without tone/shock stimuli. May infer that memory is not affected in Top38-KO, as suggested in MWM data?*

**Response:** We understand that the reviewer may not be convinced by our previous presentation of Morris water maze data, which showed defective learning and memory in Top3b-KO mice. We have now re-analyzed the data, and presented a new figure showing that in the probe test, the average distance explored by Top3b-KO mice with respect to the position of the hidden platform is significantly longer than that by wildtype mice (Fig. 1l). The data indicate that Top3b-KO mice are defective in spatial memory, which should depend on the hippocampus.

We have also provided additional evidence for impairments in hippocampal-dependent memory using the context discrimination test (Frankland et al., Behav. Neurosci., 112:863, 1998). This is a new test with new mice and represents a significant addition of new data to the revised manuscript. The results from this new experiment are clear (Fig. 1i-j): In the training day, KO mice froze in a “context B” that was not paired with electric shocks, whereas wild-type mice did not; indicating that the KO mice are deficient in discriminating two different spatial contexts (A vs. B). Moreover, KO mice have reduced

discrimination index on the test day. Importantly, this test compares within-subject data of mice to normalize for any non-specific differences in freezing behavior. Thus, the data are consistent with our MWM data that the Top3b-KO mice are defective in hippocampal dependent memory.

Our context discrimination test also showed that Top3b-KO mice have increased freezing time in both context A and B on the test day (Fig. S1C), which provide independent evidence for our findings from the fear conditioning test that these mice show heightened conditional fear.

It should be mentioned that some mouse models of neurological disorders exhibit the same phenotype as Top3b-KO mice: with enhanced fear memory but reduced spatial memory. One example is the 3X Tg-AD mouse model of Alzheimer’s Disease (Stover et al., Behav Brain Res., 2015,289:29-38). The divergence in responding to fear conditioning versus other memory tasks demonstrates that these behaviors are mediated by different brain regions / circuits. To help readers understand that there is precedence for this divergence of memory behaviors in another mouse model of neurological disorders,

6

we added a statement in the Discussion to describe this similarity between Top3b-KO and 3xTg-AD mouse model,

- *Fig. 2b, MWM: authors state “KO mice showed impaired retention of the spatial memory, because they exhibited no significant difference in time exploring in the platform quadrant versus the no-platform quadrants, whereas the WT mice preferred the platform quadrant” BUT, when you look at the values for the Top3β-KO, they spent the longest time in the platform quadrant vs any other platform (21s) – similar to the WT! Similar trends observed between WT and Top3β-KO in this test (WT values per quadrant = 24, 10, 15, 11; Top3β-KO values per quadrant = 21,14, 17, 8). The ** over the WT value shows significance vs what column? The Top3β-KO column of the same quadrant? Or another WT, or Top3β-KO, quadrant?*

**Response:** Upon closer investigation of the MWM data, we agree with the reviewer that the results from wildtype and Top3b-KO mice converged at the 24-hour time point, which appeared due in part to wild-type mice losing preference for the quadrant that contained the platform – as would be normally expected during extinction learning. We therefore re-analyzed our data by focusing on the 4-hour time point of the probe test, during which the group differences between wildtype and Top3b-KO mice were most pronounced. Here we report the data from the 4-hour time point using the “distance from platform” metric, which was found to be the most sensitive measure for the MWM test in a study of 1,600 probe test trials (Maei et al., Front Integr Neurosci., 3:33, 2009). In our measurement (new Fig. 1l), the average distance explored by Top3b-KO mice relative to the position of the hidden platform were significantly longer than that by wildtype mice, indicating that Top3b-KO mice have impaired spatial memory. The finding is consistent with data from the invisible platform training sessions in the Morris water maze (Fig. 1k), as well as with the data from the new context discrimination behavioral test (Fig. 1i-j). They also in agreement with the electrophysiological and biochemical data we present in this manuscript indicating impairment in hippocampal function.

- *Experiments relating to positive symptoms of schizophrenia, like the locomotor activity? Or locomotor activity in response to novelty? Or sensitivity to psychomimetic drugs? Are any additional tests done on negative symptoms? Like the resident intruder assay (altered aggression behavior)? Was decreased nesting behavior observed in Top3β-KO? In terms of cognitive symptoms, was working memory examined through T-maze or 8 arm radial maze tasks? (Powell and Miyakawa 2006) Why do Top3β-KO behave normally in PPI when there are schizophrenia and autism mouse models that behave abnormally, and when Top3β-KO have increased freezing time in FC test?*

**Response:** We have performed additional behavior tests to address reviewer’s comments.

We have already shown that Top3b-KO mice do not show different locomotor activity in open field test without any treatment (Supplemental Figure S2a-d after reorganization of figures).

We have tested locomotor activity in mice retreated with a psychomimetric drug (MK-801), as previous studies have shown that some schizophrenia mouse models exhibit enhanced locomotor activity in response to this drug (Ayhan et al., Mol. Psychiatry, 16:293, 2011; Ibi et al., Behav. Brain Res. 2010). The results show a strong trend of increased locomotor activity for Top3b-KO mice similar to other schizophrenia mice; but the difference with that of the wildtype mice does not reach statistical significance except for one time point out of 18 total time points. We deem these results ambiguous,

7

which are not strongly for or against the notion if Top3b-KO mouse is a schizophrenia mouse model. We decided not to include these confusing data in the manuscript, but show them to the reviewers as Appendix Figure 1. We plan to repeat this experiment in future studies using mice that have not been through various behavior tests, to see if we can observe significant difference.

We did not perform resident intruder assay, because when we performed reciprocal social interaction test, in which the test mouse has free access to the target mouse (instead of target mouse being restricted in a protective cage, as in resident intrude test), we did not observe any instances of aggressive behavior by Top3b-KO mouse toward the target mouse.

We have performed nest building test as well as marble burying test. However, the results are negative or inconclusive. We decide not include them in the manuscript.

We did not perform T-maze and radial arm maze tests to test the working memory. The reason is that in this manuscript, we plan to focus on Top3b-KO behavior phenotypes that can be linked to defects in neuronal activity dependent transcription, which usually takes long time to show its effects on behaviors (it takes time to produce proteins, which affects behaviors directly). Working memory errors in T-maze and radial arm maze tests have very short time course (seconds to minutes), so that they are less likely to be affected by reduced neuronal activity dependent transcription in Top3b-KO mice.

On the other hand, we focused more on hippocampus-dependent cognitive function, by performing context discrimination test. This test is well established to be highly sensitive to hippocampal function— it is impaired following lesion of hippocampus (Frankland, 2998), and enhanced following treatments that stimulate hippocampal neurogenesis (Wu, Luna and Hen, Frontiers Sys. Neurosci., 2015). The results are positive and included in Figure 1, which provides strong evidence that Top3b-KO mice have impaired hippocampus-dependent cognitive function. Together with MWM test, we have now shown with **two** different behavioral tests in independent cohorts of animals that Top3b-KO mice have impaired spatial reference memory. There are indeed other cognitive functions mediated by the hippocampus that could possibly be impaired in these top3b-KO mice: working memory, memory for temporal sequences, and reversal learning. These tasks may require both overlapping and non-overlapping hippocampal circuitry, which is not well-established. In our opinion, understanding all hippocampal dependent functions and their underlying circuitry is far beyond the scope of this work.

Regarding why do Top3b-KO mice behave normally in PPI test, unlike other schizophrenia and animal models. We do not have a clear answer, but have a hypothesis based on our pol II ChIP-data that Top3b mutation can reduce neuronal activity-dependent transcription of thousands of genes. These genes include not only those important for schizophrenia and autism, but also other neurological disorders, such as dementia and Alzheimer’s disease. Therefore, we think Top3b-KO mouse is not a pure schizophrenia or autism mouse model, but has phenotype overlapping with those from other disorders. The results are actually consistent with human genetics data, which show that Top3b mutations have been linked to more than one neurological disorders, including schizophrenia, autism, epilepsy, intellectual disability, and cognitive impairment. We have added our hypothesis in the Discussion, and also de-emphasized the resemblance of Top3b-KO mice to schizophrenia. We stated “Overall, these data argue that Top3L-KO mouse is not a simple schizophrenia or autism model, but rather, shares features with several different psychiatric and cognitive disorders. This fits well with human genetic data suggesting that Top3b mutation carriers have a spectrum of behavioral alterations related to multiple psychiatric disorders. It is also consistent with our pol II ChIP-seq data that Top3b deletion disrupts NADT of many genes, which include those important for not only schizophrenia, but also other neurological disorders such as dementia..”

8

- *Fig. 2d-f, LTP/LTD: The authors state “suggesting that Top38 deficiency does not affect glutamate release from presynaptic terminals.” NMDA receptor activation leads to synaptic strengthening through LTP and weakening through LTD. Were glutamate levels or NMDA/NMDA receptor levels assessed in Top38-KO?*

**Response:** We did not measure the glutamate levels or NMDA/NMDA receptor ratios. However, our field recordings (Fig. 1m) showed the basal synaptic transmission was not altered at lower stimulation intensity, so that it is unlikely that one can detect significant difference in basal glutamate levels between control and KO mice. Because this paper has already presented several lines of evidence that Top3b-KO mice are defective in synaptic transmission and plasticity (Figure 1m-o), and the focus of this paper is not on the mechanism of synaptic dysfunction of Top3b-KO mice, we hope to study these issues in future experiments.

- *Cultured adult SCs from Top38-KO and WT: how was proliferation assessed (e.g. BrdU incorporation)? Were Top2α levels assessed in these ASCs and the Top38-KO in general? (Top2α is highly expressed in proliferating cells) What about Top28? Were pluripotency markers (Nanog, Klf4, etc) affected by Top38-KO?*

**Response:** We assessed the proliferation of the adult stem cells in cell culture by examining whether they are doubling in numbers after we culture them in tissue culture dishes. Those derived from Top3b-KO mice do not double and eventually die, in contrast to those from wildtype mice which increase their numbers over time. We have not analyzed BrdU or other markers, as we have already performed BrdU-labeling and GFP-labeling assays under in vivo conditions, which should be more physiological and thus more convincing than in vitro cultured conditions.

We have examined our RNA-seq data from Top3b-KO mouse whole brains; hippocampus, cortex, and amygdala regions of the brain. We did not detect consistent reduction of Top2a, Top2b, Nanog, Klf4, c-myc, oct4, and sox-2. We have also analyzed our unpublished RNA-seq data from Top3b-KO HCT116 human cells, and observed significant reduction of Klf4 mRNA. Because this study is on Top3b-KO mouse models, we will pursue this lead in future publications.

We should mention that we have examined 250 genes important for neurogenesis, and found some of them show significant reduction of Pol II signals (Fig. 7e), or significant reduced induction of pol II signals (Fig. 7f), in Top3b-KO mouse. We hope to follow up these leads in future studies.

- *Was Top38 expression examined in ESCs, NPs, Ns, and other organs? Why were neuronal cells not isolated from the Top38-KO and examined for markers of neurogenesis/synaptic plasticity? What is the morphology of neurons etc like in these mice? Can ESCs from Top38-KO differentiate? mRNA/protein expression of Top38? Hippocampal/other brain regions stained for Top38 in WT mice?*

**Response:** We have not started examining the roles of Top3b in ESCs. The reason is that we want to first find out if Top3b inactivation in mouse can lead to behavior and neurological abnormalities observed in human psychiatric and cognitive disorders, as suggested by human genetic studies. This is the key issue regarding the biological function of Top3b, and is the focus of this study. We can examine ESCs and other specific tissues and cells in future studies after completing this paper.

9

We have reported in our early publication that cultured primary neuronal cells from Top3b-KO mice have reduced synapse formation, based on staining of synaptic markers (Xu et al., Nat. Neurosci., 2013). We have now included a sentence in the Introduction indicating Top3b-KO mice have abnormal synapse formation in their cultured primary neurons.

The brain Atlas project ([mouse.brain-map.org](http://mouse.brain-map.org)) has reported that Top3b mRNA is highly expressed in hippocampus, cortical subplate, and olfactory area by in situ hybridization. We have added citation of their findings to the text in Results.

- *“We observed enlarged lateral ventricles in some Top38-KO mouse brains in histology studies” – where’s the figure? Supplemental?*

**Response:** We made the visual observation after dissection of the brains that lateral ventricles appear to be enlarged. However, we did not document this difference, because we worry that the size of the lateral ventricles may be altered by dissection, which could vary between different mice. We therefore used MRI to obtain image of live animals to determine if our visual observation is correct or not. The MRI data are included in Supplemental figure (Fig. S3b-c). To avoid confusion, we have removed the sentence that confused the reviewer.

- *Fig. 4: were the mRNA of the genes themselves (Npas4, Fos, Arc, Erg1) measured? Were total mRNA levels examined in WT vs Top38-KO as well as RNA Pol II? If so, was there a correlation between total mRNA and Top38 at TSS and other regions (intergenic, etc)? Is FC a robust method of examining gene transcription? Could the expression of these 4 genes be induced pharmacologically rather than by using the FC behavioral test?*

**Response:** To measure the mRNA levels, we performed total RNA-seq as well RT-qPCR, and added the new data to the paper. RNA-seq data (new Fig. 3d, e) show that the above 4 neuronal early response (NER) genes, Npas4, Fos, Arc, and Egr1, are induced by fear conditioning treatment in wildtype but not Top3b-KO mice. Similarly, RT-qPCR results show significant difference (p<0.05) in the induction between wildtype and Top3b-KO for three of the 4 genes: Arc, Egr1 and Npas4. For the 4^th^ gene, Fos, the difference is very close to be significant (p=0.055) (Fig. S6a-d). Overall, these data are consistent with the Pol II ChIP-seq data that neuronal activity dependent transcription of NER genes are defective in Top3b-KO mice.

We also extended our analysis of RNA-seq data to all 45 NER genes. The results show that significant induction of 12 NER genes (including Npas4, Fos, Arc and Egr1), in wildtype, but none in Top3b-KO mice, by fear conditioning (Fig. 3f, g, h). The data are very similar to the pol II ChIP-seq results, which show significant induction of 28 and 21 of NER genes based on signals at TSS and exons, respectively, in wildtype mice; but none in Top3b-KO mice, by fear conditioning (Fig. 3f-g). Again, the data support the conclusion that Top3b is needed for neuronal activity-dependent transcription.

As requested, we have added a graph describing correlation between TDRD3 ChIP-seq and total RNA-seq data. The correlation coefficient between TDRD3 signals at TSS and RNA-seq is 0.7, which is strong; that between TDRD3 at exons and RNA-seq is 0.6, which is also positive (new Fig. 5e). These numbers are higher or comparable to the correlation coefficient observed between Top2b and RNA levels (0.6)(Tiwari

10

et al., PNAS 2012), suggesting that Top3b-TDRD3 complex may act similarly as Top2b to enhance mRNA expression. We have indicated the similarity between Top3b-TDRD3 and Top2b, and proposed that the two topoisomerases may work together to enhance transcription of many genes in neurons in the manuscript.

Both our pol II ChIP-seq and RNA-seq showed FC is a robust method to induce neuronal activity dependent transcription (Fig. 4a-b; Fig. 3). Our data are consistent with the paper from Tsai’s group (Madabhushi et al., Cell, 2015), which also used FC to induce neuronal activity dependent transcription, including Top2b-mediated double strand breaks at promoters.

In this study, we only used FC, but not other treatment, to induce neuronal activity-dependent transcription. We have explained the rational of using FC in Results. One, FC has been shown to induce Top2b-mediated DSBs, indicating generation of strong topological stress under this condition, which may depend on not only Top2b, but also other topoisomerases, to solve. Two, Top3b-KO mice display abnormal behaviors in FC behavior tests, suggesting that they may have abnormal transcription under this condition. The fact that we were able to identify a crucial role of Top3b in neuronal activity dependent transcription under FC validates our strategy. We think that it is certainly worthwhile to try other approaches to induce neuronal activation. However, these other approaches may or may not induce enough topological stress to reveal a role of Top3b. The results obtained, either positive or negative, may not add too much to the conclusion of this paper. I hope that we can address this question in future studies.

- *What if Top38 was catalytically inhibited – what effect would this have? Would it mirror Top38-KO data?*

**Response:** This experiment is currently not feasible in animals. Unlike Top1 and Top2, there are currently no good inhibitors for Top3a or Top3b. We and our colleagues have tried to develop one, but the KD is still too high to be used in cells. We are still in the process of finding one (Ranjan et al., J. Med Chem., 2017), and hope to address this issue in future.

- *Fig. 4a, c: Why was TDRD3 ChIP done for Arc and Fos but not Npas4 or Erg1? Was the TDRD3 ChIP and H3K27ac done in WT or Top38-KO? (same goes for Fig.6 and Fig.7) Where is the corresponding Pol II ChIP at TSS and exons for Npas4 and Erg1? Fig.4a already shows TSS, why is TSS included in Fig. 4c? – either separate TSS from exon or remove Fig. 4a.*

**Response:** We have done genome-wide TDRD3 ChIP-seq, which covers all genes. We have previously showed two genes (Arc and Fos) in the main figure, and two genes (Egr1 and Npas4) in Supplemental Figure. The reason is to save space for the main figure. To avoid confusion, we have now showed exon bedgraphs for all 4 genes in the main figure (Fig. 3a); and put the TSS bedgraphs of all 4 genes in Supplemental Figure (Fig. S4a).

We have done TDRD3 ChIP-seq from both wildtype and Top3b-KO mice, and have now included the representative bedgraph data for Arc, Fos, Egr1 and Npas4, in a Supplemental Figure (Fig. S4b). TDRD3 signals are reduced in Top3b-KO mice under fear conditioning, which is expected because TDRD3

11

protein stability is reduced in the absence of Top3b. This experiment can serve as a control to show that TDRD3 ChIP signals detected in wildtype mouse brains are specific.

We have done H3K27Ac in both wildtype and Top3b-KO mice, and included most of the new analysis in a Supplemental Figure (Fig. S13). In our previous version, we only showed H3K27ac signals from wildtype mice as a marker in bedgraphs for transcriptionally active genes, including their TSS, exons and enhancers. In the revised version, we showed more analysis, including H3K27Ac signals from both wildtype and Top3b-KO mice for specific genes as well as for all genes, without or with treatment of fear conditioning. The reason is that H3K27Ac has been shown to be induced by neuronal activity in cultured neurons (Malik et al., Nat. Neurosci., 2010). H3K27Ac is also a substrate of p300 histone acetyltransferase, which was found to have reduced pol II signals in Top3b-KO mice. Thus, one possibility is that Top3b inactivation may reduce neuronal activity dependent transcription by decreasing transcription of p300, leading to reduced H3K27Ac, as well as lower transcription. Our results show that Top3b inactivation does not affect H3K27Ac levels at genome-wide scales, or at most of the neuronal early response genes. However, we observed modest reduction of H3K27Ac at TSS or enhancers of some specific NER genes, Fos and Fosb. These data argue that the major effect of Top3b is not H3K27Ac acetylation, although Top3b-TDRD3 complex could play a role in H3K27 acetylation at some specific loci.

As requested, we have moved the original Figure 4a (TSS) to Supplemental Figure (Fig. S4a). This figure shows difference of signals at TSS for Arc, Fos, Egr1 and Npas4.

- *Fig. 4c: H3K27ac is examined (briefly; only for Arc and Fos), but what about methylation? “We detected a peak of TDRD3 (but not Top38; data not shown) at TSS of these genes” As TDRD3 contains a Tudor domain – methyl-lysine binding motif – does Top38 bind methylated lysines in H3/H4? Topoisomerases are the second most abundant chromatin protein in eukaryotes after histones and Top28 target chromosomal sites contain H3K4me2 – was there a correlation between Top38 enrichment at TSS and H3K4me2 or other chromatin marks? Were the promoters/introns/intergenic regions of these 4 genes examined for such chromatin marks or Top38 enrichment? Perhaps a Top38 peak would have been observed at intergenic/other regions?*

**Response:** We have now presented more analyses of H3K27ac (see our response above for Fig. 4a,c). In addition, we have performed ChIP-seq for 3 active methylation markers, H3K4me1, H3K4me2, and H3K4me3, as well as two repressive methylation markers, in both wildtype and Top3b-KO mice. We found that these markers are not significantly altered in Top3b-KO mice for specific NER genes or genome wide scale. To save space, we only showed one representative graph for genes near Arc locus from wildtype mice (Fig. 5g). We also showed a graph of correlation coefficients between signals of these marks and TDRD3 ChIP signals (Fig. 5f), which indicate strong correlation between active methylation gene marks and TDRD3, but no correlation between repressive methylation marks and TDRD3. Moreover, we added a heatmap show co-clustering of TDRD3 bound genes with active but not repressive chromatin marks (Fig. 5h).

The reviewer’s comment on “TDRD3 contains a Tudor domain-methyl-lysine binding motif-does Top3b bind methylated lysines in H3/H4?”. We believe that lysine should be Arginine here. There is extensive literature reporting that Tudor domain of TDRD3 is a methyl-arginine (not lysine) binding motif, which enable TDRD3 to bind arginine methylated histone tails (Yang et al., Mol Cell. 2010, 2014; Liu et al., Plos One, 2012), CTD domains of pol II (Sims et al., Science, 2011), USP9X (Narayanan et al., Cell Dis. 2017),

12

and Med12 (Cheng et al., Lfe Sci Alliance, 2018). We have examined whether TDRD3 peaks co-localizes with the peak of H4R3me2a, which has been proposed to be the primary mark that recruits Top3b-TDRD3 complex to c-Myc promoter (Yang et al., Mol. Cell, 2014). However, our ChIP-seq using two different H4R3me2a antibodies failed to show enrichment of this mark at c-Myc promoter, or other neuronal early response gene promoters, or any promoters, without or with fear conditioning (data not shown). We also failed to find any published ChIP-seq data for H4R3me2a. Thus, our data do not support the notion that Top3b-TDRD3 binds methylated H4R3me2a. Rather, the strong correlation between TDRD3 and pol II signals at TSS and exons favors the hypothesis that Top3b-TDRD3 is recruited by its interaction with methylated CTD of pol II, so that it can help to solve topological problems generated by the polymerase during transcription. We have mentioned our negative data on H4R3me2a briefly in Results, and also described our favored hypothesis on Top3b-TDRD3-pol II interaction in Discussion.

As requested, we have performed ChIP-seq using antibodies against not only H3K4me2, but also H3K4me3 and H3K4me1. We observed strong positive correlation at TSS and exons between TDRD3 signals and H3K4me2, H3K4me3, and somewhat weaker correlation with H3K4me1 (which is an enhancer mark). The data suggest similar mechanisms between Top3b-TDRD3 and Top2b. We included the data in New Figure 5.

As requested, we have examined promoters, introns, and intergenic regions, for different methylation and acetylation marks; and showed a representative bedgraph of Arc genomic locus for Fig. 5g. The reviewer is correct: there are clear TDRD3 peaks in intergenic regions, which represent enhancers because they colocalize with Pol II, H3K4me2, H3K27ac, or other active marks; and some of them also colocalize with the published neuronal activity regulated enhancers by Greenberg and colleagues (Kim et al., 2010). To further show binding of TDRD3 in different genes and intergenic regions, we also showed TDRD3 signals for the 4 genes in both wildtype and Top3b-KO mice (Fig. S4b); and TDRD3 signals with pol II signals at 2 enhancers of Fos and Npas4 (Fig. S7). These data clearly show localization of TDRD3 at enhancers in intergenic regions. We have indicated that TDRD3 and pol II co-bind several neuronal activity-regulated enhancers in intergenic regions.

We added a new figure showing H3K27Ac mark in both wildtype and Top3b-KO mice (Fig. S13). We did not include other marks from Top3b-KO mice, because they do not show consistent difference compared to the wildtype. But if the reviewers want us include more data for a specific mark, we can include them.

- *Fig. S5: what is Top38 binding to at these enhancers; Is there increased H3K27ac at these enhancers? “Top38-TDRD3 complex can interact with RISC to promote heterochromatin formation” – is there a difference in chromatin accessibility between WT and Top38-KO?*

**Response:** We think that Top3b-TDRD3 complex most likely binds to pol II at their methylated CTD motifs. This is based on earlier work from Danny Reinberg’s lab that TDRD3 can bind arginine methylated CTD of pol II (Sims et al., Science, 2011), as well as on our findings that TDRD3 and pol II co-bind many TSS, exons and enhancers; and TDRD3 and pol II signals strongly correlate at TSS and exons, with correlation efficient of 0.9 and 0.8, respectively.

We should mention that we have attempted to verify a hypothesis from a previous paper by Bedford and colleagues (Yang et al., Mol Cell 2014), which proposed that TDRD3 serves a molecular bridge

13

between Top3b and arginine-methylated histones; and Top3b-TDRD3 is recruited to the c-Myc promoter primarily by the H4R3me2a mark. However, we were unable to detect any enrichment of H4R3me2a ChIP-seq signals at TSS or enhancers at c-myc or other NER genes using two different commercial antibodies. To our knowledge, no lab (including Bedford’s) has published any H4R3me2a ChIP-seq data for any species. Thus, in our revised Discussion, we have only mentioned Top3b-TDRD3 may be recruited by its interaction with methylated Pol II CTD.

We have observed increased H3K27ac at some enhancers of neuronal early response genes, and this increase is similar between Top3b-KO and wildtype mice for most of the genes. as shown in the new Figure S13a-b. Thus, we do not think that difference in H3K27ac can account for the reduced levels of pol II at TSS or enhancers in Top3b-KO mice.

We have performed FAIRE-seq experiment to examine whether chromatin accessibility is altered in brains of Top3b-KO mice. However, the experiment has not been successful due to technical difficulty. We were able to use FAIRE-seq to show that chromatin accessibility is different at some loci in Top3b-KO Drosophila heads (unpublished). We hope to examine this issue in future studies.

- *The authors state Top38 is required for global neuronal activity-dependent transcription. The authors show reduced EP300 in Top38-KO with FC vs WT with FC (Fig 7d), but was genome-wide correlation analysis performed to examine this? H3K27ac, H3Kme marks, FAIRE, etc.*

**Response:** We have genome-wide correlation analysis of H3K27Ac, which is a substrate of EP300 acetyl-transferase, and is known to be induced at neuronal activity regulated enhancers by neuronal activation. We observed that FC induced genome-wide increase of H3K27Ac is comparable between Top3b-KO and wildtype mice (new Fig. S13a-b). However, there is modest reduction of H3K27Ac at some enhancers and TSS of specific neuronal early response genes (Fig. S13c, d). The data suggest that the major effect of Top3b is on Pol II-mediated transcription, and its effect of H3K27ac should be minor and indirect.

We did not observe genome wide reduction of H3Kme signals in Top3b-KO mice, so that we did not include the analysis in the paper. Our FAIRE-seq experiment was not successful, and we will try to use other assays in future studies.

- *Fig. 6a “Top 10 negative MeSH terms for Pol II changes at TSS no Fear” and Fig. 7a “Top 10 negative MeSH terms for Pol II changes at exons with fear”: easier to interpret data comparing TSS vs exons no fear OR comparing TSS vs exons with fear. Perhaps “..TSS fear (S10a)” and “..exons no fear (S9a)” should be included in Fig.6/Fig.7 respectively, rather than left in supplemental?*

**Response:** We have combined the figures 6,7,8 into two figures, as recommended by Reviewer 1. To address the comment by reviewer 2, we have put Mesh term graphs of Pol II data at TSS under both no treatment and FC in Figure 6a and 6b so that readers can compare easily the Mesh terms under both conditions in the regular figure. We have also put Mesh term graphs of Pol II data at exons under both no treatment and FC in Supplemental figure (Fig. S11a and 11b), to save space for the main figure. The overall results from TSS and exon Mesh Graphs are similar—they all implicate schizophrenia and dementia as Top 10 mesh terms. The reason that we put TSS graph in the main figure is that the signals

14

to noise ratios are higher than those in exons, so that the overall Z-scores are also higher.

- *Fig. 7 “(f) A graph shows examples of reduced pol II signals (EP300 and DTNBP1), and reduced induction of Pol signals by FC (BDNF and RGS4), in Top38-KO mice under FC” – how and why were these examples chosen? Were all genes (the other 19 genes from Fig.7d and Fig.7e) also examined in this context?*

**Response:** Reviewer 1 asked us to combine Figures 6, 7, and 8, so that the manuscript can have fewer figures. To address his/her comments, we have removed the old Figure 7f, to save space.

For reviewer 2’s information, we have used graphs and bedgraphs to further analyze ChIP-seq signals for all the schizophrenia-related genes that have at least 20 Pubmed citations, and are significantly reduced in Top3b-KO mice under fear conditioning (p<0.05), or their FC-induced increase is significantly reduced in Top3b-mice. Because of space, we showed representative graphs for those genes that exhibit the most obvious reduction of Pol II signals in Supplemental figures.

*Discussion:*

- *Top38 mutations are mentioned throughout manuscript, e.g.: “Our data suggest that Top38 is crucial for normal brain function and mental health; and that disruption of NADT could be one mechanism by which Top3b mutation causes mental and cognitive disorders.” The authors do not present Top38 mutant data here. Re-word to reflect Top38 deletion. Do the authors have data on Top38 mutants and their role in neuronal activity-dependent transcription? Unreasonable to suggest that Top38 mutations disrupt neuronal transcription without data to back it up (either from authors laboratory or referenced from other research groups).*

**Response:** We have gone through the text, and replaced the word “mutation” with “deletion” when we describe Top3b inactivation in mouse. We still used the word “mutations” when describing human genetic data, because both deletion and *de novo* single nucleotide variants of Top3b gene have been associated with schizophrenia and autism.

- *“Indeed, etoposide, a Top28 poison that induces DSBs by blocking the topoisomerase from rejoining the broken ends, represses far more genes than it activates [15], arguing that Top28-mediated DSB formation facilitates only a small fraction of genes during NADT” – the number of genes repressed or activated is somewhat irrelevant. Their function however, is. Not fair to dismiss Top28 so readily, given that genes down-regulated in Top28−/− neurons show a strong enrichment for gene ontology terms associated with “neurogenesis,” whereas genes up-regulated in Top28−/− neurons were associated with the GO term “cell division” (Tiwari et al. 2012).*

**Response:** We have removed the statement from the text. We also thank reviewer 2 for alerting us the data in Tiwari’s paper—it allows us to compare our data with those of Top2b, and we were able to find a positive correlation between TSS bound by TDRD3-Top3b and Top2b (correlation coefficient 0.6). The data suggest that TDRD3-Top3b and Top2b may preferentially bind and regulate a common set of genes. We have proposed in Discussion that the two topoisomerases could work together to regulate transcription of the same genes in neurons.

15

*Methods:*

- *“Top3b KO mouse is originally produced at Harvard University by targeted disruption of mouse Top3b in C57BL/6J background [56]. Age-matched male littermates were used in all experiments, whereas female mice were included in some behavior tests”:*

*Are the Top38-KO male or female? Specify! Indicate where females were used; were they used because the Top38-KO in those tests were female? Light-dark box test was developed with male mice (Bourin and Hascoët 2003). Perhaps the females displayed higher anxiety and/or other behaviour differences? Have male vs female been compared in the tests where females were used? The stage of reproductive cycle, where there are fluctuations in both oestrogens (e.g., 178-oestradiol) and progestins (e.g., progesterone), and/or hormone concentrations would need to be considered for female mice in behavioural tests – especially the Morris water maze where testing was done across multiple days.*

**Response:** Top3b-KO mice have normal sex ratios. We used females in some behavior tests in order to increase the statistical powers. We have now indicated the number of male and female mice in different behavior tests in Methods.

In the tests that used the females, mice of both sexes do not show obvious difference, which include the light-dark box test. So, we just showed data of total mice in the figure.

We understand that reproductive cycles can affect behaviors of female mice in certain behavior tests. Thus, we only used male mice in Morris water maze, context discrimination, and open field.

- *Elevated plus maze: were mice placed in the maze facing the open or closed arm? Differences can arise if mice are placed facing open vs closed arms. Why are the closed arm walls smaller than standard? (2.5cm vs 15.25cm (Walf and Frye 2007)). Has the maze been validated prior to this study? i.e. have mice that have received either anxiolytic or anxiety-causing pharmacologicals been subjects of the maze?*

**Response:** In elevated plus maze test, mice were placed in the center where the two arms cross. This give the test mouse an equal opportunity to go to either arms. We have described this in the Method.

The 2.5cm arm length was a typo, which should have read 30cm instead. (The wall height in open arms is 2.5cm.) We have corrected the mistake. Our institute has published other manuscripts using this apparatus and procedures to successfully identify differences in anxiety-like behavior in experimental groups of mice (TP Stringer, Transl Psychiatry 2015).

We have not used mice that have received anxiolytic or anxiety-causing drugs in elevated plus maze test, but have examined MK801-treated mice in open field test (Appendix Figure 1). These experiments are time consuming, and we hope to address this issue in future studies.

- *Anxiety test: was it feasible/possible to carry out heart-rate monitoring on the Top38-KO mice as a measure of anxiety? And were cortisol/epinephrine levels measured in Top38-KO mice post-behavioural tests?*

**Response:** Dissecting behavioral vs physiological responding to stressors is an interesting idea. A recent optogenetic publication (Kim SY 2013) showed that unique output projections from the bed nucleus of

16

the stria terminals to either the lateral hypothalamus or parabrachial nucleus selectively modify behavioral anxiety-like measures in the elevated plus maze versus changes in respiratory rate. This suggestion from the reviewer gets to the question of whether the changes in brain circuitry are global across all inputs and outputs to stress-sensitive brain regions, or whether there are circuit-specific alterations. We feel that this type of circuit/systems level question is well outside the scope of the present manuscript.

- *Morris Water Maze: “4 hrs following the last training, mice were placed in water without platform for the first probe trial.” Should this say 24 hrs? and “when the platform was removed for a 60-sec probe trial after the last day of training” – Ref #59 states a probe trial should be done at least 24 hours after last learning trial if they are intended to assess reference memory. Should both probe trials have been more than 24 hours later?*

**Response:** As discussed in an earlier response, we are now choosing to simply present 4 hr probe test data, since the 24hr time point is affected by the experimental design that retested mice, effectively running animals through extinction training. Testing in trials 2-4 on any given day may reflect a combination of long-term reference memory from trials on previous days, as well as short term and working memory for trial(s) occurring earlier within the same day. A 4-hour interval since the final training trial would be expected to allow for some degree of de novo protein translation and thereby synaptic consolidation to occur, converting “short term” into “long-term” memory (reviewed in JL McGaugh 2000 and ER Kandel 2001). Since we cannot definitively ascribe the group differences to short versus long-term memory, we avoid interpretation here in this experiment on this specific point. Future experiments testing the exact time course of memory impairment in Top3b-KO mice would be interesting, but we feel that that question is beyond the scope of the present manuscript, in which we first establish memory deficits using several hippocampus-dependent behavioral tasks.

- *Three-chamber sociability test: In Ref #60 - “At the end of the first 10 minutes, each mouse was tested in a second 10-minute session to quantitate social preference for a new stranger. A second, unfamiliar mouse was placed in the chamber that had been empty during the first10-minute session.” Why was this not done here?*

**Response:** Social memory is a different phenomenon from drive for social interaction, which we here report. Our behavioral core facility manager and manuscript coauthor Dr. Ross McDevitt has extensive experience with mouse behavior in general and 3-chamber sociability in specific. He has found that, although the initial sociability phase of the experiment is highly reliable, the additional social recognition experiment phase as suggested here is not reliable in control mice. He has found that about one third to one half of the time, an entire cohort of wild-type control mice will fail to show preference for social novelty. This result appears to be independent of the experimenter, age of mice, and lighting conditions. In speaking confidentially with a member of a lab in the extramural research community with extensive experience in mouse behavioral tests of learning and memory, this unreliability was confirmed in their lab as well. The unreliable nature of mouse behavior in general is widely known (eg, JC Crabbe 1999) and because of this, we are making a conscious choice at our institute to run and publish behavior tests that we have greater confidence can be reproduced and replicated.

17

- *MRIs for ventricular enlargement (a feature of schizophrenia) were done at 2-3 months. Is this time point too early? Perhaps the authors would have seen statistical significance if MRIs were performed at 6 months?*

**Response:** We chose the 2-3 month time point because when we performed brain dissections, we have visually seen that ventricles of Top3b KO mice appear to be enlarged. In addition, we have also observed significant reduction in adult neurogenesis during this period, which could account for the enlargement of ventricles. It is unfortunate that the results show not significant difference. Because this result is not a big part of the paper, we hope to address this issue when we have more mice available.

- *“Newborn neurons” – X hours after birth? P1? P2?*

**Response:** We used “newborn neurons” here to refer newly developed neurons for mice within 1 month old. We have added this definition in Methods.

- *For “Stereotaxic surgery and viral vector injections”, what age were the mice used? Ref #63 says 5 weeks? Males? Females?*

**Response:** We have added a statement indicating that we used male mice between 4-5 weeks old for viral injection experiments.

- *Similarly, for BrdU injections – age/sex of mice? Also, BrdU was administered i.p. for 3 days to label adult neural SCs in the brain. Could it have been administered i.v. to increase the chances of BrdU crossing BBB? Only 20 positive BrdU cells in WT... poor route of administration? Clearance of BrdU before it got a chance to label adult neural SCs?*

**Response:** We have added a statement in Methods that we used male mice of 1 month old for BrdU injections. We performed the i.p. (intraperitoneal) injections, because it is very commonly used for BrdU assay in studies of adult neurogenesis (Zhang et al., Brain Res Bull., 2018; Wang et al., Front Aging Neurosci, 2018; Madrorial., et al., PlosOne, 2010; Noguchi et al., eLife, 2019).

The number of BrdU positive cells observed by us (20) is well within the range of other studies, which typically observed between 4-80, from different published papers (Zhang et al., Brain Res Bull., 2018; Wang et al., Front Aging Neurosci, 2018; Madrorial., et al., PlosOne, 2010; Noguchi et al., eLife, 2019). So, we think that our BrdU labeling is as efficient as other studies, and can detect the difference between wildtype vs. Top3b-KO mice.

- *Sample sizes? – Figures 4, 6 (and presumably Figure 7): “The data are from 4 pairs of untreated mice, and 5 pairs of FC-treated mice” – n = 4-5 per group. Is this sufficient? Individual mice in each group show highly variable measurements. What are the n numbers for the behavioral tests – is each point in the graphs in Fig.1 and Fig.2 an individual mouse? Or an individual trial (e.g. 12 trials from 4 mice?)*

18

**Response:** The number of 4-5 mice per group should be sufficient, as the difference observed in our hands is statistically different (p<0.0-5). We often see publications with only 2 or 3 replicates of ChIP-seq.

Each point in the graphs represents result from an individual mouse. We have now indicated this in the Figure legend.

*Inconsistencies in labeling etc:*

- *“Top3b” instead of “Top3β” throughout the manuscript.*

**Response:** We have now used *Top3β* throughout the text.

- *Fig. 4e: the corresponding main text has mislabelled this as 3e - “Among them, 31 (69%) also showed at least 1.5-fold increase (Fig. 3e). In contrast, only 3 of these 31 genes showed a significant increase in Top3β-KO mice (Fig. 3e and S4a)”*

**Response:** We have corrected the mislabeling. Because of Reviewer 1’s request, Figure 1 and 2 are combined. Thus, the rest of the figure citations are all changed accordingly.

- *Fig. 5: main text referring to 5d and 5e needs to be clarified – 5d is not mentioned at all, and there’s a discrepancy between numbers in Fig.5e (exons and TSS in blue bars) and corresponding text.*

**Response:** We have now cited these figures (new Fig. 4e and 4f), and corrected the discrepancy between numbers and the text.

- *Fig. 5d: “We also noted that about 2400 genes (10% of total) showed reduced Pol II signals at exons (>1.5-fold, p<0.05) in response to FC in WT mice (Fig. 5d); and this number was only slightly different in Top3β-KO mice (2100; 9%)” – use exact numbers from data: 2373, 2088.*

**Response:** This part of the text discusses the results that Top3b-KO mice also have a small number of genes that show reduction of Pol II signals in response to neuronal activation by FC. We decided to remove this part to save space. This is because we have added more data to the revised manuscript, resulting in the length of the text over the limit of Research Article. We think that this part can be removed without affecting the main conclusion of the paper. The readers can see these numbers and percentages in the graph and make their own conclusions.

We should mention that we found an error in our statistical analyses of pol II ChIP-seq data, so that we have over-estimated the number of genes that have significant difference. We have corrected the mistake in the revised version. The new analysis showed that the number of genes with significant difference are fewer than the previous version.

- *Fig. 5g: two percentages in the overlapping part of the Venn diagram? Explained in the main text but confusing if looking at figure alone. Re-draw Venn diagram to reflect the specifics of these percentages. “49%” is not referred to in the main text?*

19

**Response:** We have now re-drawn the Venn diagram (new Fig. 5d), by putting a connecting underline below each pair of the two percentages that should be compared. Hopefully, this will make clear to readers how to compare the two percentages in the overlapping part to that in the non-overlapping part. We have also cited the percentages in the overlapping parts of the Venn graph.

We should mention that the percentages in the new figures are changed, because we corrected an error in our statistical analyses in the previous version.

- *“Furthermore, TDRD3 signals at TSS have a strong negative correlation with reduced Pol II signals in Top3β-KO mice vs. those of wildtype mice under FC (r=-0.67) (Fig. 5i)” – this is mislabelled – should be Fig. 5k.*

**Response:** This figure has been moved to a Supplemental S10d in the revised paper. We have corrected the mistake.

Finally, we would like to thank both reviewers for their thoughtful comments and valuable advice on how to improve the manuscript. We believe that the new data based on these suggestions have made this paper much more interesting and convincing. We hope that it can now be accepted for publication.

20

Appendix Figure 1

Top3b-KO mice exhibit a strong trend of increased locomotor

activity in response to MK-801 treatment by open field test

a	b

c	d

**Legend:** MK 801 administration increases locomotor activity in both wild-type and Top3b-KO animals in open field test. It has been shown that some schizophrenia mouse models exhibit enhanced locomotor activity in response to this drug (Ayhan et al., Mol. Psychiatry, 16:293, 2011; Ibi et al., Behav. Brain Res. 2010).

Four locomotor activity was measured in a time-course experiment, and their averages, error bars, and statistical difference (p-values; Student T-test) are shown in each graph. (a) Total distance traveled; (b) absolute turn angles of the animal; (c) rotation of the animal body; and (d) maximum speed. After 3 days of handling, the animals (8 WT male and 10 Top3bKO male mice) were put in a 34-cm open field for 30 min in habituation followed by brief handling and another 30 min in the open field. After 24 hours, the animals were put in the open field cage for 30 min habituation (pre-drug time) where their activity was recorded, after which they were injected with MK801 (0.15 mg/kg, i.p.) in physiologic solution and immediately returned to the open field. Activity was subsequently recorded for 90 minutes. An overhead camera was used with ANY-Maze software to score frequency and duration of each parameter detected in the test subject mouse. Both wildtype and Top3b-KO mice showed MK-801 induced increase in the locomotion activity at different time points. Comparisons of 5-minute bins using T-test showed that at about 50 minutes post-injection, differences between WT and Top3b-KO mice were significant at some time points (b, c; p<0.05), and almost significant at several others (a-d; 005<p<0.10). However, differences at most of the time points are not significant(NS) (p>0.05).

**Reviewer comments, second version:**

Reviewer #1 (Remarks to the Author):

The authors have satisfactorily addressed the concerns I raised. While I don't have additional comments, I do feel the authors should actually include text in their manuscript regarding the mention of variations in PolII ChIP-seq signals between animals. This is an important point for readers working in this area, and I think a full disclosure of data variability needs to be clearly stated.

As they state in their rebuttal:"We have observed between-animal variability in Pol II ChIP signals, but do not have a good explanation for the underlying mechanism. We also noticed that that there is also between-animal variability for both WT and Top3b-KO mice in the fear behaviors (freezing time) in response to fear conditioning treatment (between 20-80 sec). This variation has also been observed by many other
studies. One possibility is that the hormonal levels of the animals are different."


Reviewer #2 (Remarks to the Author):

The authors have satisfactorily addressed the majority of my concerns. Towards this. they have provided new data, made extensive changes to the presentation of findings as advised and further provided explanations as required. The manuscript is now suitable for publication.

**Author rebuttal, second version:**

*Reviewer #1 (Remarks to the Author):

The authors have satisfactorily addressed the concerns I raised. While I don't have additional comments, I do feel the authors should actually include text in their manuscript regarding the mention of variations in PolII ChIP-seq signals between animals. This is an important point for readers working in this area, and I think a full disclosure of data variability needs to be clearly stated.

As they state in their rebuttal:"We have observed between-animal variability in Pol II ChIP signals, but do not have a good explanation for the underlying mechanism. We also noticed that that there is also between-animal variability for both WT and Top3b-KO mice in the fear behaviors (freezing time) in response to fear conditioning treatment (between 20-80 sec). This variation has also been observed by many other
studies. One possibility is that the hormonal levels of the animals are different."*

**Response:** As requested by the reviewer, we have added a brief statement in the Results to discuss variations in our Pol II ChIP-seq signals and fear behaviors between animals.  *Reviewer #2 (Remarks to the Author):

The authors have satisfactorily addressed the majority of my concerns. Towards this. they have provided new data, made extensive changes to the presentation of findings as advised and further provided explanations as required. The manuscript is now suitable for publication.*

**Response:** We thank both reviewer 1 and 2 for supporting publication of our manuscript. Their comments and constructive suggestions have significantly improved the quality of our work.

**Reviewer comments, third version:**

**Author rebuttal, third version:**
